# Cryptic, Sympatric Diversity in Tegu Lizards of the *Tupinambis teguixin* Group (Squamata, Sauria, Teiidae) and the Description of Three New Species

**DOI:** 10.1371/journal.pone.0158542

**Published:** 2016-08-03

**Authors:** John C. Murphy, Michael J. Jowers, Richard M. Lehtinen, Stevland P. Charles, Guarino R. Colli, Ayrton K. Peres, Catriona R. Hendry, R. Alexander Pyron

**Affiliations:** 1 Science & Education, Field Museum of Natural History, 1400 S. Lake Shore Drive, Chicago, Illinois, 60605, United States of America; 2 National Institute of Ecology, 1210 Geumgang-ro, Maseo-myeon, Seocheon-gun, 33657, Republic of Korea; 3 CIBIO/InBIO (Centro de Investigação em Biodiversidade e Recursos Genéticos), Universidade do Porto, Campus Agrario De Vairão, Rua Padre Armando Quintas n°7, 4485-661 Vairão, Portugal; 4 Department of Biology, 931 College Mall, The College of Wooster, Wooster, Ohio, 44691, United States of America; 5 Department of Biology, Howard University, Washington, District of Columbia, 20059, United States of America; 6 Departamento de Zoologia, Universidade de Brasília, 70910-900, Brasília, DF, Brasil; 7 Department of Biology, The George Washington University, 2023 G St. NW, Washington, District of Columbia, 20052, United States of America; University of Innsbruck, AUSTRIA

## Abstract

Tegus of the genera *Tupinambis* and *Salvator* are the largest Neotropical lizards and the most exploited clade of Neotropical reptiles. For three decades more than 34 million tegu skins were in trade, about 1.02 million per year. The genus *Tupinambis* is distributed in South America east of the Andes, and currently contains four recognized species, three of which are found only in Brazil. However, the type species of the genus, *T*. *teguixin*, is known from Bolivia, Brazil, Colombia, Ecuador, French Guyana, Guyana, Peru, Suriname, Trinidad and Tobago, and Venezuela (including the Isla de Margarita). Here we present molecular and morphological evidence that this species is genetically divergent across its range and identify four distinct clades some of which are sympatric. The occurrence of cryptic sympatric species undoubtedly exacerbated the nomenclatural problems of the past. We discuss the species supported by molecular and morphological evidence and increase the number of species in the genus *Tupinambis* to seven. The four members of the *T*. *teguixin* group continue to be confused with *Salvator merianae*, despite having a distinctly different morphology and reproductive mode. All members of the genus *Tupinambis* are CITES Appendix II. Yet, they continue to be heavily exploited, under studied, and confused in the minds of the public, conservationists, and scientists.

## Introduction

Tegus, lizards of the genera *Tupinambis* Daudin and *Salvador* Duméril and Bibron are important in Neotropical ecosystems as predators, scavengers, and seed dispersal agents [1, 2]. They are hunted for skins and meat by thousands of indigenous and local people, and are important sources of protein and income [3]. Tegus make up 1–5% of the biomass harvested by the local populations [4, 5]. However modest the indigenous harvest may appear, the numbers in trade suggest tegu lizards are being harvested at a dramatic rate. Between 1977 and 2006, there were 34 million in trade, with the primary end product being cowboy boots [6]. Tegu lizards are to varying degrees habitat generalists using forests as well as savannas, climbing trees, burrowing, and using riparian, mangrove, and human-modified habitats [2]. Their populations must be substantial and resilient to sustain an annual harvest that averages 1.0–1.9 million individuals per year for thirty years [6]. By any estimate, tegus are an ecologically and economically important clade of lizard. These widespread, heavily exploited species are classified as species of Least Concern based upon their distribution, abundance, and an absence of evidence of population decline.

Imagine, however, that one of these species, *Tupinambis teguixin*, was in reality a collection of cryptic species some of which are living in sympatry at some locations. By definition, cryptic species are morphologically similar to the human eye, but genetically distinct. Such populations have commonly been historically classified as a single species. The phenomena of cryptic species is well known and frequently encountered when detailed studies involving morphology, genetics and ecology are undertaken [7]. Therefore their discovery should not be unexpected, except perhaps for the fact that these lizards are extensively used by humans and have been the subject of hundreds of scientific studies.

The genus *Tupinambis* contained seven species until Harvey *et al*. [8] revalidated *Salvator* Duméril and Bibron for *S*. *duseni*, *S*. *merianae*, and *S*. *rufescens*. The generic split was subsequently supported by molecular work [9]. *Salvator* inhabits much of South America east of the Andes, and they share a suite of traits (a complete row of supraocular granules, divided caudal annuli alternating with complete annuli, a round pupil, keeled proximal subcaudals, and usually a divided loreal) distinguishing them from the sometimes sympatric *Tupinambis*. Thus, four species, *T*. *longilineus*, *T*. *palustris*, *T*. *quadrilineatus*, and *T*. *teguixin*, remain in Daudin’s genus. One of these, *T*. *palustris*, is poorly known and its status seems uncertain. Two of us (GRC, AP) are currently working to clarify its relationship to the other species in the genus.

*Tupinambis* is distributed from the Chocó of Colombia eastward to northern Venezuela, (including the Isla de Margarita, Trinidad and Tobago) and the Guianas southward into Amazonia, and the Cerrados of eastern Bolivia [8]. Three of the four species, *Tupinambis longilineus* [10], *T*. *palustris* [11], and *T*. *quadrilineatus* [12] have poorly understood distributions centered in Brazil. One species, *T*. *longilineus*, is known to use open, sub-montane tropical rainforest along rivers as well as disturbed areas. Another, *T*. *palustris* is apparently restricted to wetlands in the vicinity of the type locality at Usina Hidréletrica Três Irmãos, in the lower Tiete River, between Aracatuba and Pereira Barreto in the state of São Paulo, Brazil. *Tupinambis quadrilineatus* is endemic to the savannas of central Brazil [13, 14, 15], however Langstroth [16] suggested it may also occur in Bolivia. These three species have all been described since 1995. The range of *T*. *teguixin* is thought to overlap the distribution of all three congeners, and has a range that encompasses that of the entire genus, or nearly so [8].

With a maximum body length of 400 mm [8], *Tupinambis teguixin* is one of the largest terrestrial, and as previously noted one of the most exploited, Neotropical lizards. Yet, its systematics and nomenclature remain poorly resolved, with some authors [3] describing the taxonomy as “tortuous.” Discussing genetic data Fitzgerald *et al*. [3] wrote, “…the split among *T*. *teguixin* from Cuyabeno, Ecuador and Roraima, Brazil was comparable to differences between *T*. *teguixin* and *T*. *longilineus* and even to that between *T*. *rufescens* and *T*. *merianae*.” This would be expected in species composed of multiple lineages and given the two localities are separated by more than 1500 km. However, *Tupinambis teguixin* has been used in hundreds of phylogenetic, ecological, morphological, and physiological studies given its abundance, size, and availability in museum collections and the pet trade, without the systematic work to clarify the status of various populations.

There are two common, but quite contradictory, names applied to *Tupinambis teguixin* in the pet trade and popular literature suggesting differences in coloration, the golden tegu and the black and white tegu. The name "black and white" tegu is also commonly applied to *Salvator merianae* [17]. Beebe [2] was well aware of the ontogenetic and geographic variation in coloration and pattern in neonate and adult *Tupinambis*. He described them as being black above, spotted and blotched on the head and body, and broadly banded on the tail with bright yellow. Beebe described neonates and young as banded from nape to tail tip. In the same paper he noted a pattern of four longitudinal series of white dashes down the back from the nape to mid-tail. An examination of 53 specimens from Suriname [18] and 37 specimens from Brazil and Suriname [10] diagnosed *T*. *teguixin* as having one loreal, all supraoculars in contact with ciliaries, upper temporals smaller than lower ones, enlarged supratemporals, 94–122 scales around mid-body, 21–28 longitudinal rows of ventrals, a total of 10–17 femoral pores, 13–18 lamellae under fourth finger, 29–39 lamellae under fourth toe, a dorsal color pattern with transverse bands or irregularly vermiculated, and a gular region that could be uniform or spotted. However, Colombian, Bolivian, Ecuadorian, Peruvian, Venezuelan, Trinidad, and Tobago specimens were not included in either of the accounts. Here we diagnose the four species supported by our molecular data and associated morphology.

**Fig 1:**
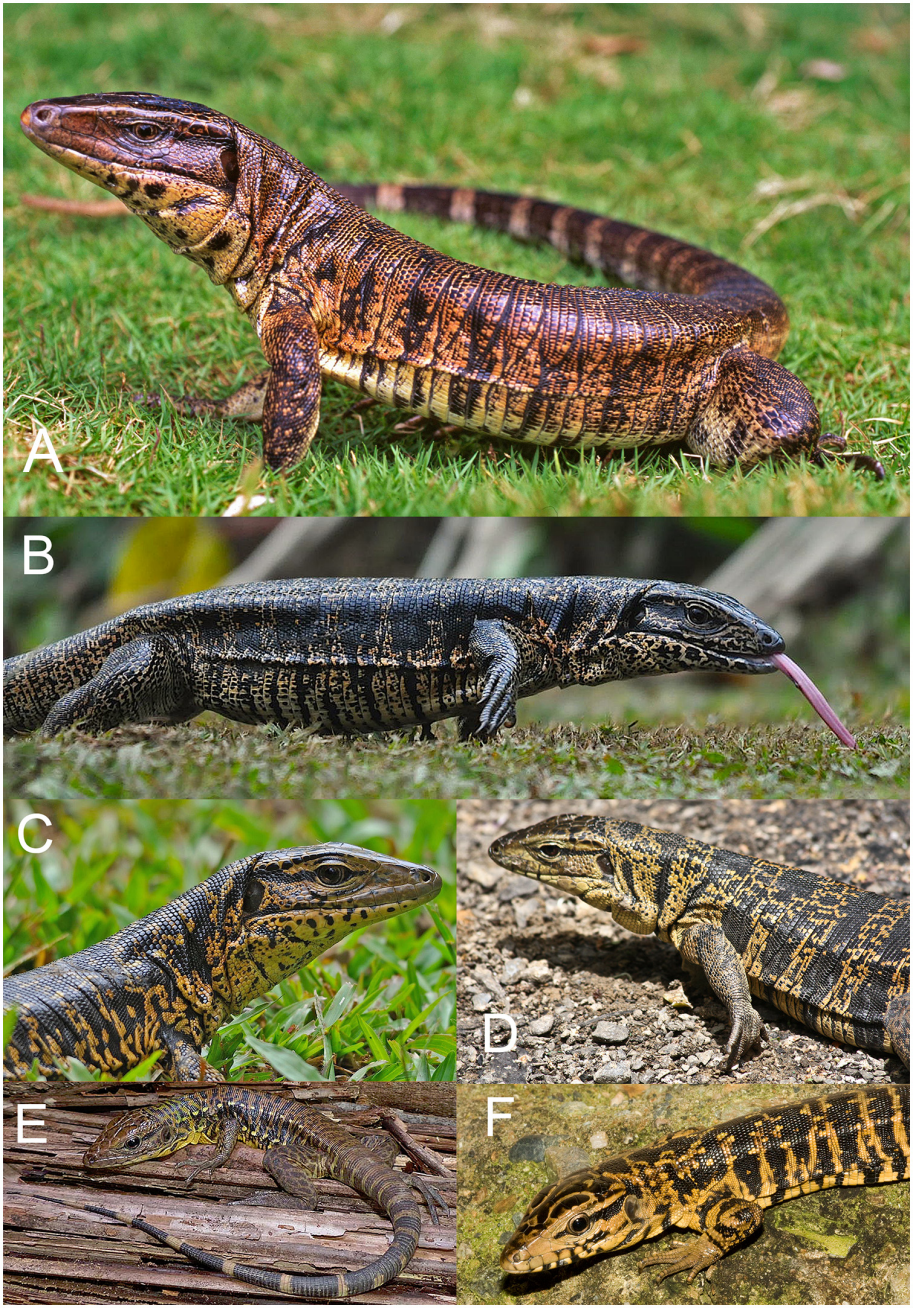
Six specimens of the *Tupinambis teguixin* Group presently considered *Tupinambis teguixin*. (a) Roraima, Brazil (b, c); Guyana (d) Trinidad; (e) Peru, Department Loreto, near the Madre Selva field station, on the Rio Orosa; (f) Tobago. Photographers: (a) GRC; (b, c) Armida Madngisa; (d, f) JCM; (e) Mike Pingleton.

Fig 1 illustrates some of the varied patterns and coloration present in the *Tupinambis teguixin* group. Here, we present a range-wide analysis of molecular and morphological data, strongly supporting the existence of four species-level taxa within what is currently considered *T*. *teguixin*. Morphological data suggest the potential presence of additional species. We discuss the phenomenon and impact of cryptic, widespread species, and offer perspectives for future research.

Confusion over the use of *Tupinambis teguixin* (Linnaeus) and *Tupinambis nigropunctatus* Spix is a long standing problem and closely tied to the *Salvator merianae* entanglement [10]. Hoogmoed and Lescure [19] and Hoogmoed and Gruber [20] considered *Lacerta teguixin* Linnaeus and *Tupinambis nigropunctatus* Spix distinct, but Presch [21] considered them conspecific due to overlapping characters. The nomenclatural problems have been discussed and clarified by Avil-Pires [10] and we have little to add to her discussion. Using photographs of type material, museum specimens and molecular analysis we conclude the following.

A *Lacerta teguixin* paralectotype, the *Seps marmoratus* holotype, and a *Tupinambis nigropunctatus* paralectotype examined for this study all clearly lack the divided loreal, the small rows of granular scales between the supraoculars and ciliaries and the other traits associated with the genus *Salvator*.

Presch [21] designated UUMZ 14 the lectotype of *Lacerta teguixin*, restricted Linnaeus’ type locality of “Indiis” to the vicinity of Paramaribo, Suriname, and placed *T*. *nigropunctatus* as a junior synonym of *T*. *teguixin* because he noted Peters and Donoso-Barros [22] separated the two species based on a divided loreal. Presch [21] found loreals could be single, divide or tripart and that the number of pores, longitudinal and transverse ventral rows, lamellae on the fourth finger and toe, number of vertebral rows, overlapped between *T*. *teguixin* and *T*. *nigropunctatus*. We located UUMZ 13, a badly desiccated specimen of *Tupinambis*. The UUMZ database contained the following information.

*Lacerta teguixin* # 13. Protologue: 1758: 208. Autoreference: 1749, p. 128. Donation: C. Gyllenborg. Depository: UUZM. Preparation: alcohol. = *Tupinambis teguixin* (Linnaeus 1758)

(cf. Lönnberg 1896, no. 14).

Thus, it seems likely this was the same specimen examined by Presch (UUMZ 14) [21] prior to its desiccation, a conclusion previously confirmed by Avila-Pires [10]. No useful data could be obtained from this specimen.

**Fig 2:**
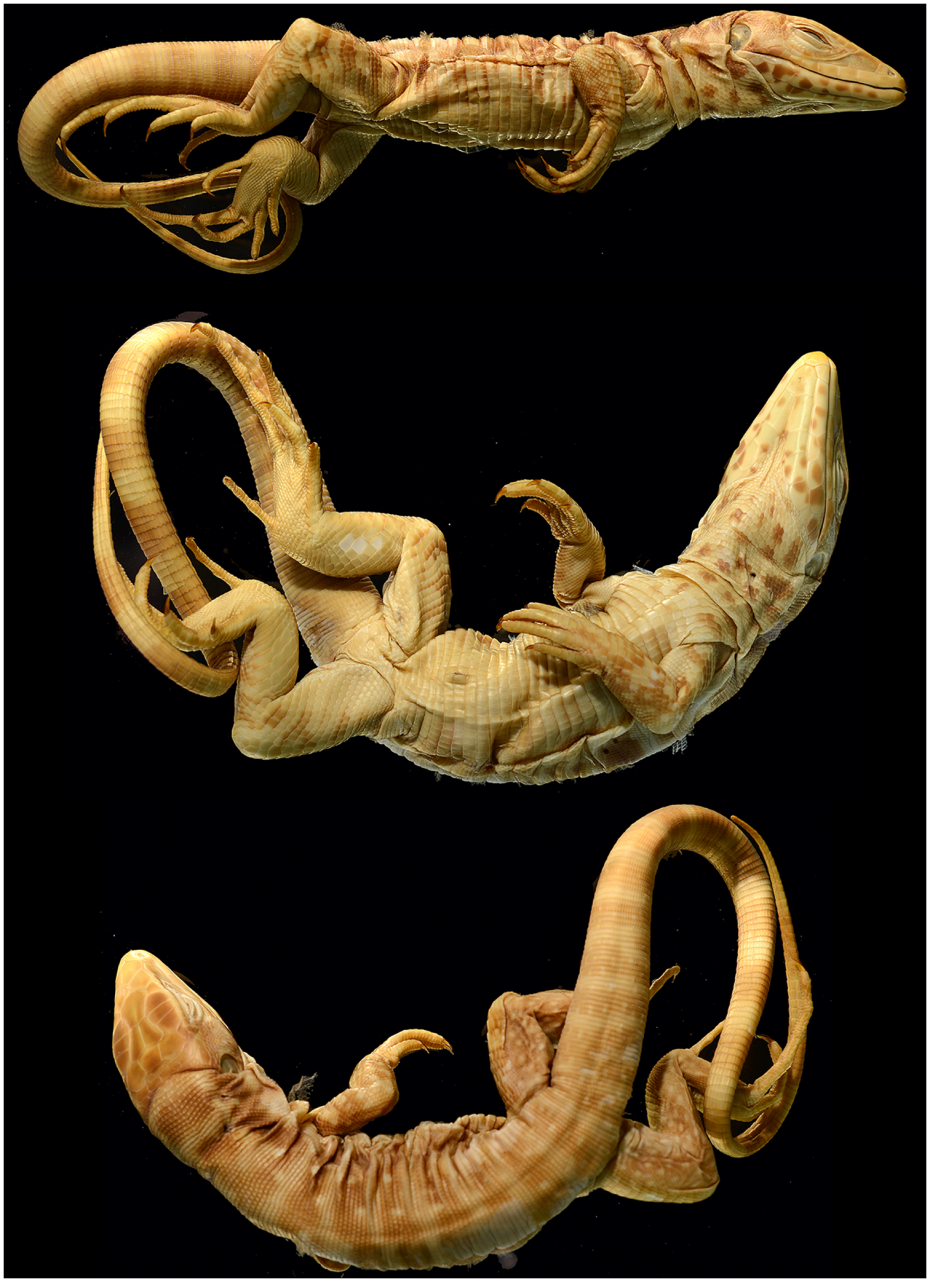
The neolectotype of *Lacerta teguixin* (NRM 121). Photo credit Sven O. Kullander.

A *Lacerta teguixin* paralectotype (NRM 121) examined by us (Fig 2) has five supraoculars, the first is the longest, the second is largest in area, and the fifth contacts two ciliaries. Three occipitals contact the interparietal scale and there are distinct, nearly round spots present on the dorsal surface of hind legs. The specimen has about 114 rows of vertebrals. All traits suggest it is a member of our second clade. However, the NRM lists four paralectotypes of *Lacerta teguixin* (120, 121(2) 123) and scale counts made on other specimens by one of us (AP) suggests clade four may also be represented in this material. Given the above situation, we select NRM 121 as described above and illustrated in Fig 2 as the neolectotype for *Lacerta teguixin*. Presch’s type locality restriction [21] remains appropriate.

**Fig 3:**
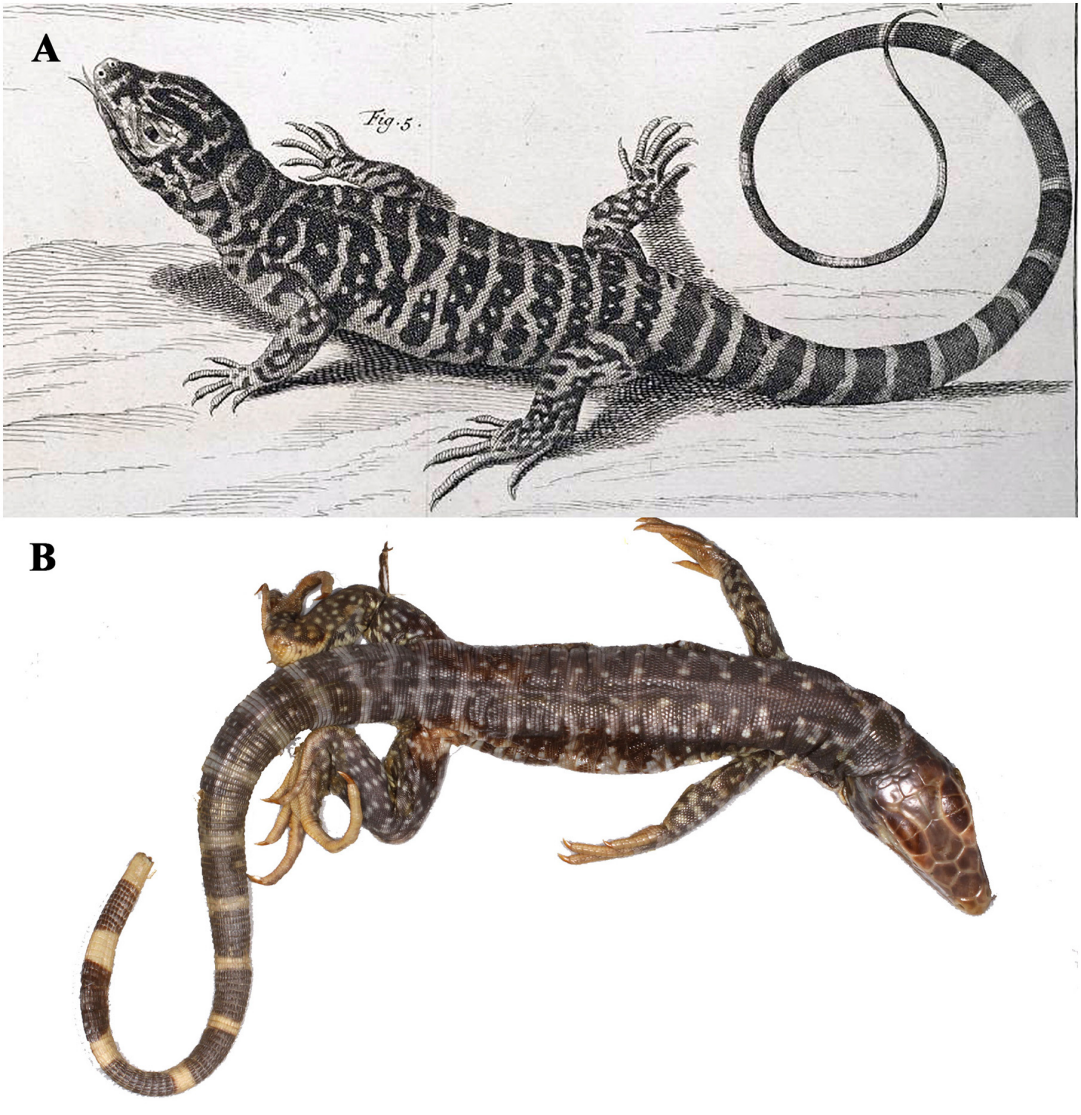
The plate (A) of *Seps marmoratus* from Seba [24] and the specimen (B) (ZMB 849) thought to be the model for the plate. Photo credit Aaron Bauer.

*Seps marmoratus* Laurenti is most likely based upon ZMB 849 [23] a juvenile specimen (Fig 3). The name has long been considered a junior synonym of *Tupinambis teguixin*. The dorsal pattern is composed of wide dark bands separated by narrow light bands and four rows of white spots on the dorsum. It has five supraoculars, first is the longest, the second is the largest in area and the last one contacts two ciliaries. Also, it has 115 or 116 vertebral rows. All are in agreement with our clade two. Here we retain this name as a junior synonym of *Tupinambis teguixin* based upon the data we have.

**Fig 4:**
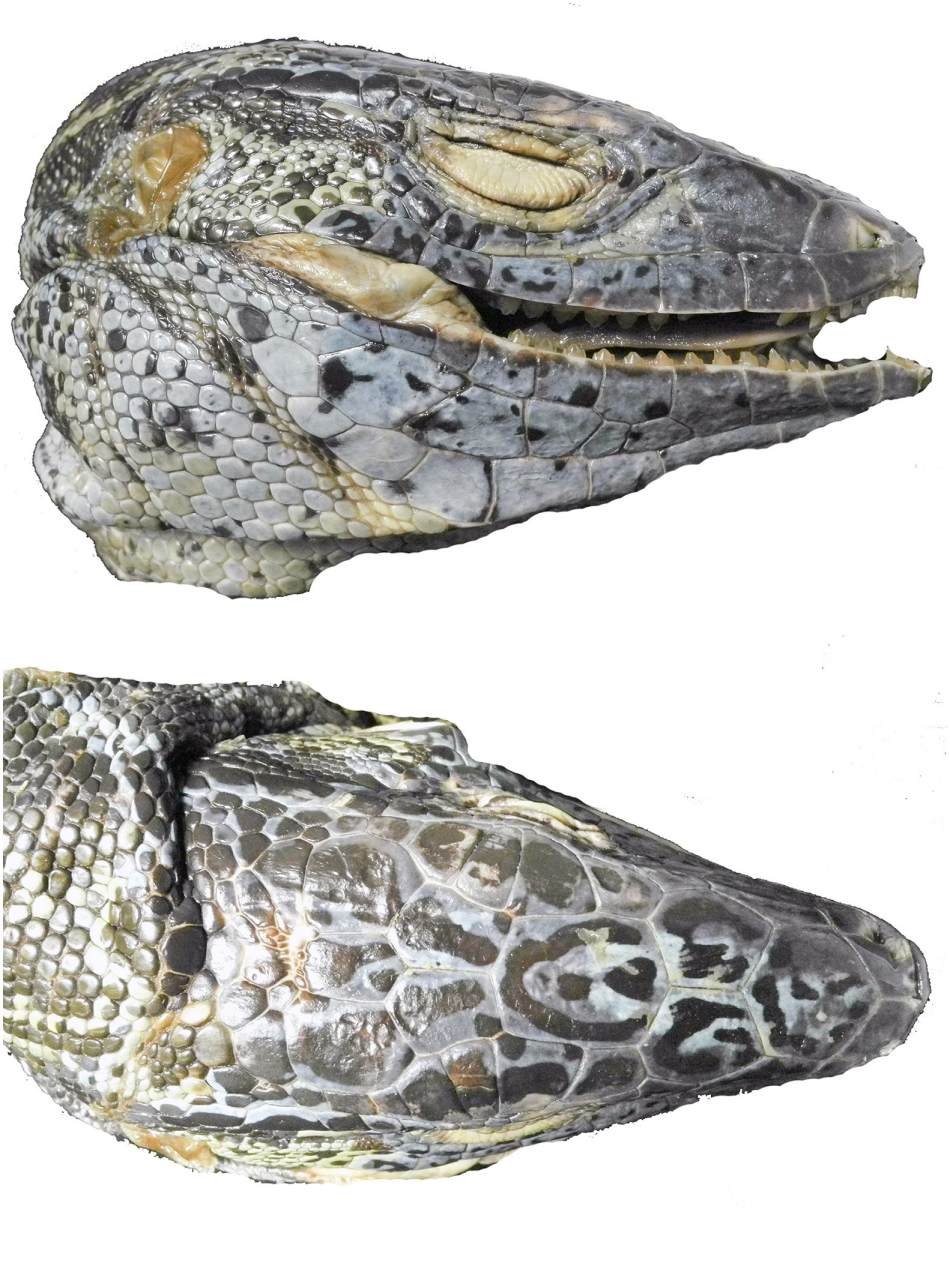
A paralectotype of *Tupinambis nigropunctatus*. Photo credit Michael Franzen.

*Tupinambis nigropunctatus* Spix was based upon five syntypes and all are extant. Hoogmoed and Gruber [20] designated ZMA 629 the lectotype, making ZMA 627, 628, 630, 3208 paralectotypes. They note Spix was unsure of his own classification when it came to distinguishing it from *T*. *teguixin*, and thought it to be either a different species or a female *T*. *teguixin*. Vanzolini [25] interpreted the Spix type locality to be Belém, Para, Brazil. Photos of ZMA 627 (a male) illustrate morphology that agrees relatively well with our clade two (Fig 4). Here we retain *Tupinambis nigropunctatus* Spix as a junior synonym of *T*. *teguixin*.

## Materials and Methods

### Molecular Methods

We gathered tissue samples from existing museum collections from 40 *Tupinambis* and *Salvator*, including 31 *T*. *teguixin*. Using standard PCR and Sanger-sequencing methods, we sequenced fragments of three mitochondrial genes; the 12S rDNA using primers 12SA 5′-AAACTGGGATTAGATACCCCACTAT-3′ and 12SB 5′-GAGGGTGACGGGCGGTGTGT-3′ from Kocher *et al*. [26], 16S rDNA using primers 16SL: 5′-GCCTGTTTATCAAAAACAT-3′ and 16SH 5′-CCGGTCTGAACTCAGATCACGT- 3′ from Palumbi *et al*. [27], and ND4 using primers ND4 5’ CAC CTA TGA CTA CCA AAA GCT CAT GTA GAA GC 3’ and LEU 5’ CAT TAC TTT TAC TTG GAA TTT GCA CCA 3’ from Arévalo *et al*. [28]. These data were combined with all available, vouchered individuals from GenBank for those genes for *Crocodilurus*, *Dracaena*, *Tupinambis*, and *Salvator*, representing the subfamily Tupinambinae, with *Callopistes* representing Callopistinae, following Harvey *et al*. [8] and Ameiva Ameiva as the outgroup.

Sequences were aligned using MAFFT [29] with the default parameters in Geneious (Biomatters, Ltd.). We determined the optimal partitioning strategy for these loci using PartitionFinder [30] using the BIC criterion. We estimated phylogenies using MrBayes3.2.5 [31] using the optimal partitioning strategy [S1 Fig]. We used 2 runs of 4 chains each (three cold, one hot) for 6,666,667 generations, discarding the first 25% as burnin, diagnosed as an Effective Sample Size (ESS) >100 for all parameters [32]. We summarized the posterior distribution using a majority-rule consensus tree, with support estimated as the Posterior probability (Pp) for each node from the sampled trees. Specimen vouchers and GenBank accessions are given in [S1 Table].

Specimens collected were covered by Trinidad and Tobago Forestry Division Special Game Licenses issued to JCM and RML on June 18, 2012 and June 5, 2013.

### Nomenclatural Acts

The electronic edition of this article conforms to the requirements of the amended International Code of Zoological Nomenclature, and hence the new names contained herein are available under that Code from the electronic edition of this article. This published work and the nomenclatural acts it contains have been registered in ZooBank, the online registration system for the ICZN. The ZooBank LSIDs (Life Science Identifiers) can be resolved and the associated information viewed through any standard web browser by appending the LSID to the prefix “http://zoobank.org/”. The LSID for this publication is: urn:lsid:zoobank.org:pub: 40988884-7383-413E-B68D-EDC0778F5A1E. The electronic edition of this work was published in a journal with an ISSN, and has been archived and is available from the following digital repositories: PubMed Central, LOCKSS.

We reviewed the literature and examined illustrations and specimens said to be *Tupinambis teguixin* and *T*. *nigropunctatus* in an attempt to understand the characters various authors have attributed to each name. We also consulted researchers with extensive taxonomic knowledge for their opinions on the status of some names. Taxonomic decisions are best made on the basis of recognizable morphological characters and concordant molecular evidence [33]. Thus, we reconcile geographic genetic variation with meristic and mensural characters from specimens to produce a robust taxonomic estimate with diagnostic evidence from both molecular and morphological data. This integrates all available data, using the General Lineage Species Concept to delimit evolutionarily distinct clades as independent species [34].

### Morphological methods

For this work we examined 335 extant museum specimens for morphological data [S2 Table]. Three previous works [8, 10, 18] provide detailed descriptions for *Tupinambis*. However, some clarification as well as challenges regarding scale and scale arrangement terminology for *Tupinambis* are needed. While we use the terms and characters provided in these papers, we made some adjustments. Some scale counts and characters were found to contain information for distinguishing taxa, but most did not.

Some traditional characters were of limited use because the ranges overlapped extensively. These included vertebral row counts (from the occiput to the row immediately posterior to the hind legs), transvers and longitudinal ventral scale rows counts, and lamellae on the fourth finger and fourth toe counted from the articulation points. Scales around mid-body were counted from one ventral around the mid-body, including scales of all sizes and shapes.

Characters that were more valuable for distinguishing taxa included the length and area of the supraoculars (the longest vertebral axis and the largest area). Hoogmoed [19] described *Tupinambis* as having four supraoculars, and Avila-Pires [10] noted that a fifth scale is present that could be considered a supraocular; Harvey *et al*. [8] described this scale as a circumorbital. Here we follow Avila-Pires [10] and consider the fifth and subsequent scales (if present) to be supraoculars, given their position above the orbit, and contact with ciliaries. The number of occipital scales contacting the interparietal scale is relatively consistent within taxa; usually one or three occipital scales contact the interparietal, but occasionally the occipitals become fragmented or granulate in some specimens.

**Fig 5:**
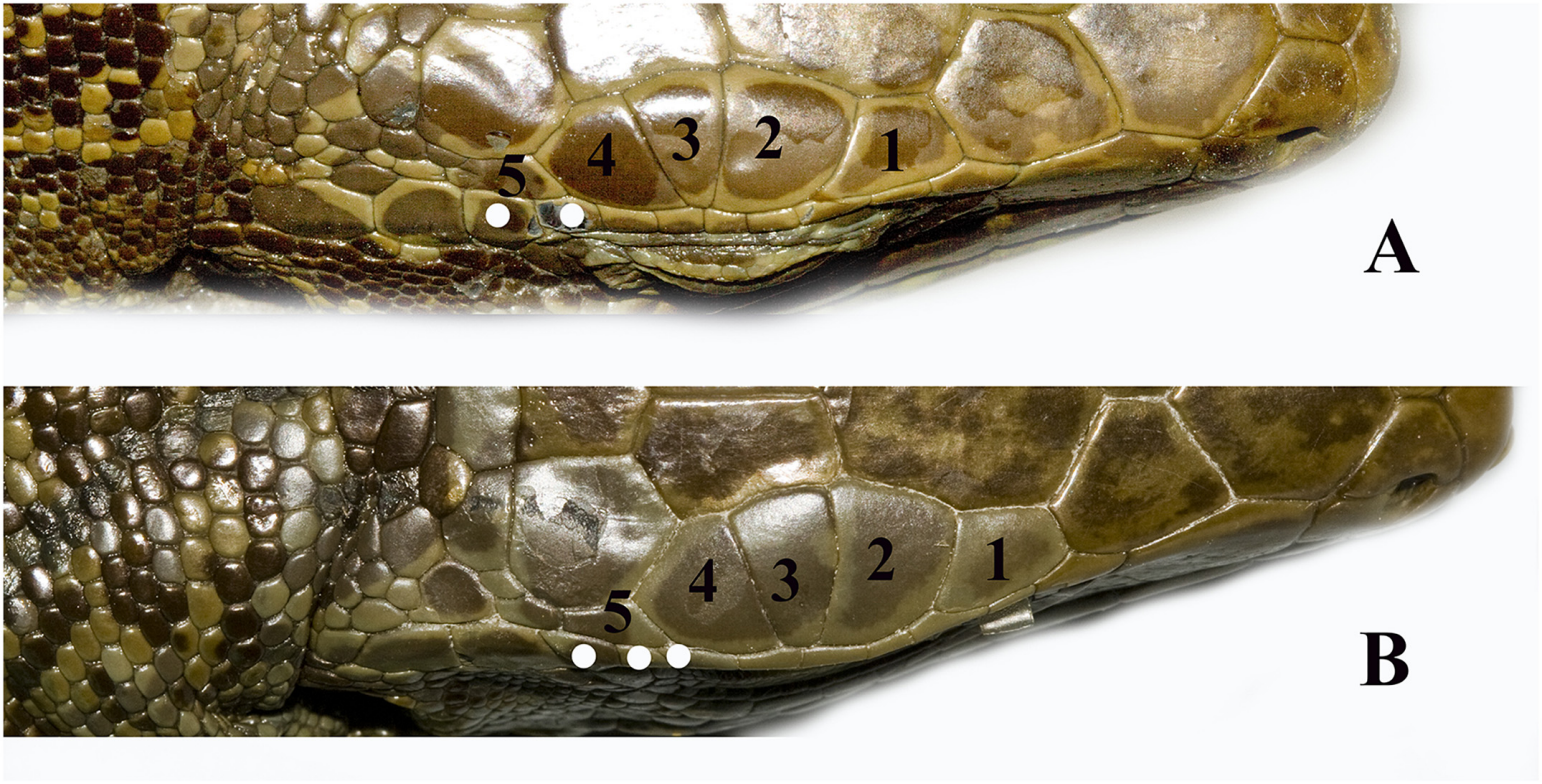
The number of ciliaries in contact with the last supraocular. This is useful for identification since some taxa tend to have two ciliaries in contact with the last supraocular, while others tend to have three. The white markers denote the ciliaries in contact with the last supraocular.

**Fig 6:**
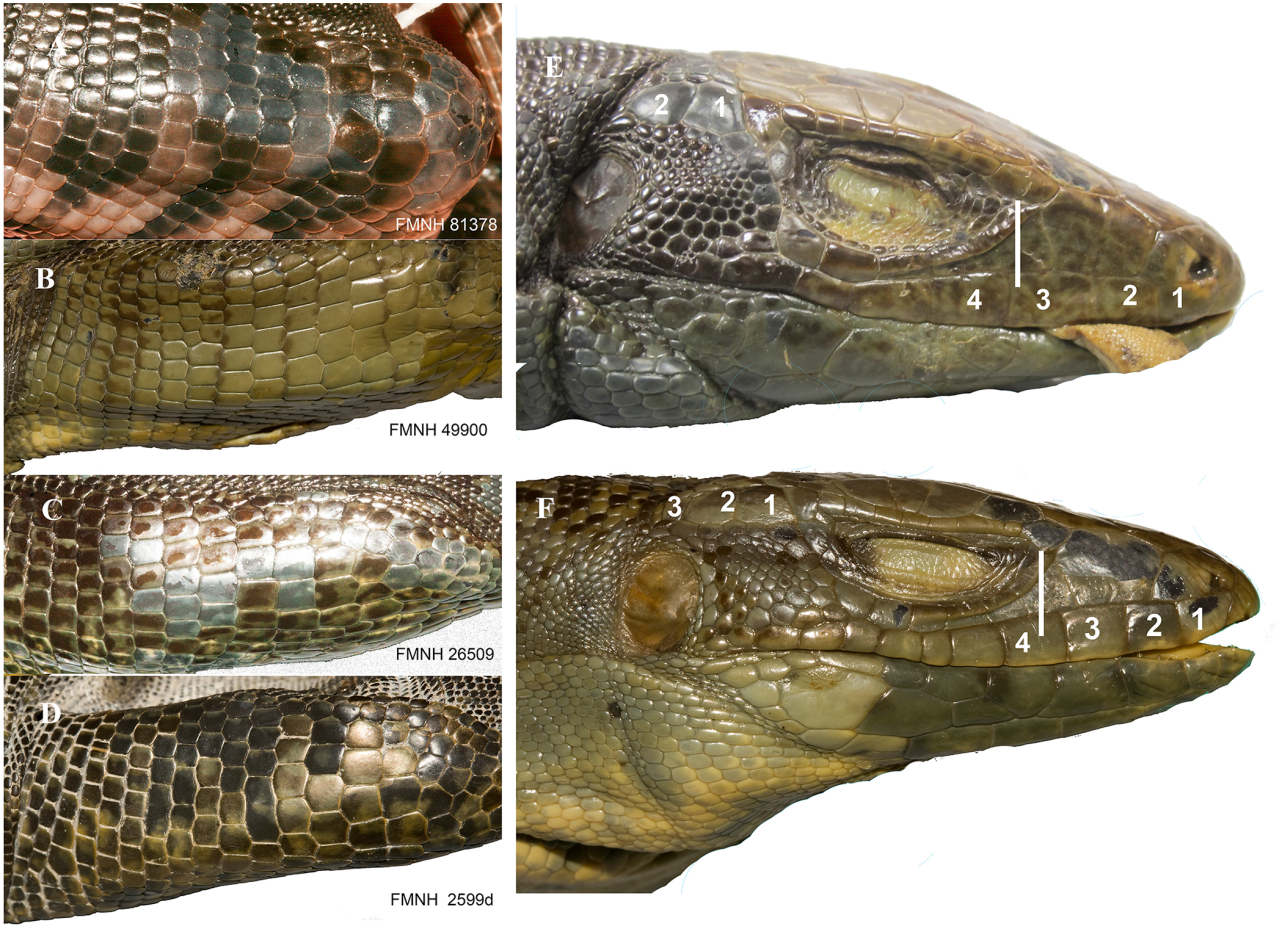
Two traits that are useful in separating the four species of the *Tupinambis teguixin* group. First, the shape and size of the scales on the anterior surface of the femur: (A) *T*. *cuzcoensis*; (B) *T*. *cryptus* (C) *T*. *teguixin* (D) *T*. *zuliensis*. Second, the upper labial under the anterior corner of the orbit (E, F). The inside corner of the orbit is over the third upper labial in *Tupinambis teguixin*, and the fourth upper labial in *T*. *cryptus*. The supratemporals are numbered. *Tupinambis teguixin* (E) usually has two supratemporals and *T*. *cryptus* (F) usually has three supratemporals.

Total number of pores (precloacal + femoral pores) was calculated. *Tupinambis* has a gap between the pre-cloacal pores and the femoral pores. Pores are obvious in males while females tend to have pore-bearing scales with a small pore and a notch extending to the edge of the scale. Pore bearing scales were counted in both sexes. The number of enlarged supratemporal scales was somewhat useful, some taxa tend to have two enlarged supratemporals while others tend to have three. The number of ciliaries in contact with the last supraocular was useful, as some taxa tend to have two ciliaries in contact with the last supraocular, while others tend to have three (Fig 5). The shape and size of the largest scales on the anterior surface of the femur were of some use in distinguishing between taxa (Fig 6A–6D). The position of the anterior inside corner of the orbit (defined as the posterior junction between the first subocular and the first ciliary) over an upper labial was also useful. In some taxa it is over the third upper labial, in others it is over the fourth (Fig 6E and 6F).

Examination of photographs of type material including one of the paralectotypes of *Lacerta teguixin* Linnaeus (NRM 121) as well as *Seps marmoratus* Laurenti, and *Tupinambis nigropunctatus* Spix (ZMA 629) allowed for some comparison of the type material to the data collected from the specimens examined.

**Fig 7:**
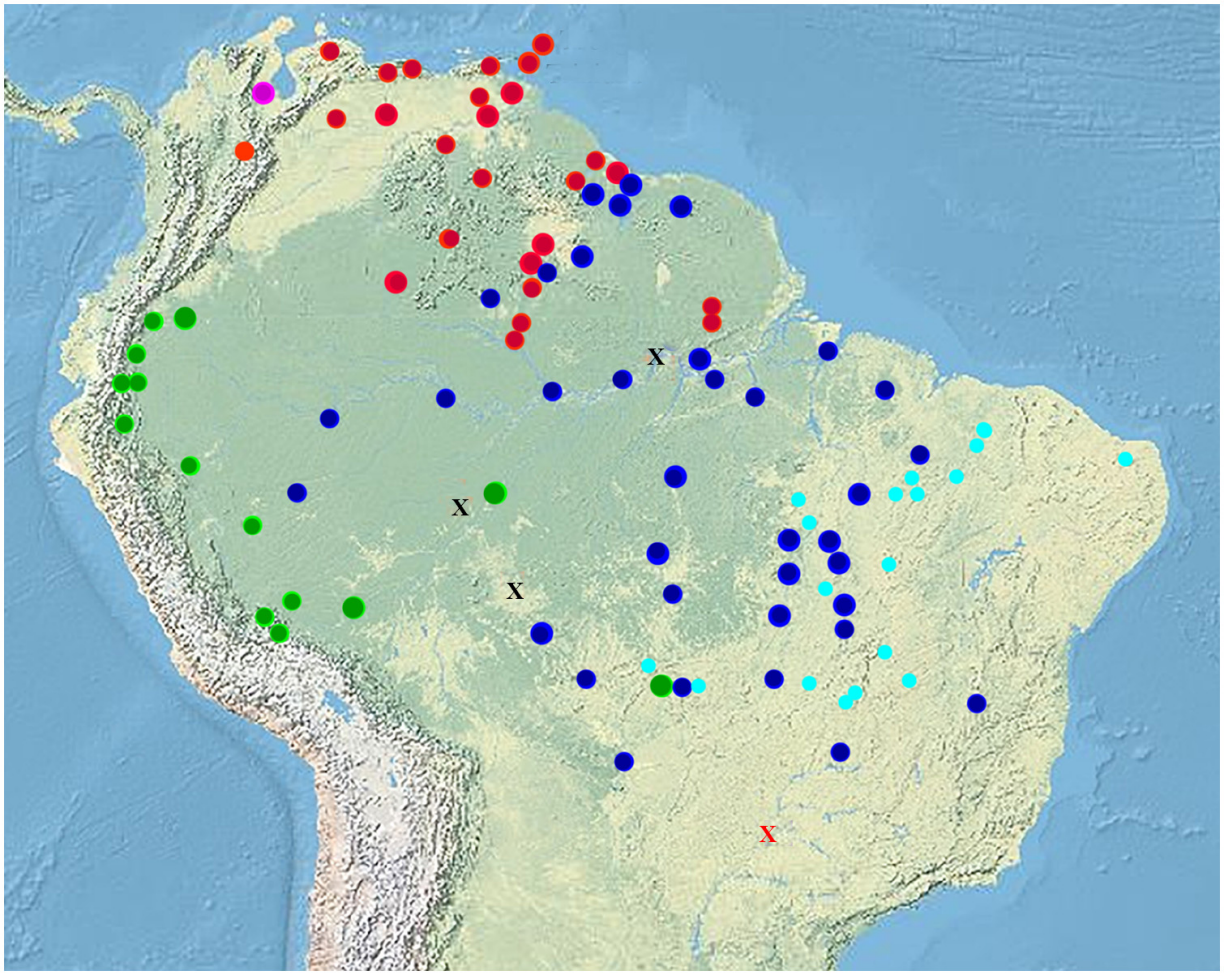
The distribution of members of the genus *Tupinambis*. Large circular markers denote the localities of specimens sampled for DNA. Smaller circular markers denote localities of specimens identified using morphology: Green is clade 1 (*T*. *cuzcoensis* sp. n.), blue is clade 2 (*T*. *teguixin*), purple is clade 3, (*T*. *zuliensis* sp. n.), and red is clade 4 (*T*. *cryptus* sp. n.). The two most northern red circles represent the islands of Tobago and Trinidad respectively. The other markers denote other species of *Tupinambis* not in the teguixin group. Red X = *T*. *palustris*. Black X = *T*. *longilineus*. Aqua blue circles = *T*. *quadrilineatus*.

Geographic methods–Coordinates for museum localities were obtained using the National Geospatial Intelligence Agency’s GeoNames Search website and Google Earth when museum data did not contain map coordinates. Locality data was plotted using Arcview. Fig 7 illustrates localities sampled for DNA and morphology.

Measurements of body and tail lengths were taken to the nearest 1 mm using a ruler and tape measure. Dial calipers were used to measure scale lengths to the nearest 0.1 mm. Values for paired head scales are given in left/right order.

Univariate analyses of morphological data, Student t-tests, principal component analysis, and cluster analysis were applied when necessary. Statistical analysis was done in Excel with QIMacros and cluster analysis and PCA’s were made using DataLab. Because this project was started independently in the USA and Brazil not all data were collected for all specimens. The USA participants collected about 74 pieces of data on the specimens examined, the Brazilian contingent collected about 27 pieces of data on each specimen. About 12 of these traits overlapped. Consequently, sample size in various analyses varies considerably.

We also used the Guided Regularized Random Forest (GRRF) method to assess interspecific differences in meristic counts and determine predictor importance, with R package RRF [35, 36, 37]. In this analysis, we used the following meristic counts: lower labials, upper labials, scales around midbody, vertebral rows, longitudinal ventral rows, transverse ventral rows, 4th finger lamellae and 4th toe lamellae. Prior to implementing GRRF, we imputed 46 missing values (2% missingness) using Multivariate Imputation by Chained Equations (MICE), with package mice [38]. We estimated prediction error based on 100 replicates of 10-fold cross-validation [39] of models with sequentially reduced number of predictors, ranked by importance. When building decision trees in random forests [40], regularization penalizes the selection of new features for splitting when the gain (e.g. decrease in Gini impurity or increase in information gain) is similar to that of features used in previous splits, a method known as Regularized Random Forest (RRF). A GRRF is an enhanced RRF in which the importance scores from an ordinary RF are used to guide the feature selection process of RRF [35, 36, 37].

Several of the specimens used in the molecular analysis were also used in the morphological analysis for *T*. *teguixin*, *T*. *cuzcoensis*, and *T*. *cryptus* sp. n. and the morphological data from those specimens was used to diagnose the four clades; this was not possible for *T*. *zuliensis*. The names established in this paper have been registered at ZooBank.

## Results

### Molecular results

The Bayesian Inferences (BI) analyses converged very quickly, with PSRF~1.0 and ESS>200 for all parameters. Our results are similar to previous phylogenies of Tupinambinae [3, 9, 41]. The subfamily is strongly supported as monophyletic (Pp = 100), as are the genera *Callopistes* (100), *Salvator* (100) and *Tupinambis* (98). The placement of the genera *Dracaena* and *Crocodilurus* is not strongly supported, likely due to the small amount of mitochondrial data available for those species. We find weak support for a clade consisting of, respectively, *Dracaena*, *Crocodilurus*, and *Tupinambis*. We also find strong support for all sampled species, with possible paraphyly of *S*. *rufescens*, which includes two specimens of *S*. *duseni* [3], though this could potentially be specimen mis-identification. Multi-locus nuclear datasets and deeper phylogeographic investigation will be needed to resolve deeper relationships in Tupinambinae and species limits in *Salvator*.

Within *Tupinambis*, we find strong support for a clade of *T*. *longilineus* + *T*. *quadrilineatus* as the sister group to *T*. *teguixin* sensu lato. Interestingly, the *T*. *teguixin* group is not strongly supported as monophyletic (Pp = 63). Within the *T*. *teguixin* group, there are four highly divergent clades that are well-differentiated morphologically (see below). Some of these have been identified already by previous authors [3, 42]. The first clade inhabits the Andean foothills and the western Amazon Basin. The second clade is widespread east of the Andes, in the Cerrado. The third clade appears restricted to the Maracaibo Basin in Venezuela and the fourth clade is primarily on the Guiana Shield and in the eastern Amazon basin.

**Fig 8:**
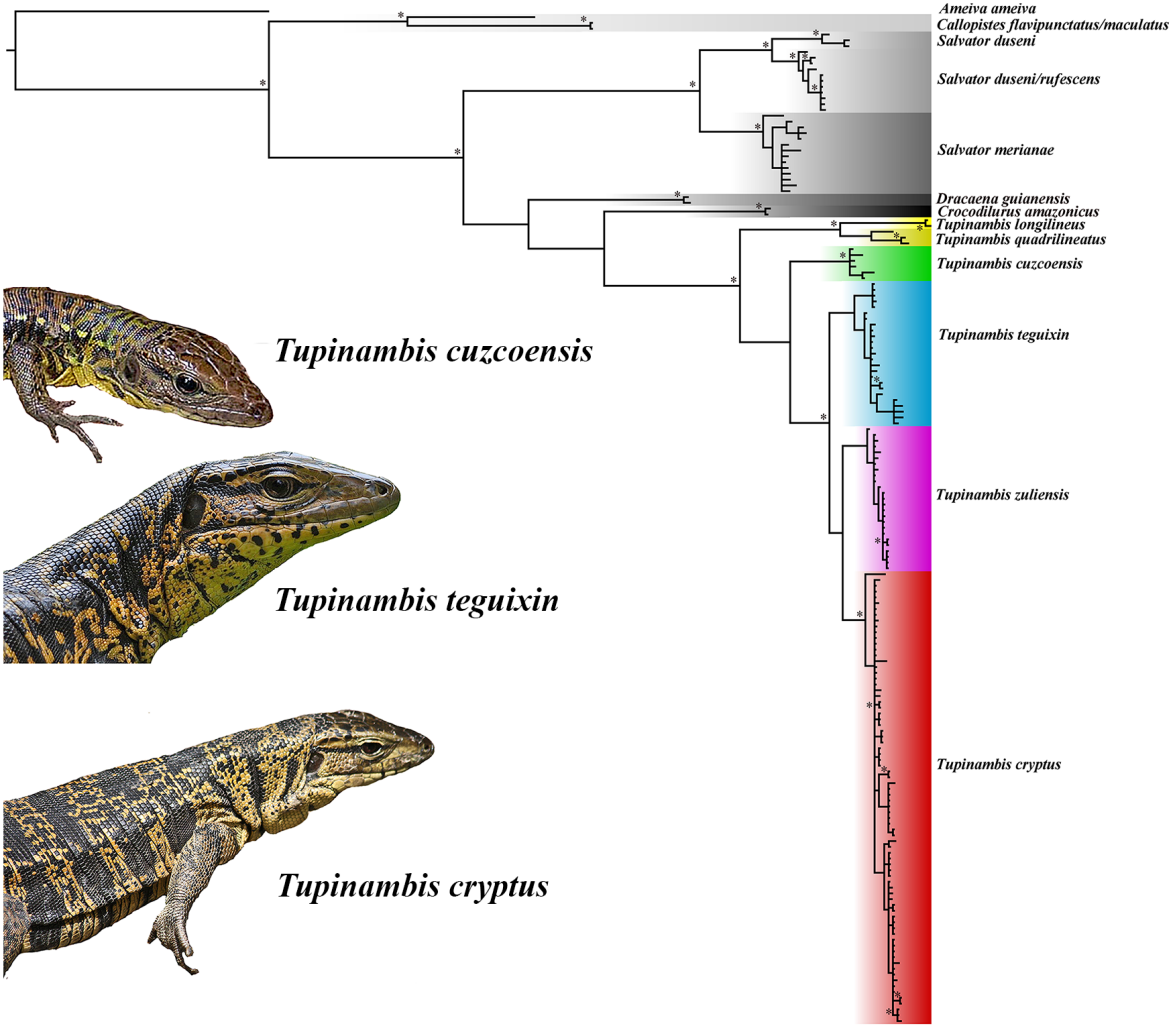
Bayesian majority rule consensus tree clade color coding follows Fig 7. Species illustrated: Top- *Tupinambis cuzcoensis* sp. n., clade 1. Photo credit Mike Pingleton. Second from top *T*. *teguixin*, clade 2. Photo credit Armida Madngisa. Bottom photo *T*. *cryptus* (clade 4). Photo credit JCM. Nodes with Bayesian posterior probabilities ≥ 95 are represented by asterisks (*).

Each of the clades (Fig 8) is moderately to strongly supported (Pp = 89–96). The Maracaibo and eastern Amazon clades are moderately supported (Pp = 83) as sister lineages, and the Cerrado lineage is the sister group of this clade. The western Amazon/Andes clade is the earliest-diverging lineage. Each clade appears to correspond to a species-level taxon. Note that we have not performed an explicit species-delimitation analysis, but these lineages have already been identified as distinct, putatively species-level taxa by previous authors, and are clearly diagnosable morphologically (see below), while being relatively genetically and morphologically homogenous within each lineage. Their status as "cryptic" species is more a reflection of a lack of historical attention to their subtle morphological distinctiveness, resulting in a taxonomic burden of heritage [43].

### Morphological Diversity and Nomenclature

**Fig 9:**
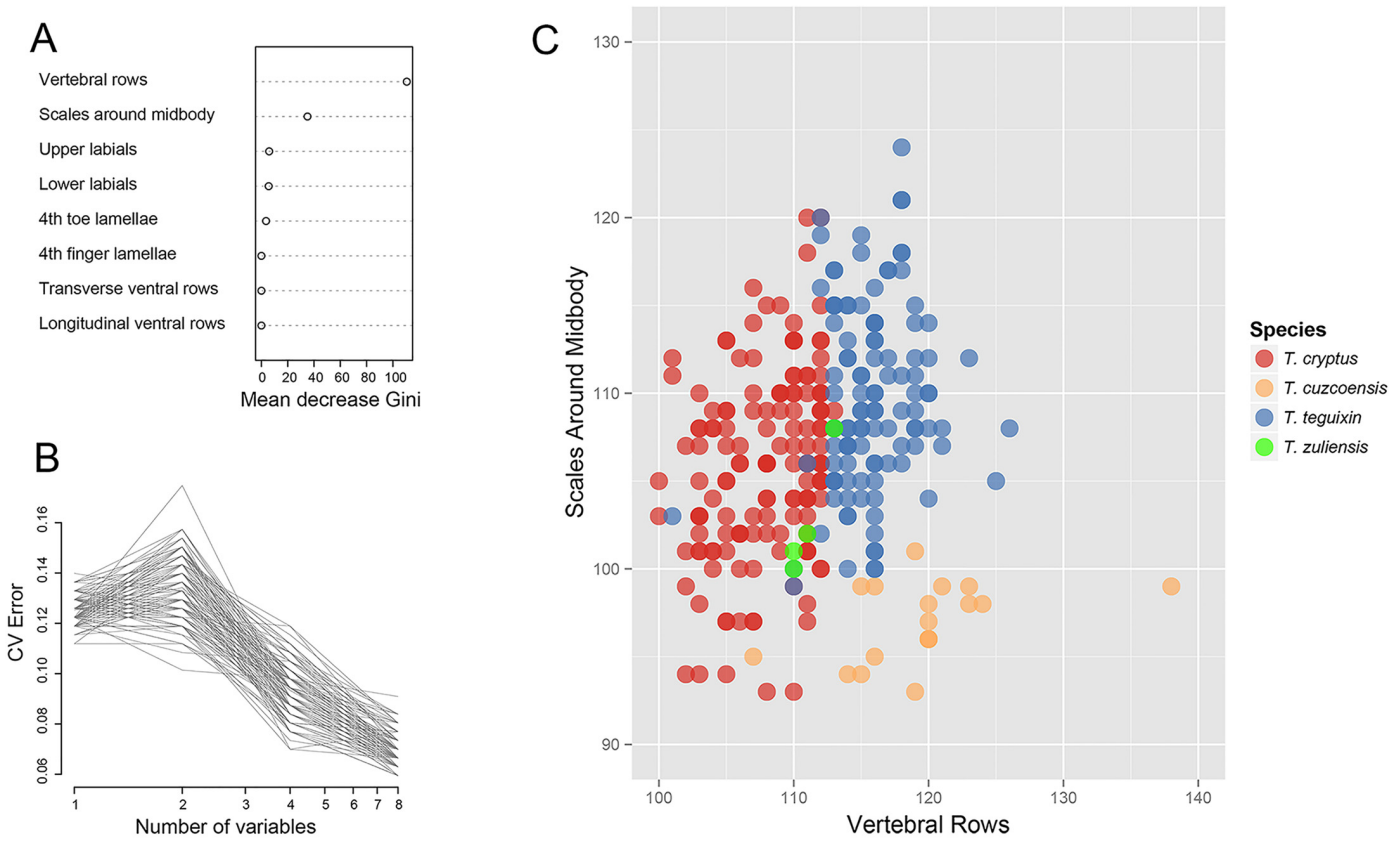
The GRRF results. (A) Importance of meristic counts in predicting individual assignments to four species of *Tupinambis* lizards based on mean decrease in Gini accuracy as revealed by 100 replicates of 10-fold cross-validation of Guided Regularized Random Forests (GRRF). The higher the mean decrease in Gini accuracy, the higher the predictor importance. (B) Prediction error of GRRF models based on reducing number of predictors ranked by importance, as revealed by 100 replicates of 10-fold cross-validation. (C) Variation in vertebral rows and scales around midbody, the two best predictors of differences among four species of *Tupinambis* lizards.

The GRRF analyses indicated that prediction accuracy ranged from 13%, when using the single most important predictor, to 7%, when using all predictors (Fig 9a). Vertebral rows and scales around midbody were the best predictors of the four species, with a prediction accuracy around 87% based on 100 replicates of 10-fold cross-validation (Fig 9b). With the exception of *Tupinambis zuliensis*, which was represented by only four individuals, these two variables permit a fairly good separation of the three other species (Fig 9c).

The results of the cluster analysis [S2A Fig] and PCA [S2B Fig] are in Based on the genetic and morphological analyses describe above, we split the species currently recognized as *Tupinambis teguixin* into four morphologically distinct species, three of which are new. Considering the morphological data collected for this study, it is clear why these lizards have been confused for more than two centuries. Differences are subtle, the coloration and pattern are variable, complex and have an ontogenetic component. Table 1 summarizes the morphology for the four species of the *Tupinambis teguixin* group discussed here, and Table 2 compares the known species in the genus *Tupinambis*.

**Table 1:** Comparison of the four species of the *Tupinambis teguixin* group. IP = interparietal, R = range, SD = standard deviation, X = mean. * = usually three, ** usually two.

	*T*. *cryptus* (n = 119)	*T*. *cuzcoensis* (n = 24)	*T*. *teguixin* (n = 183)	*T*. *zuliensis* (n = 4)
distribution	Guyana, Trinidad, Venezuela	western Amazon Basin	Guianas, Brazil	Maracaibo Basin, Venezuela
R = vertebral rows	100–113	107–124	101–138	110–113
vertebral rows	X = 105.89	X = 117.9	X = 113.70	X = 111
SD+	3.365	2.88	4.32	1.22
R =	94–120	94–110	94–124	100–108
scales around body	X = 104.95	X = 96.2	X = 108.93	X = 102.75
SD	5.21	8.75	5.00	3.11
sample size for traits below	(n = 43)	(n = 7)	(n = 10)	(n = 4)
longest supraocular	1	2	1	2
supraocular largest area	2	2	2	2
ciliaries at last supraocular	3	2 or 3	2	2 or 3
ventrals l/t	20-27/28-37	20-25/28-34	20-28/29-36	22/28-29
total pores	10–26	7–14	19–26	21–23
X	15.95	11	14.67	13
SD	2.94	2.36	2.61	1.22
supratemporals	2–3*	3–4	2–3**	2–3
occipitals at IP	1	1	3	2–5
supraoculars	5	5 or 6	5–7	5
markings on hind legs	vermiculationsnot round spots	uniform or with indistinct spots	distinct rounded spots, vermiculations may be present	distinct round spots
dorsal pattern adults	transverse bands fade w/age	dorsolateral spots indistinct transverse bands	males indistinct transverse bands almost uniform, females with more defined bands	almost uniform dorsum with lateral spots
longest ciliary	first	second	first	first
complete interangular fold	no (except Amazonas population)	no	no	no

**Table 2:** Comparison of the seven species in the genus *Tupinambis*. Data for *longilineus*, *palustris*, *quadrilineatus* were taken from the literature and on-line photographs.

	*T*. *cuzcoensis*	*T*. *cryptus*	*T*. *longilineus*	*T*. *palustris*	*T*. *quadrilineatus*	*T*. *teguixin*	*T zuliensis*
largest supraocular	2	2	2	2	2	2	2
longest supraocular	2	1	2	1	1	1	2
corner of orbit over upper labial	3	4	4	3	4	3	4
lamellae 4th finger	14–18	15–18	10–13	16–18	12–17	14–16	15–16
supratemporals	2–4	2	2	3–4	3	3	2
occipitals at IP	1	1	3	3	3	3	2–5
dorsals	107–124	100–113	110–121	111–122	113–138	101–126	110–113
SAB	92–101	94–113	90–98	112–119	94–118	94–124	100–108
total pores	10–26	7–14	22	18–26	1–18	19–26	21–23

Here, we provide a taxonomic revision to bring taxonomy into concordance with the molecular and morphological results for the *Tupinambis teguixin* complex. The confusion of names presented in these lizards is discussed and illustrated in S3, S3A and S3B Fig. First, we provide a re-description of:

#### *Tupinambis teguixin* (Linnaeus 1758)

Figs 1a and 1c, 3a and 7,

Diagnosis. (1) Five supraoculars, first is usually the longest, but the second is largest in area (note that in some specimens the first and second supraocular are almost equal in length); (2) last supraocular usually contacts two ciliaries; (3) ventral side of male’s head often uniform black during breeding (4) largest prefemoral scales are imbricate, hexagonal, and longer than tall; (5) three enlarged supratemporal scales form one row; (6) three occipitals contact the interparietal scale; (7) rostral readily visible in dorsal view; (8) indistinct transverse bands, may be mostly black in adult males or with a trace of transverse bands (females); (9) the anterior corner of the orbit is over upper labial three. In the molecular analysis this is clade 2.

Size. The largest *Tupinambis teguixin* measured was a male, 279 mm SVL with a 491 mm tail. The smallest was a neonate 84 mm SVL and a 134 mm tail.

Variation. Supraoculars five, (six or seven are not common), the first is the longest; last supraocular usually in contact with two ciliaries; all specimens had three occipitals contacting the interparietal, except one specimen which had two; and all had an incomplete interangular fold; suboculars usually six, one specimen had seven; upper labials 8–10, third or fourth the longest; lower labials 7–8; sublabials 3–5, usually four; chin shields in four pairs, rarely five pairs; lamellae on fourth finger 14–16; lamella on fourth toe 31–36.

Comparisons. *Tupinambis teguixin* is distinguished from the sympatric *Tupinambis cryptus* sp. n. by two supraciliaries contacting the last supraocular (three in *T*. *cryptus* sp. n); usually three occipitals in contact with the interparietal (usually one in *T*. *cryptus* sp. n). *Tupinambis teguixin* differs from *T*. *cuzcoensis* sp. n. in having the first supraocular the longest (the second is the longest in *cuzcoensis*); first pair of chinshields are distinctly longer than the postmental (in *T*. *cuzcoensis* sp. n. the first pair of chinshields are about as long or shorter than the postmental). *Tupinambis teguixin* differs from *Tupinambis zuliensis* sp. n. by having the first supraocular the longest (the second is longest in *T*. *zuliensis*).

Distribution. *Tupinambis teguixin* appears to be widespread in the Amazon and present on the Guiana Shield, ranging from the Caribbean Coast of the Guianas to Roraima and Para, Brazil southward to Mato Grosso and Goias and into western Amazonas, Brazil.

Natural History. Because this species has been long confused with other species of the *T*. *teguixin* group comments on its natural history are difficult to make because they are deeply entangled in the literature with the other cryptic species in the group as well as members of the genus *Salvator*. Considering that *Tupinambis teguixin* and *T*. *cryptus* sp. n. have been collected within 3 km of each other further investigation of these taxa would be of ecological interest.

Etymology. We propose the English name Common Golden Tegu for this species.

#### *Tupinambis cryptus* sp. n

Figs 1d and 1f and 3b and 3c.

Zoo Bank urn:lsid:zoobank.org:act:F107E142-2A05- 4D93-A3B3-2842583EAA80

Diagnosis. (1) Five supraoculars (rarely four or six), first supraocular is the longest, second largest in area; (2) last supraocular contacts three ciliaries (rarely two); (3) ventral side of head usually with heavy mottling and black spots; (4) largest pre-femoral scales are imbricate, hexagonal, and longer than tall; (5) two enlarged supratemporal scales, a second row of enlarged but smaller scales ventral to the two enlarged scales; (6) one occipital usually contacts the interparietal; (7) rostral is readily visible in dorsal view; (8) adult dorsum often has transverse bands that fade with age but are still distinct, does not hold true for the Trinidad and Tobago populations which usually retain well defined bands into adulthood; (9) the anterior corner of the orbit is usually over upper labial four or the seam of upper labials three and four. This species corresponds to clade 4 in the molecular analysis.

Holotype AMNH 140937, male. Size SVL 323 mm, tail broken. Collected 5 March 1994 by Charles J. Cole and Carol R. Townsend at the Dubulay Ranch on the Berbice River, 200 ft asl, 5.681944–57.533333, Guyana.

Description of holotype. Rostral visible from above, posterior border anterior to posterior border of mental; nasals make medial contact behind rostral, completely divided; nostril valvular, ventral border at first and second labial; frontonasal hexagonal, greater than prefrontal length; prefrontals paired, contact first supraciliary and loreal; frontal octagonal, contacts first two supraoculars; frontoparietals paired, pentagonal, contact three supraoculars; interparietal hexagonal, shorter than parietals, contacts three occipitals; parietals partially fragmented, contact two supraoculars, and each in contact with three to four occipitals; 10 occipitals contact parietals; two medial scales on neck at the occipital sulcus are square and large; supraoculars five, first longest, fifth in contact with two ciliaries; ciliaries 9/9, first and ninth equal in length and longest; loreal pentagonal, upper edge longer than ventral edge, in contact with upper labials 2–3; suboculars 6/7, first longest, first five form ridge, in contact with upper labials 3–7; lower eyelid disk with palpebral with four enlarged segments; upper labials nine plate-like scales, fourth longest, anterior edge of orbit over fourth; temporal scales in about eight rows (front to back) smallest ones in front upper half of temporal region, bottom three rows convex polygons; supratemporals first row of two enlarged plates, bordered by a second row of smaller plate-like scales; mental rounded, does not extend passed first pair of lower labials; postmental heptagonal in contact with first two upper labials, with a tapered posterior edge with medial process, anterior edge fragmented; chinshields first pair in contact, five pair, pairs 2–4 separated from lower labials by sublabials; lower labials 8/7 visible, third pair the longest, pairs 2–3 in contact with first pair of chin shields; sublabials five, extend to the third lower labial; interangular fold incomplete, intertympanic sulcus complete; scales on throat: antegulars elongated ovals, juxtaposed, rows disorganized; gulars rounded squares and juxtaposed; mesoptychials hexagonal and juxtaposed; dorsal scales on neck convex, oval, broader than long; dorsal scales on mid-trunk convex, ovate hexagons, longer than broad; vertebral rows 103; transverse ventral rows 31; longitudinal ventral rows 24; the cloacal plate has seven rows of scales from the level of the femoral pores to the free edge of the plate, three of these rows are plate-like scales; scales around mid-body 104; pre-cloacal pores number eight in total, and femoral pores number 6/7; there is a gap of granular scales between the pre-cloacal and femoral pores; tail scales on the proximal dorsal surface are slightly imbricate, convex, smooth, and rectangular; tail scales on proximal ventral surface, are keeled, quadrangular, slightly imbricate, notched posteriorly; anterior surface of forelimbs, upper arm and eight rows on enlarged ovate (with tapered tips) scales; forearm has five rows of square to hexagonal, slightly imbricate plate-like scales; anterior surface of hind limbs: on the femur there are about seven rows of enlarged, slightly imbricate, rectangular scales which transitions into rows of small oval granules on the upper femur; 15 subdigital lamellae fourth finger; 32 subdigital lamellae fourth toe.

Color in alcohol. Crown has mottled plates which are mostly dark with light areas; face is brown-black with light spots on each scale; chin is olive green; throat is mostly olive green with some yellow; neck red brown with gray vermiculated pattern; trunk is a mosaic of light and dark indistinct bands; dorsal surface of legs vermiculated with red brown and yellow-gray; ventral surface is yellow with some black intruding laterally, and black along the seams of the ventral plates; tail is uniform red brown above and laterally, yellow ventrally, distally striped with wide black and slightly narrower yellow bands.

Variation. Temporal scales mostly oval; posterior border of frontal extends posterior to the border of the second and third supraocular in Trinidad specimens examined, but not in Tobago specimens; the frontal is about equal to, or slightly shorter than frontoparietals (frontal is longer than frontoparietals in other species); 8–10 ciliaries, two in contact with last supraocular; 8–9 rows of temporal scales in Trinidad specimens 11 rows in Tobago specimens; 8–10 upper labials, last one or two upper labials are usually hexagonal in Trinidad specimens, in Tobago specimens the last several upper labials are pentagonal), all others rectangular; 7–8 lower labials; lamellae on fourth finger 15–18; lamella on fourth toe 30–38; first pair of chin shields in medial contact, the other three pairs are separated by multiple scales; 3–7 occipitals in contact with parietals in Trinidad population; 11 occipitals in contact with parietals in the Tobago population; 8–14 preanal pores, 12–23 femoral pores per side, 23–36 total pores. The cloacal plate has seven or eight rows of scales from the level of the femoral pores to the free edge of the plate, five of these rows are plate-like scales.

In alcohol the head is olive green with some dark spotting on the crown scales. The lower jaw is yellow and heavily mottled with black pigment. The dorsum of the neck and body is mostly uniform dark brown to black with about 12 rows of indistinct spots. The ventral surface is mostly yellow with dark pigment intruding from the side along the seams of the ventrals, and some patches of dark pigment scattered. This same coloration occurs on the underside of the legs. Proximally, the tail is a solid dark brown-black above with very narrow yellow rings. Juvenile coloration (based on UWIMZ 2012.27.42) is black with markings. Light spot on frontal, and supraoculars outlined in white, stripe on seam of frontoparietals; face mostly gray, with black postocular stripe extending over ear, anterior upper labials gray, posterior upper labials outlined in black; lower labials white with black seams; chin white; dorsum black with 11 irregular, interrupted white cross bands; venter white with scattered black checks; forelimbs banded with alternating black and white bands; hind limbs have irregular elongated yellow markings on dorsal surfaces with only a trace of black stripes on ventral surface; tail with alternating black and white bands, and a white/yellow tail tip. Coloration of juveniles and adults in life can be seen in Fig 1.

There is considerable pattern variation in *Tupinambis cryptus*. Mainland Venezuelan specimens tend to have bands that are indistinct and short longitudinal stripes that Beebe [2] termed “dashes.” A few are almost uniform in coloration. None of the adults from Trinidad and Tobago have this pattern, although we have seen a few individuals in the field with indistinct bands.

Size. The largest *Tupinambis cryptus* sp. n. measured was a male 391 mm SVL with a 530 mm tail. The smallest was a neonate that was 85 mm SVL and a 42 mm tail. Fourteen males SVL 216–391 mm, x = 281.71 mm, SD = 41.11; seven had undamaged tails 463–635, X = 448.56 mm, SD = 60.62. Eleven female SVLs 183–284 mm, X = 231.0 mm, SD = 39.73; only three had unbroken tails 384–605, X = 481.0 mm, SD = 79.87. Smallest individual measured 85 mm SVL.

Comparisons. *Tupinambis cryptus* sp. n. can be distinguished from the sometimes sympatric and syntopic *T*. *teguixin* by its lower number of vertebral rows (average 106 vs 116); three supratemporals (*T*. *teguixin* usually has two); one occipital contacting the interparietal (*T*. *teguixin* usually has three); three ciliaries contacting the last supraocular (*T*. *teguixin* has two); the dorsal surface of the hind legs is uniform in older adults with younger animals having irregular vermiculations (*T*. *teguixin* has light colored round spots—but may also show reticulations); This species can be distinguished from *T*. *cuzcoensis* sp. n., by its lower vertebral scale row count (means 104 vs 119 in *T*. *cuzcoensis*); its longest supraocular is the first (in *T*. *cuzcoensis* sp. n. the second is longest); the species has a higher average number of scale rows around mid-body (106) compared to 98 in *T*. *cuzcoensis*; and the first pair of chin shields are longer than the postmental (*T*. *cuzcoensis* sp n has the first pair of chin shields shorter or about equal to the postmental in length). It can be distinguished from *T*. *zuliensis* sp. n. by having the first supraocular longer than the second.

Distribution. *Tupinambis cryptus* is known from Trinidad and Tobago, Venezuela, the Guianas, and as far south as the confluence of the Rio Negro and Rio Branco in Brazil, but its range may be more extensive. It ranges as far west as Falcon, Venezuela, and appears to range into the Andes in the vicinity of Bucaramango, Colombia.

Natural History. The co-occurrence of this species with *T*. *teguixin* on the Guiana Shield suggests the previous natural history accounts [2] are likely a mixture of these two species. However, *T*. *teguixin* is unknown from Trinidad and Tobago and natural history descriptions from the islands’ populations can be attributed to *T*. *cryptus* alone. Our observations of this lizard suggest they use secondary forest, savannas, and human modified habitats. We have not observed them in primary forests proper, but at the forest edge. It may avoid dense forest because of the reduced number of basking sites. Like other species of *Tupinambis*, *T*. *cryptus* is a dietary generalist. We have observed this lizard investigating caiman nests, foraging along streams on the floor of secondary forests and in mangroves. Usually their tongue is flicking and they are probing the leaf litter with their head. *Tupinambis cryptus* is most readily observed foraging under the bird feeders at the Asa Wright Nature Center (Trinidad) were they scavenge pieces of fruit.

The ecology of the Trinidad population was examined by Everard and Boos [44]. They trapped *Tupinambis cryptus* sp n. at six different sites while studying the mongoose over a six year period. Traps were baited with chicken remains. At the Waller Field study site 56 *T*. *cryptus* were trapped during a 23 week period. In a mark and release study involving 40 animals they had ten recaptures; time between capture and recapture ranged from 1–86 days. The animals moved between 0.0–1.2 km (X = 404 m) and they estimated 79.9 (r = 116.3–43.5) lizards inhabited the 104 ha study site. They report the species feeding on leatherback turtle eggs and ground nesting birds (including nestlings and eggs). Trinidadian folklore [44] states that the young hatch during thunder storms, suggesting Beebe’s [2] observations of females depositing eggs in termite nests is correct for *T*. *cryptus* sp n. Females excavate a chamber in a termite nest (often in arboreal situations), deposit their eggs, and the termites re-seal the nest chamber. The eggs hatch and the hatchlings escape when the termite nest softens during heavy rains.

Etymology. Named *cryptus* for it similarity to *Tupinambis teguixin*. On Trinidad it is commonly known as the matte, on Tobago it is called the salempenta. We propose the Cryptic Golden Tegu as the common English name for this species.

#### *Tupinambis cuzcoensis* sp. n

Figs 1e and 10

**Fig 10:**
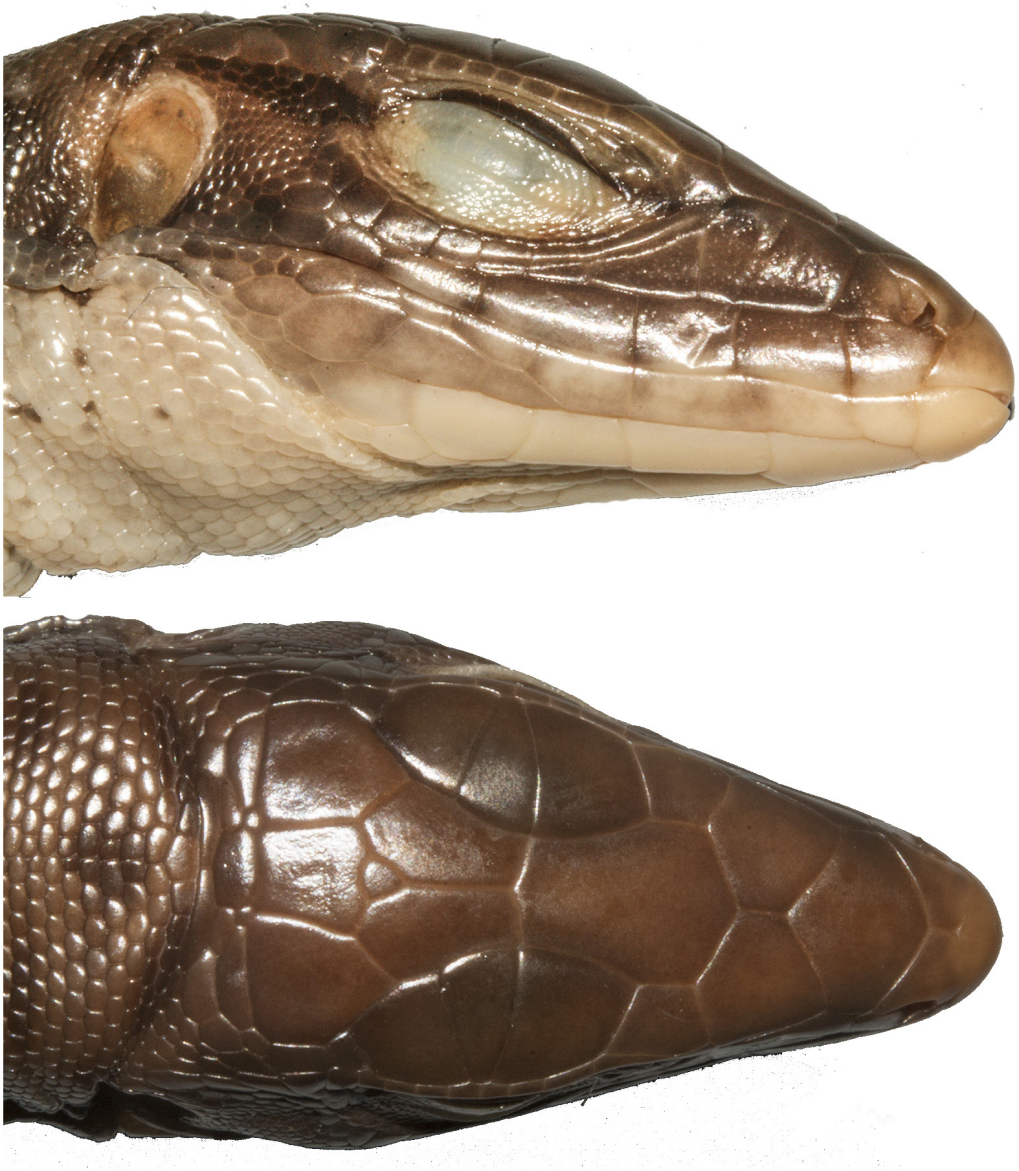
*Tupinambis cuzcoensis* sp. n. FMNH 168330 from Madre Dio, Peru. JCM.

Zoo Bank urn:lsid:zoobank.org:act:9EB27D24-6738-454F-AB62-1360AF1D2C80

Diagnosis. (1) Five or six supraoculars, the second is the longest and the largest in area, (2) last supraocular contacts one or two ciliaries and is exceptionally small; (3) the ventral side of the head is usually uniform white, yellow or olive green to gray; (4) largest prefemorals slightly imbricate, tend to be taller than long, and are hexagonal; (5) two to four enlarged supratemporal scales are bordered by two or three rows ventral rows of smaller scales; (6) three to five occipitals contact the interparietal; (7) rostral visible from above; (8) dorsum has well defined dorsolateral and dorsoventral rows of spots (white in preserved specimens, yellow in life) that may fuse to form a partial stripe; (9) the anterior corner of the orbit is usually over upper labial three or the seam of upper labials three and four. This species corresponds to clade 1 in the molecular analysis.

Holotype FMNH 168228, a male collected by L.E. Pena at Quincemil, Cusco, Peru (~ -13.250–70.735) at 780 m. in August, 1962.

Description of Holotype. Size SVL 247 mm, tail damaged. Posterior border of rostral anterior to posterior border of mental; nasals make medial contact behind rostral, completely divided; nostril valvular, ventral border at first and second labial; frontonasal hexagonal, longer than prefrontal length; prefrontals paired, hexagonal, contact first supraciliary and loreal; frontal octagonal, contacts first three supraoculars; frontoparietals paired, slightly fragmented, pentagonal, contact two supraoculars; interparietal pentagonal, shorter than parietals, contacts two occipitals; parietals contact three supraoculars, and each in contact with 3/2 occipitals; five occipitals contact parietals; occipital sulcus with three polygonal, medial scales only slightly larger than surrounding scales; supraoculars six, second slightly longer than first, fifth in contact with two ciliaries, sixth in contact with one ciliary; ciliaries 7/8, fifth and first longest; loreal pentagonal and fragmented, upper edge longer than ventral edge, in contact with upper labials 2–3; suboculars five, first longest, first four form ridge, in contact with upper labials 3–5; lower eyelid disk with palpebral with three enlarged segments; seven plate–like supralabials scales, fourth longest, anterior edge of orbit over fourth; temporal scales in about 10 rows (front to back) smallest ones fill most of temporal region; supratemporals in two rows, two enlarged plates in first row, second row contains six smaller plate–like scales; mental rounded, does not extend past border of first pair of lower labials; postmental heptagonal in contact with first two lower labials, with a tapered posterior edge and shallow medial process; chinshields in four pairs first pair in contact, third pair partially separated from labials, fourth pair completely separated from labials by sublabials; lower labials 6/6, third longest, pairs 2–3 in contact with first pair of chin shields; sublabials 3/3, extend to the third lower labial; dorsal scales on neck slightly convex, oval, broader than long; dorsal scales on mid–trunk convex, ovate; vertebral rows 120; transverse ventral rows 29; longitudinal ventral rows 22; scales around mid–body 96; pre–cloacal plate formed by six rows of plate–like scales to the level of the precloacal pores; pre–cloacal pores number eight in total, and femoral pores number 5/7; there is a gap of granular scales between the pre–cloacal and femoral pores; tail scales on proximal dorsal surface are juxtaposed, slightly convex, smooth and rectangular; tail scales on proximal ventral surface are smooth, rectangular and imbricate; anterior surface of forelimbs and upper arm covered with six rows of enlarged triangular to ovate scales with tapered tips; forearm has 3–4 four rows of asymmetrical square to pentagonal, slightly imbricate plate–like scales; on anterior femur 7–8 rows of enlarged, slightly imbricate, mostly square to slightly pentagonal scales which abruptly transitions into rows of small oval granules on the upper femur; 14 subdigital lamellae fourth finger; 34 subdigital lamellae fourth toe. Interangular fold incomplete, scales make smooth transition; intertypanic sulcus complete; scales on throat: antegulars polygonal, slightly imbricate, rows somewhat disorganized; gulars polygonal to oval and imbricate; mesoptychials asymmetrical polygons with two enlarged rows of scales.

Color in alcohol. Crown dark brown with darker spots on some of the scales; face uniform dark brown; chin is uniform gray black; no spots on antegulars and gulars; throat is gray with some yellow; neck dark brown with dark 3–4 darker bands; trunk mostly uniform with 11 or 12 light spots in a dorsolateral row on each side; dorsal surface of legs uniform brown and gray; posterior thigh with some mottling; ventral surface is yellow with some black intruding laterally, and scattered black spots on the ventral plates; tail is mostly uniform anteriorly, banded distally, but last half missing.

Variation. Lamellae on fourth finger 14–18; lamellae on fourth toe 29–39; total pores 9–21; occipitals at parietal usually one (one specimen has two); upper labials 8–9 (rarely 7), longest usually fourth (rarely the third); lower labials 6–7; chin shields four pairs in all specimens examined; rows of antegulars 9–12; loreal usually longer than frontonasal.

Comparisons. *Tupinambis cuzcoensis* is the only species discussed here that has the first pair of chin shields equal to or shorter than the postmental. All other species have the first pair of chin shields longer than the postmental. It is also distinctive in having the lowest average number of scales around the mid-body (92.6) and the highest average number of vertebral rows (119). The dorsal pattern is distinctive and consists of transverse bands with a row of dorsolateral spots on each side. The second supraocular is the longest and the largest in area, in the other three species the first supraocular is the longest and the second is the largest in area. The ventral side of the head is uniform in pigmentation.

Distribution. *Tupinambis cuzcoensis* appears to be relatively widespread in the foothills of the Andes and the western Amazon. In Peru it is known from Quincemil (the type locality), Cuzco at about 750 m and goes to at least 827 m (FMNH 81378 is from Villa Carmen, Peru). In Ecuador it occurs in the vicinity of Canelos, near the Rio Bobanasa at about 200 m and, at Zancudo, Napo, Ecuador at about 600 m. It ranges westward into the Amazon Basin as far as Humaita, Amazonas, Brazil and Cuiaba, Mato Grosso, Brazil.

Natural History. Comments on this lizard’s habits can be found in Duellman [45]. He observed it the afternoon along non-forested river banks, and sunning 0.3 m off the ground. He found six arthropods in one stomach (cricket, spiders, beetles, an ant and an orthopteran). He also reports a clutch size of five from a 274 mm SVL female (cited in a paper not seen by us).

#### *Tupinambis zuliensis* sp. n

Fig 11

**Fig 11:**
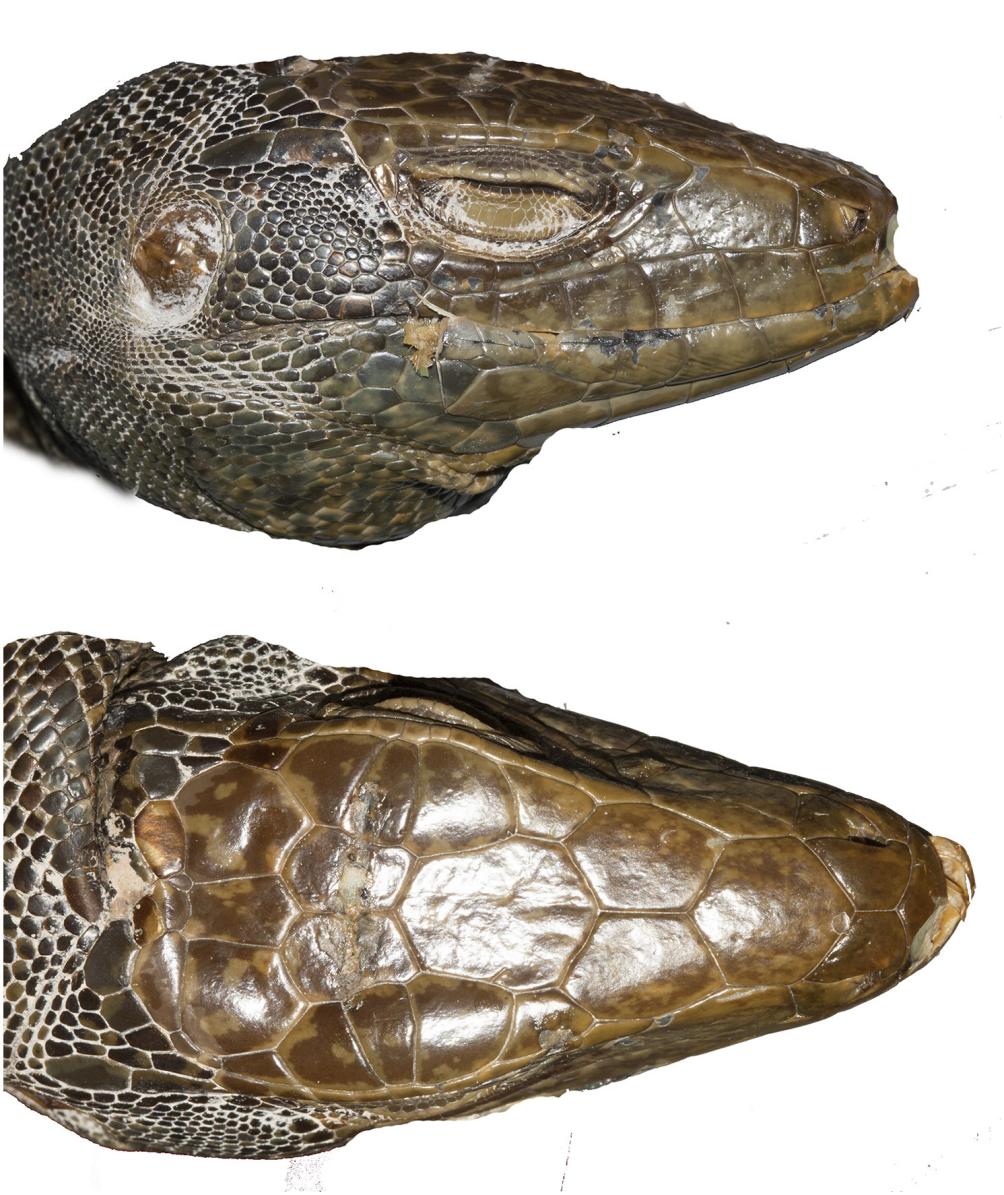
*Tupinambis zuliensis* sp n. FMNH 2599d. The specimen is from Enconstrados, Zulia, Venezuela. JCM.

ZooBank urn:lsid:zoobank.org:act:80EFB0F4-6436-47F0-B4A9-D115FBFF10EC

Holotype FMNH 2599d, a 273 mm SVL male with a 537 mm tail from Encontrados, Zulia, Venezuela (~9.057–72.233). Collected by Ned Dearborn.

Diagnosis. A *Tupinambis* with (1) five supraoculars, the second is the longest and the largest in area; (2) last supraocular contacts two ciliaries; (3) ventral side of head with mottling and black spots; (4) largest prefemoral scales are taller than long, juxtaposed to slightly imbricate, and quadrangular to slightly hexagonal; (5) supratemporal scales two or three in a single row; (6) three or more occipitals at interparietal; (7) rostral barely visible from dorsal view; (8) dorsum with indistinct transverse bands with longitudinal rows of white to yellow spots; (9) the anterior corner of the orbit is usually over upper labial four. This species correspond to clade three.

Description of holotype. Nasals in medial contact behind rostral; completely divided; nostril valvular, ventral border at first and second labial; frontonasal hexagonal, length less than prefrontal; prefrontals paired, contact first supraciliary and loreal; frontal octagonal, contacts first two supraoculars; frontoparietals paired, pentagonal, contact three supraoculars; interparietal hexagonal, shorter than parietals, contacts three occipitals; parietals, contact two supraoculars, and each in contact with two or three occipitals; 14 occipitals, four contact parietals; one medial scale on neck at the occipital sulcus is polygonal and large; supraoculars five, second longest, fifth in contact with two ciliaries; ciliaries 10/10, first longest; loreal pentagonal, upper edge longer than ventral edge, in contact with upper labials 2–3; suboculars 6/6, first longest, first five form ridge, in contact with upper labials 3–7; lower eyelid disk with palpebral with three enlarged segments; upper labials nine (first six plate-like), 3-4-5 longest and equal in length, anterior edge of orbit over third; temporal scales in about eight vertical rows (front to back) smallest scales in anterior upper three-fourths of temporal region, bottom two rows convex polygons; supratemporals two enlarged plates, bordered by a second row of much smaller plate-like scales; mental rounded, does not extend passed first pair of lower labials; postmental heptagonal in contact with first two lower labials, no medial process; four pair of chin shields, first in contact, second to fourth pairs separated from labials by sublabials, separated from each other by antegulars, poorly developed outer antegular row, only slightly enlarged scales; lower labials 7/6 visible, third the longest, second and thirs pairs contact first pair of chin shields; sublabials five, extend to the third lower labial; interangular fold incomplete, intertympanic sulcus complete; scales on throat: antegulars elongated ovals, juxtaposed; gulars ovate and juxtaposed; mesoptychials hexagonal and juxtaposed; dorsal scales on neck convex, oval, broader than long; dorsal scales on mid-trunk convex, ovate hexagons, longer than broad; vertebral rows 111; transverse ventral rows 28; longitudinal ventral rows 22; the cloacal plate has eight rows of plate-like scales from the level of the femoral pores to the free edge of the plate, three of these rows are large; scales around mid-body 102; pre-cloacal pores number nine in total, and femoral pores number 7/7; there is a gap of granular scales between the pre-cloacal and femoral pores; tail scales on the proximal dorsal surface are slightly imbricate, convex, heavily keeled, and rectangular; tail scales on proximal ventral surface, are smooth, quadrangular and slightly imbricate, posterior edge is notched; anterior surface of forelimbs, upper arm has eight rows of enlarged ovate (with tapered tips) scales; forearm has five to seven rows of square to hexagonal, juxtaposed plate-like scales; anterior surface of hind limbs: on the femur there are six to eight rows of enlarged, slightly imbricate, rectangular scales which transitions into rows of small oval granules on the upper femur; 15 subdigital lamellae on fourth finger; 36 subdigital lamellae on fourth toe.

Color in alcohol. Crown has uniform to slightly mottled plates which are mostly dark brown; face is brown-black with mottling on upper labials, loreal and first subocular; chin is olive green; throat is mostly olive green with some yellow and black mottling; trunk is darkly pigmented with six to eight rows of indistinct spots laterally; dorsal surface of legs uniform dark brown with some traces of round white spots; ventral surface is yellow-red with some black intruding laterally, and black often along the seams of the ventral plates; tail is uniform dark brown above and laterally and ventrally; distally banded with indistinct wide black yellow bands.

Variation. Lamellae on fourth finger 15–16; lamellae on fourth toe 36–38; total pores 21–23; occipitals at parietal usually 4–5; upper labials 7–8, the second through fourth are equal in length and the longest; lower labials 5–7; chin shields 4–5 pairs; rows of antegulars 9–10; loreal longer than frontonasal.

Comparisons. *Tupinambis zuliensis* sp. n. can be distinguished from *T*. *teguixin* and *T*. *cryptus* by having the second supraocular the longest and the largest in area (the other two species have the first supraocular the longest and the second is largest in area). It can be distinguished from *T*. *cuzcoensis* by having the post mental longer than the first pair of chin shields (it is shorter than the first pair of chin shields in *T*. *cuzcoensis*).

Etymology. This lizard is named after the Venezuela state it occurs in, Zulia. We suggest the English common name Maracaibo Basin Tegu Lizard for this species.

Natural History. Nothing known.

## Discussion

Here we describe three cryptic species related to *Tupinambis teguixin* on the basis of morphology and genetics.

The *Tupinambis teguixin* group is morphologically conserved, and when combined with our historical reliance on measurable and descriptive characters, has led us to underestimate the diversity in this lizard complex for almost 250 years. Our attempt to resolve cryptic species with molecular and morphological evidence yields two sympatric and undoubtedly syntopic species (*T*. *cryptus* sp. n. and *T*. *teguixin*) that are living alongside each other and cannot be easily distinguished in the field. Each cryptic species represents a monophyletic lineage and three of these species appear to have distributions that are potentially sympatric and syntopic at some locations. Needless to say, we have not been able to rule out the possibility that other cryptic species are present in northeastern South America and, in fact, think it likely based upon morphology that did not agree well with any of the species discussed here. Thus, other species of the *T*. *teguixin* group remain to be defined.

Hatchling and juvenile coloration and pattern remain to be elucidated for each of these taxa. Our initial thought was that *T*. *teguixin* hatchlings have wide dark transverse bands separated by narrow yellow or white bands, and that *T*. *cryptus* sp. n. hatchlings and juveniles have alternating transverse bands that are about equal in length. However, the Tobago population of *T*. *cryptus* does not conform to this pattern, suggesting variation within each of these species is complex and overlapping. Speciation is not always accompanied by recognizable phenotypic change [46].

Evidence [47, 48] suggests that a molecular phylogeny can serve as a sorting mechanism when specimens are examined with the hindsight of this tool. Morphological characters once considered ‘‘individual variation” can be reliable apomorphies for species identification, although these characters are few in number in the *T*. *teguixin* group.

*Tupinambis teguixin* has been given two common names, the golden tegu (*T*. *teguixin* and *T*. *cryptus*) and the black and white tegu. *Salvator merianae* is also mostly black and white [18]. The two species of golden tegus, *T*. *teguixin* and *T*. *cryptus*, both have adult patterns of black, brown and gold/yellow/white, with adult male *T*. *teguixin* tending to be darker than *T*. *cryptus* males based upon our relatively small sample of live specimens.

**Fig 12:**
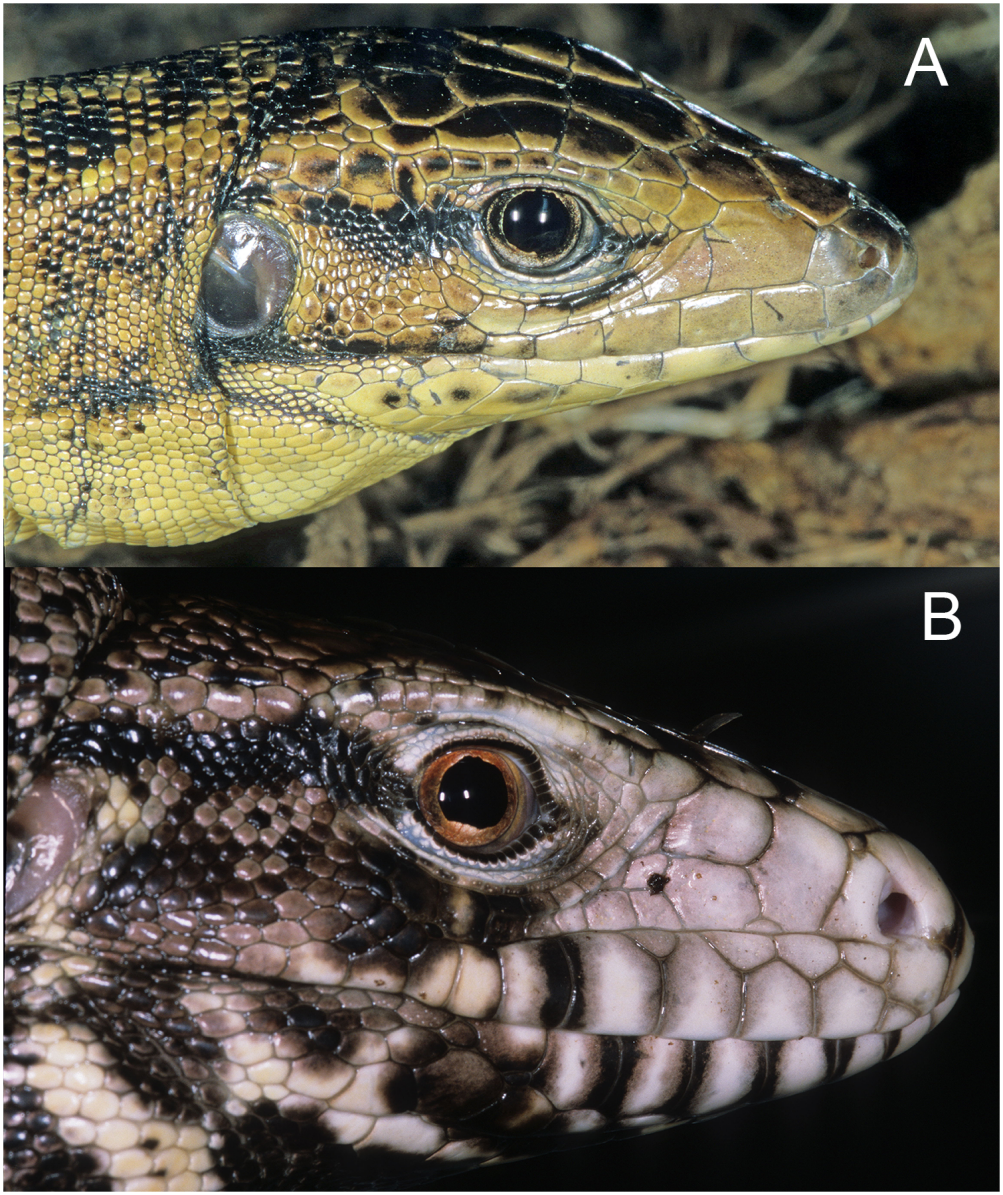
Compares a member of the *Tupinambis teguixin* Group and a *Salvator merianae*. The two species have been long confused in the literature. Both specimens were in the pet trade and are from unknown localities. Diagnostic characters are obvious. A. *Tupinambis teguixin* lacks granular scales separating the supraoculars from the ciliaries, it has a single loreal scale, and the head is slightly compressed (dorsoventrally). B. *Salvator merianae* has granular scales between the supraocualars and the cillaries, a divided loreal, and a deep head. Also note the tall and horizontally divided lower labials. Photographs by JCM.

Unexpectedly, we found a substantial amount of confusion still exists between members of the *Tupinambis teguixin* group and the *Salvator merianae* group. Müller [49] named *T*. *teguixin sebastiani and T*. *t*. *buzioensis* based on island populations in southeast Brazil. Böhme [50] recognized these as belonging to *T*. (= *Salvator*) *merianae*, not *T*. *teguixin*, and reallocated the names appropriately. A recently published paper on exotic reptiles in the Philippine pet trade lists *Tupinambis teguixin*, however the accompanying photograph clearly shows a species of *Salvator* [51]. Fig 12 compares profiles of *Salvator merianae* and *Tupinambis teguixin*. Data for the number of *Tupinambis teguixin* group members involved in the novelty leather trade are apparently unknown. While large numbers of tegus are taken from the wild each year for the leather industry, most of these appear to be *Salvator merianae*, or another member of the *Salvator* clade. Members of the *Tupinambis teguixin* group are likely more often consumed as bush meat or enter the pet trade. The confusion between *T*. *teguixin* group members and *S*. *merianae* is on-going and basal to the confusion.

Anecdotally, an internet search for photographs of *Tupinambis teguixin* produces nearly as many photographs of *Salvator merianae* labeled *T*. *teguixin* as it does *T*. *teguixin* photographs. Websites such as the Encyclopedia of Life, i-Naturalist, and the Reptile Database have photographs of both *S*. *merianae* and the *T*. *teguixin* group labeled *T*. *teguixin*. The confusion of these two tegu lizards has been on-going for centuries and carries into their life history descriptions and ultimately into conservation polices.

**Fig 13:**
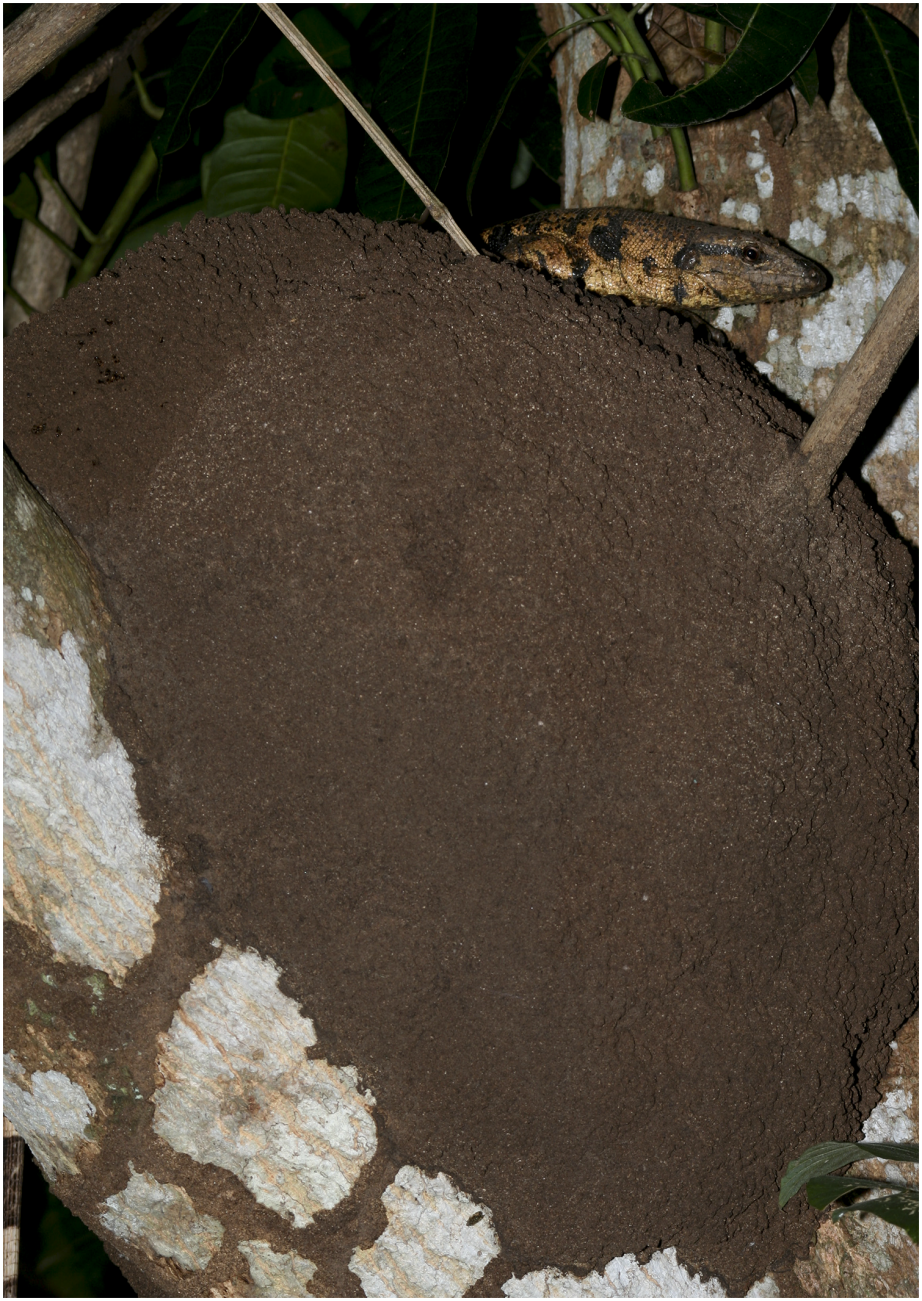
A female *Tupinambis cryptus* investigating an arboreal termite nest as a possible location to deposite her eggs. Photo credit Graham White.

Early descriptions of members of the *Tupinambis teguixin* group depositing small clutches of eggs in termitaria were reported under the name *T*. *nigropunctatus* [52–55] for Brazilian and Guyana specimens. Krieg [55] contrasted *T*. *nigropunctatus* laying eggs in termite nests, to female *T*. *teguixin* laying eggs in the ground and guarding the nest. However, his photograph labeled *T*. *teguixin* is clearly a specimen of *Salvator merianae*. The current literature suggests female *Salvator* are excavating nests in the ground to deposit relatively large clutches of 20–50 eggs [56, 57], while *T*. *teguixin* group members lay smaller clutches of eggs in termitaria. These differences in life history traits likely make *S*. *merianae* much more capable of withstanding exploitation, than members of the *T*. *teguixin* group [58]. Given the absence of data that *Tupinambis teguixin* is present on Trinidad and Tobago and Venezuela it is probable that studies and observations on those populations apply only to *T*. *cryptus* [59–61]. Thus, the reproductive cycle described by Herrera and Robinson [62] applies to *T*. *cryptus* and shows female gonadal mass increasing during the wet season and mating and oviposition occurring in the early to mid-dry season (roughly February to April). The nest is excavated in a dry season termite mound by the female and the egg chamber is then re-sealed by the insects (Fig 13). This raises the question, do all members of the *T*. *teguixin* group use termitaria as oviposition sites, or is it just *T*. *cryptus*? Hagmann [53] contains photographs of what appear to be *T*. *teguixin* from the llha Mexiana, Brazil (about -0.03611–49.602500) that were hatched from a termitaria. Thus, it seems probable that all *T*. *teguixin* group members uses this mode of reproduction and it is a synapomorphy for the clade, if not the genus.

*Tupinambis teguixin* and *T*. *cryptus* eggs incubate until the first heavy rains in June or July. Incubation time is thus on the order of 150–180 days compared to incubation times of about 60 days in *Salvator merianae* which places its nests in the ground [57]. There is some anecdotal evidence that female *T*. *teguixin* group members may use communal nests and that the incubation time of eggs laid in captivity may be as long as 170 days which supports the 150–180 incubation period suspected in nature [10].

Recently, Gols-Ripoll *et al*. [42] examined the genetic structure of Venezuelan *Tupinambis* in different bioregions using nucleotide diversity, haplotype diversity and number of polymorphic sites. They found genetic structuring with all three measures and suggest it is the result of historic biogeographic events, the Mérida Andes orogeny and the shifts in Orinoco River (which accounted for 71.2% of the molecular variance) as barriers. They considered the Zulia population (now *Tupinambis zuliensis*) an evolutionary significant unit. Since we have not found evidence of *T*. *teguixin* in Venezuela, all of their comments likely apply *T*. *cryptus* (and *T*. *zuliensis*). We did find morphological differences between several of the Venezuela *T*. *cryptus* populations which supports the genetic structure found by Gols-Ripoll *et al*. [42]. Specimens from Amazonas tended to be dark, almost melanistic with very little yellow pigmentation, and differed from other *T*. *cryptus* populations in having three occipitals contacting the interparietal and a complete fold at the interangular sulcus. The Orinoco populations tend to have a dorsal pattern of distinct transverse bands interrupted by longitudinal lines. However, both populations share the other traits typical of *T*. *cryptus*.

Similarly, we found differences between the Tobago and Trinidad populations of *T*. *cryptus*. Personnel at the Emperor Valley Zoo in Trinidad told us that specimens from the two islands did not look alike. Preliminary morphological examination of a few specimens suggested they were correct. But in a first comparison of gene sequences between Trinidad and Tobago specimens the animals were only 0.06% different and most of the morphological traits all fall into the range of *T*. *cryptus*.

*Tupinambis cuzcoensis* is the most basal clade and its Andean foothills—western Amazon distribution suggests the Andean uplift may have been involved in its origin. The rise of the Mérida Andes likely isolated *T*. *zuliensis* from the other populations and the eastward shifts of the Orinoco River are likely to have influenced the evolution of the *T*. *cryptus* and *T*. *teguixin*. The absence of *T*. *teguixin* in Venezuela may suggest that the Orinoco or a marine incursion separated the eastern and western populations and allowed speciation with the subsequent eastward dispersal of *T*. *cryptus* to the Guyanas and Brazil.

## Supporting Information

S1 Table Specimen vouchers and Genbank accession numbers. (PDF)

S2 Table Morphological data. (PDF)

S1 Fig Estimated phylogenies using MrBayes3.2.5. (PDF)

S2 Fig Morphological analysis using a cluster analysis (S2A) and PCA (S2B). (PDF)

S3 Fig Elaboration on some nomenclatural issues and illustrated with S3A and S3B Fig. (PDF)
