# Supplementary material for: Cryptic, Sympatric Diversity in Tegu Lizards of the Tupinambis teguixin Group (Squamata, Sauria, Teiidae) and the Description of Three New Species
Source: PLoS One. 2016 Aug 3;11(8):e0158542. doi: 10.1371/journal.pone.0158542 (PMC4972348; doi:10.1371/journal.pone.0158542)
Supplement: S1 Table — (PDF) [file pone.0158542.s001.pdf]

Supporting Information 1 Table. Specimen vouchers and GenBank accessions.

Species	Voucher	12S	16S	ND4	CYTB	Locality	Lat	Long
Tupinambis teguixin	AMS009	KU894506	-	-	-		4.736000	-58.720000
Tupinambis teguixin	BPN3729	KU894509	KU894544	-	-		2.182000	-59.337000
Tupinambis teguixin	CG366	KU894511	KU894555	-	-			
Tupinambis teguixin	CHUNB00476	KU894526	KU894563	KU894572	-	Boa Vista	2.816680	-60.670500
Tupinambis teguixin	CHUNB00485	KU894533	KU894565	KU894575	-	Humaitá	-7.512220	-63.026700
Tupinambis teguixin	CHUNB10757	KU894521	KU894545	KU894573	-	Santa Terezinha	-10.466100	-50.506300
Tupinambis teguixin	CHUNB14017	KU894510	KU894546	-	-	Chapada dos Guimarães	-15.460600	-55.745000
Tupinambis teguixin	CHUNB14846	KU894513	-	-	-	Minaçu	-13.535100	-48.223900
Tupinambis teguixin	CHUNB15207	KU894508	-	-	-			
Tupinambis teguixin	CHUNB36081	KU894522	-	-	-	Monte Alegre	-2.003330	-54.073400
Tupinambis teguixin	CHUNB37555	KU894515	KU894556	-	-	Novo Progresso	-7.034840	-55.419900
Tupinambis teguixin	CHUNB45404	KU894517	KU894558	-	-	Caseara	-9.267410	-48.948700
Tupinambis teguixin	CHUNB47007	KU894527	KU894553	-	-	Alta Floresta	-9.871720	-56.091600
Tupinambis teguixin	CHUNB49926	KU894518	KU894559	-	-	Palmas	-10.163300	-48.351000
Tupinambis teguixin	CHUNB50776	KU894514	KU894554	-	-	Cerejeiras	-13.195000	-60.818400
Tupinambis teguixin	CHUNB52140	KU894519	KU894560	KU894574	-	Carolina	-7.330780	-47.470100
Tupinambis teguixin	CHUNB52479	KU894525	-	-	-	Peixe	-12.029800	-48.537800
Tupinambis teguixin	CHUNB58099	KU894524	KU894561	-	-	Santana do Araguaia	-9.329420	-50.344800
Tupinambis teguixin	CHUNB58270	KU894520	KU894547	-	-	Novo Santo Antônio	-12.294700	-50.969200
Tupinambis teguixin	LH608	-	-	-	-			
Tupinambis teguixin	LH673	KU894529	KU894549	-	-			
Tupinambis teguixin	RHO495	KU894512	KU894550	-	-			
Tupinambis teguixin	UFMT5919	KU894528	KU894552	-	-			
Tupinambis teguixin	UFMT7205	KU894523	KU894551	-	-			
Tupinambis teguixin	UFMT8133	KU894516	KU894548	-	-			
**Murphy**								
Tupinambis teguixin	UWIZM.2012.38.6	KU894532	KU894557	-	-	Lopinot, Trinidad	10.671891	-61.325395
Tupinambis teguixin	UWIMZ 2012.27.42	KU894531	KU894564	-	-	Tobago – Cambleton Bay Trail	11.316000	-60.583000
Tupinambis teguixin	AMNH133345	KU894504	KU894541	-	-	Suriname, Brokopondo	5.662778	-57.890000
Tupinambis teguixin	AMNH140937	KU894530	KU894562	-	-	Guyana- Dubulay Ranch, Berbice River	5.681944	-57.858889
Tupinambis teguixin	AMNH140938	KU894507	KU894543	-	-	Guyana- Warniabo Creek 4 miles from Dubulay Ranch house on Berbice River	5.697276	-57.837631
Tupinambis teguixin	MVZ247605	KU894505	KU894542	-	-	Suriname, Irene Val (Fall), Brownsberg Nature Park	4.943366	-55.170699
**GenBank**								
Tupinambis teguixin	KU205023	AY046422	AY046464	-	-	PERU: Madre de Dios; Cuzco Amazonico, 15 km E Puerto Maldonado	-12.590908	-69.196314
Tupinambis teguixin	LM2421	AY359490	AY359510	-	-	SBH 267102; Peru: Cuzco Amazonico	-12.590908	-69.196314
Tupinambis teguixin	LSUMNS H-12405	-	-	AF151211	AF151183	Brazil: Roraima: Fazenda Nova Esperanca, 44km W BR-174 of BR-210	2.011146	-61.472123
Tupinambis teguixin	LSUMNS H-12431	-	-	AF151212	AF151184	Brazil: Roraima: Fazenda Nova Esperanca, 44km W BR-174 of BR-210	2.011146	-61.472123
Tupinambis teguixin	LSUMNS H-12450	-	-	AF151213	AF151185	Brazil: Roraima: Fazenda Nova Esperanca, 44km W BR-174 of BR-210	2.011146	-61.472123
Tupinambis teguixin	LSUMNS H-12678	-	-	AF151200	AF151186	Ecuador, Cuyabeno	-0.217614	-75.806908
Tupinambis teguixin	LSUMNS H-12703	-	-	AF151201	AF151187	Ecuador, Cuyabeno	-0.217614	-75.806908
Tupinambis teguixin	LSUMNS H-12715	-	-	AF151202	AF151188	Ecuador, Cuyabeno	-0.217614	-75.806908
**Outgroups**								
Salvator duseni	CHUNB12503	KU894496	-	KU894566	-			
Salvator duseni	CHUNB24850	KU894497	KU894534	-	-			
Salvator merianae	CHUNB00486	-	KU894535	-	-			
Salvator merianae	CHUNB00503	KU894498	KU894536	KU894568	-			
Salvator merianae	LH607	KU894499	KU894537	KU894569	-			
Salvator merianae	RHO842	KU894500	-	-	-			
Salvator rufescens	CHUNB08828	KU894501	KU894538	KU894567	-			
Tupinambis quadrilineatus	CHUNB14010	KU894502	KU894539	KU894570	-			
Tupinambis quadrilineatus	CHUNB37470	KU894503	KU894540	KU894571	-			
**GenBank Outgroups**								
Ameiva ameiva	Various	AY046423	AY046465	AF151206	AF151192			
Callopistes flavipunctatus	MHNSMuncat	EF029873	EF029880	-	-			
Callopistes maculatus	MNHNuncat1	EF029874	EF029881	-	-			
Callopistes maculatus	MNHNuncat2	EF029875	EF029882	-	-			
Crocodilurus amazonicus	CHUNB32582	EF029877	EF029884	-	-			
Crocodilurus amazonicus	CHUNB32614	EF029876	EF029883	-	-			
Dracaena guianensis	CHUNB15197	EF029879	EF029886	-	-			
Dracaena guianensis	CHUNB15199	EF029878	EF029885	-	-			
Salvator duseni	USNMField166766	-	-	AF151199	AF151179			
Salvator duseni	USNMField166777	-	-	AF151208	AF151180			
Salvator duseni	USNMField166778	-	-	AF151198	AF151178			
Salvator merianae	KRC060	-	JQ627299	-	-			
Salvator merianae	KRC065	-	JQ627300	-	-			
Salvator merianae	M303	-	-	KF034085	-			
Salvator merianae	M304	-	-	KF034087	-			
Salvator merianae	M306	-	-	KF034084	-			
Salvator merianae	MIC47	-	-	KF034086	-			
Salvator merianae	USNMField166649	-	-	AF151194	AF151174			
Salvator merianae	USNMField166756	-	-	AF151197	AF151177			
Salvator merianae	USNMField166779	-	-	AF151209	AF151181			
Salvator merianae	USNMField166780	-	-	AF151210	AF151182			
Salvator rufescens	R587	-	-	KF034092	-			
Salvator rufescens	R590	-	-	KF034088	-			
Salvator rufescens	R598	-	-	KF034089	-			
Salvator rufescens	R606	-	-	KF034090	-			
Salvator rufescens	R741	-	-	KF034093	-			
Salvator rufescens	R744	-	-	KF034091	-			
Salvator rufescens	USNMField166740	-	-	AF151195	AF151175			
Salvator rufescens	USNMField166743	-	-	AF151196	AF151176			
Tupinambis longilineus	LSUMNSH-14135	-	-	AF151203	AF151189			
Tupinambis longilineus	LSUMNSH-14136	-	-	AF151204	AF151190			
Tupinambis quadrilineatus	LG1132	-	AY217991	KC621501	-			
**Gols**								

Tupinambis teguixin	CVG44	-	-	KM259772	KM259676	Campamento Mirador del Frio, Guri	7.758333	-63.082779
Tupinambis teguixin	CVG45	-	-	KM259773	KM259677	Campamento Mirador del Frio, Guri	7.758333	-63.082779
Tupinambis teguixin	CVG46	-	-	KM259774	KM259678	Campamento Mirador del Frio, Guri	7.758333	-63.082779
Tupinambis teguixin	CVG47	-	-	KM259775	KM259679	Campamento Mirador del Frio, Guri	7.758333	-63.082779
Tupinambis teguixin	CVG48	-	-	KM259776	KM259680	Campamento Mirador del Frio, Guri	7.758333	-63.082779
Tupinambis teguixin	CVG49	-	-	KM259777	KM259681	Campamento Mirador del Frio, Guri	7.758333	-63.082779
Tupinambis teguixin	CVG50	-	-	KM259778	KM259682	Campamento Mirador del Frio, Guri	7.758333	-63.082779
Tupinambis teguixin	CVG51	-	-	KM259779	KM259683	Campamento Mirador del Frio, Guri	7.758333	-63.082779
Tupinambis teguixin	CVG52	-	-	KM259780	KM259684	Campamento Mirador del Frio, Guri	7.758333	-63.082779
Tupinambis teguixin	CVG53	-	-	KM259781	KM259685	Campamento Mirador del Frio, Guri	7.758333	-63.082779
Tupinambis teguixin	CVG54	-	-	KM259782	KM259686	Campamento Mirador del Frio, Guri	7.758333	-63.082779
Tupinambis teguixin	CVG55	-	-	KM259783	KM259687	Campamento Mirador del Frio, Guri	7.758333	-63.082779
Tupinambis teguixin	CVG56	-	-	KM259784	KM259688	Campamento Mirador del Frio, Guri	7.758333	-63.082779
Tupinambis teguixin	CVG57	-	-	KM259785	KM259689	Campamento Mirador del Frio, Guri	7.758333	-63.082779
Tupinambis teguixin	CVG58	-	-	KM259786	KM259690	Campamento Mirador del Frio, Guri	7.758333	-63.082779
Tupinambis teguixin	CVG59	-	-	KM259787	KM259691	Campamento Mirador del Frio, Guri	7.758333	-63.082779
Tupinambis teguixin	CVG60	-	-	KM259788	KM259692	Campamento Mirador del Frio, Guri	7.758333	-63.082779
Tupinambis teguixin	CVG61	-	-	KM259789	KM259693	Campamento Mirador del Frio, Guri	7.758333	-63.082779
Tupinambis teguixin	DE001	-	-	KM259730	KM259634	Boca de Macareo	8.915511	-61.978901
Tupinambis teguixin	DE002	-	-	KM259731	KM259635	Boca de Macareo	8.915511	-61.978901
Tupinambis teguixin	DE003	-	-	KM259732	KM259636	Boca de Macareo	8.915511	-61.978901
Tupinambis teguixin	DE004	-	-	KM259733	KM259637	Boca de Macareo	8.915511	-61.978901
Tupinambis teguixin	DE005	-	-	KM259734	KM259638	Boca de Macareo	8.915511	-61.978901
Tupinambis teguixin	DE006	-	-	KM259735	KM259639	Boca de Macareo	8.915511	-61.978901
Tupinambis teguixin	DE007	-	-	KM259736	KM259640	Boca de Macareo	8.915511	-61.978901
Tupinambis teguixin	DE008	-	-	KM259737	KM259641	Boca de Macareo	8.915511	-61.978901
Tupinambis teguixin	DE010	-	-	KM259738	KM259642	Boca de Macareo	8.915511	-61.978901
Tupinambis teguixin	DE011	-	-	KM259739	KM259643	Boca de Macareo	8.915511	-61.978901
Tupinambis teguixin	DE012	-	-	KM259740	KM259644	Boca de Macareo	8.915511	-61.978901
Tupinambis teguixin	DE013	-	-	KM259741	KM259645	Boca de Macareo	8.915511	-61.978901
Tupinambis teguixin	DE014	-	-	KM259742	KM259646	Boca de Macareo	8.915511	-61.978901
Tupinambis teguixin	DE015	-	-	KM259743	KM259647	Boca de Macareo	8.915511	-61.978901
Tupinambis teguixin	DE016	-	-	KM259744	KM259648	Boca de Macareo	8.915511	-61.978901
Tupinambis teguixin	DE017	-	-	KM259745	KM259649	Boca de Macareo	8.915511	-61.978901
Tupinambis teguixin	DE018	-	-	KM259746	KM259650	Boca de Macareo	8.915511	-61.978901
Tupinambis teguixin	GUA63	-	-	KM259790	KM259694	Guatupo National Park	10.083333	-66.416667
Tupinambis teguixin	GUA64	-	-	KM259791	KM259695	Guatupo National Park	10.083333	-66.416667
Tupinambis teguixin	GUA65	-	-	KM259792	KM259696	Guatupo National Park	10.083333	-66.416667
Tupinambis teguixin	GUA66	-	-	KM259793	KM259697	Guatupo National Park	10.083333	-66.416667
Tupinambis teguixin	GUA67	-	-	KM259794	KM259698	Guatupo National Park	10.083333	-66.416667
Tupinambis teguixin	GUA68	-	-	KM259795	KM259699	Guatupo National Park	10.083333	-66.416667
Tupinambis teguixin	PM019	-	-	KM259747	KM259651	Agropecuaria Puerto Miranda	7.905450	-67.479961
Tupinambis teguixin	PM020	-	-	KM259748	KM259652	Agropecuaria Puerto Miranda	7.905450	-67.479961
Tupinambis teguixin	PM021	-	-	KM259749	KM259653	Agropecuaria Puerto Miranda	7.905450	-67.479961
Tupinambis teguixin	PM022	-	-	KM259750	KM259654	Agropecuaria Puerto Miranda	7.905450	-67.479961
Tupinambis teguixin	PM023	-	-	KM259751	KM259655	Agropecuaria Puerto Miranda	7.905450	-67.479961
Tupinambis teguixin	PM024	-	-	KM259752	KM259656	Agropecuaria Puerto Miranda	7.905450	-67.479961
Tupinambis teguixin	PM025	-	-	KM259753	KM259657	Agropecuaria Puerto Miranda	7.905450	-67.479961
Tupinambis teguixin	PM026	-	-	KM259754	KM259658	Agropecuaria Puerto Miranda	7.905450	-67.479961
Tupinambis teguixin	PM027	-	-	KM259755	KM259659	Agropecuaria Puerto Miranda	7.905450	-67.479961
Tupinambis teguixin	PM028	-	-	KM259756	KM259660	Agropecuaria Puerto Miranda	7.905450	-67.479961
Tupinambis teguixin	PM029	-	-	KM259757	KM259661	Agropecuaria Puerto Miranda	7.905450	-67.479961
Tupinambis teguixin	PM030	-	-	KM259758	KM259662	Agropecuaria Puerto Miranda	7.905450	-67.479961
Tupinambis teguixin	PM031	-	-	KM259759	KM259663	Agropecuaria Puerto Miranda	7.905450	-67.479961
Tupinambis teguixin	PM032	-	-	KM259760	KM259664	Agropecuaria Puerto Miranda	7.905450	-67.479961
Tupinambis teguixin	PM033	-	-	KM259761	KM259665	Agropecuaria Puerto Miranda	7.905450	-67.479961
Tupinambis teguixin	PM034	-	-	KM259762	KM259666	Agropecuaria Puerto Miranda	7.905450	-67.479961
Tupinambis teguixin	PM035	-	-	KM259763	KM259667	Agropecuaria Puerto Miranda	7.905450	-67.479961
Tupinambis teguixin	PM036	-	-	KM259764	KM259668	Agropecuaria Puerto Miranda	7.905450	-67.479961
Tupinambis teguixin	PM037	-	-	KM259765	KM259669	Agropecuaria Puerto Miranda	7.905450	-67.479961
Tupinambis teguixin	PM038	-	-	KM259766	KM259670	Agropecuaria Puerto Miranda	7.905450	-67.479961
Tupinambis teguixin	PM039	-	-	KM259767	KM259671	Agropecuaria Puerto Miranda	7.905450	-67.479961
Tupinambis teguixin	PM040	-	-	KM259768	KM259672	Agropecuaria Puerto Miranda	7.905450	-67.479961
Tupinambis teguixin	PM041	-	-	KM259769	KM259673	Agropecuaria Puerto Miranda	7.905450	-67.479961
Tupinambis teguixin	PM042	-	-	KM259770	KM259674	Agropecuaria Puerto Miranda	7.905450	-67.479961
Tupinambis teguixin	PM043	-	-	KM259771	KM259675	Agropecuaria Puerto Miranda	7.905450	-67.479961
Tupinambis teguixin	RN098	-	-	KM259821	KM259725	San Carlos de Rio Negro, Amazonas State	1.918953	-67.055395
Tupinambis teguixin	RN099	-	-	KM259822	KM259726	San Carlos de Rio Negro, Amazonas State	1.918953	-67.055395
Tupinambis teguixin	RN100	-	-	KM259823	KM259727	San Carlos de Rio Negro, Amazonas State	1.918953	-67.055395
Tupinambis teguixin	RN101	-	-	KM259824	KM259728	San Carlos de Rio Negro, Amazonas State	1.918953	-67.055395
Tupinambis teguixin	RN102	-	-	KM259825	KM259729	San Carlos de Rio Negro, Amazonas State	1.918953	-67.055395
Tupinambis teguixin	ZUL69	-	-	KM259796	KM259700	Campo Rosario, Zulia State	9.391212	-72.993297
Tupinambis teguixin	ZUL70	-	-	KM259797	KM259701	Campo Rosario, Zulia State	9.391212	-72.993297
Tupinambis teguixin	ZUL71	-	-	KM259798	KM259702	Campo Rosario, Zulia State	9.391212	-72.993297
Tupinambis teguixin	ZUL72	-	-	KM259799	KM259703	Campo Rosario, Zulia State	9.391212	-72.993297
Tupinambis teguixin	ZUL75	-	-	KM259800	KM259704	Campo Rosario, Zulia State	9.391212	-72.993297
Tupinambis teguixin	ZUL76	-	-	KM259801	KM259705	Campo Rosario, Zulia State	9.391212	-72.993297
Tupinambis teguixin	ZUL77	-	-	KM259802	KM259706	Campo Rosario, Zulia State	9.391212	-72.993297
Tupinambis teguixin	ZUL78	-	-	KM259803	KM259707	Campo Rosario, Zulia State	9.391212	-72.993297
Tupinambis teguixin	ZUL79	-	-	KM259804	KM259708	Campo Rosario, Zulia State	9.391212	-72.993297
Tupinambis teguixin	ZUL80	-	-	KM259805	KM259709	Campo Rosario, Zulia State	9.391212	-72.993297
Tupinambis teguixin	ZUL81	-	-	KM259806	KM259710	Campo Rosario, Zulia State	9.391212	-72.993297
Tupinambis teguixin	ZUL82	-	-	KM259807	KM259711	Campo Rosario, Zulia State	9.391212	-72.993297
Tupinambis teguixin	ZUL83	-	-	KM259808	KM259712	Campo Rosario, Zulia State	9.391212	-72.993297
Tupinambis teguixin	ZUL84	-	-	KM259809	KM259713	Campo Rosario, Zulia State	9.391212	-72.993297
Tupinambis teguixin	ZUL85	-	-	KM259810	KM259714	Campo Rosario, Zulia State	9.391212	-72.993297
Tupinambis teguixin	ZUL87	-	-	KM259811	KM259715	Campo Rosario, Zulia State	9.391212	-72.993297
Tupinambis teguixin	ZUL88	-	-	KM259812	KM259716	Campo Rosario, Zulia State	9.391212	-72.993297
Tupinambis teguixin	ZUL89	-	-	KM259813	KM259717	Campo Rosario, Zulia State	9.391212	-72.993297
Tupinambis teguixin	ZUL90	-	-	KM259814	KM259718	Campo Rosario, Zulia State	9.391212	-72.993297
Tupinambis teguixin	ZUL91	-	-	KM259815	KM259719	Campo Rosario, Zulia State	9.391212	-72.993297

Tupinambis teguixin	ZUL92	-	-	KM259816	KM259720	Campo Rosario, Zulia State	9.391212	-72.993297
Tupinambis teguixin	ZUL93	-	-	KM259817	KM259721	Campo Rosario, Zulia State	9.391212	-72.993297
Tupinambis teguixin	ZUL94	-	-	KM259818	KM259722	Campo Rosario, Zulia State	9.391212	-72.993297
Tupinambis teguixin	ZUL95	-	-	KM259819	KM259723	Campo Rosario, Zulia State	9.391212	-72.993297
Tupinambis teguixin	ZUL97	-	-	KM259820	KM259724	Campo Rosario, Zulia State	9.391212	-72.993297
