# Supplementary material for: Cryptic, Sympatric Diversity in Tegu Lizards of the Tupinambis teguixin Group (Squamata, Sauria, Teiidae) and the Description of Three New Species
Source: PLoS One. 2016 Aug 3;11(8):e0158542. doi: 10.1371/journal.pone.0158542 (PMC4972348; doi:10.1371/journal.pone.0158542)
Supplement: S2 Table — (PDF) [file pone.0158542.s002.pdf]

species	OriginalCountry	Musuem #	State	upper labials		lamella 4th finger	lamellae 4th toe	vertebral rows	sc
cryptus	venezuela	usnm16274(	Falcon	18	14	16	33	104.00	
cryptus	Venezuela	usnm16274!	falcon	18	14				
cryptus	Venezuela	usnm16274-	falcon	18	15	15	33	101.00	
cryptus	Venezuela	uf130028	gurnare	17	15	17.00	34.00	110.00	
cryptus	Venezuela	usnm21712:	monagas	16	13	16	31	101.00	
cryptus	Venezuela	UF47718	cojedes		14	16.00	33.00		
cryptus	Venezuela	UF130026	cojedes	18	16	15.00	36.00	109.00	
cryptus	Venezuela	UF47717	San Jorge	19	14	17.00	32.00	108.00	
cryptus	Venezuela	UF47716	San Jorge	18	13	15.00	33.00	103.00	
cryptus	Venezuela	uf47715	San Jorge	18	16	16.00	37.00	105.00	
cryptus	Venezuela	uf130027	San Jorge	18	16	15.00	38.00	108.00	
cryptus	Venezuela	CM7965	Sucre	17	14	16.00	35.00	100.00	
cryptus	Venezuela	CM7937	Sucre	19	18	17.00	32.00	108.00	
cryptus	Venezuela	CM86912	Tachira	18	14	16.00	32.00	107.00	
cryptus	Trinidad	uwi 2011.41	Caura Valle	18	15	16.00	38.00	103.00	
cryptus	Trinidad	vi2010.12.2	FishingPonc	16	14	17.00	34.00	105.00	
cryptus	Trinidad	MNH13761	Nariva	18	14	15	35	103.00	
cryptus	Trinidad	FMNH4990(	San Rafael	19	16	16.00	33.00	103.00	
cryptus	Trinidad	FMNH4989!	San Rafael		14	16.00	33.00	103.00	
cryptus	Trinidad	usnm32136			14	17	32		
cryptus	Trinidad	usnm17551		17	16	15	32	105.00	
cryptus	Trinidad	usnm 17719		16	14	15	34	102.00	
cryptus	Trinidad	mcz6075		16	15	17.00	33.00	101.00	
cryptus	Trinidad	mcz162861		18	15	17.00	33.00	105.00	
cryptus	Trinidad	AMNH73058						107.00	
cryptus	Trinidad	AMNH1650		18	17	18.00	35.00	110.00	
cryptus	Trinidad	AMNH1649		20	17	17.00	37.00	105.00	
cryptus	Trinidad	AMNH1641		20	15	18.00	35.00	105.00	
cryptus	Trinidad	AMNH1640		20	15	16.00	33.00	105.00	
cryptus	Tobago	IMZ2012.27.42		10	16	18.00	37	103.00	
cryptus	Tobago	mcz12001		18	16	16.00	30.00	105.00	
cryptus	Guyana	MNH140937		18	14	15	32	103	
cryptus	Venezuela	usnm21712	Amazonas	20	15	16	33	108	
cryptus	Venezuela	usnm21712:	Amazonas	19	14	16.00	35.00	100.00	
cryptus	Venezuela	usnm21712:	Amazonas	19	16	14	31	107.00	
cryptus	Venezuela	usnm21712-	Amazonas	14	14	15	35	109.00	
cryptus	Venezuela	usm217125	Amazonas	18	14	17	39	104.00	
cryptus	Venezuela	usnm21712(	Amazonas	17	14	16	35	110.00	
cryptus	Venezuela	USNM21713	Bolivar	18	16	16.00	36.00	104.00	
cryptus	venezuela	USNM21713	Bolivar	19	16	14	31	104.00	
cryptus	Venezuela	USNM21712	Bolivar	21	16	13.00	35.00	111.00	

cryptus	Venezuela	USNM21712	Bolivar	18	**14**	**17**	**31**	**103.00**
cryptus	Venezuela	USNM21713	Orinoco	16	**16**	**15**	**32**	**112**
cryptus	Venezuela	USNM21713	Orinoco	20	**15**	**15**	**35**	**113**
cryptus	Venezuela	USNM21713	Orinoco	19	**14**	**15**	**32**	**107.00**
cryptus		NRM 123		16	11	16	34	108
cryptus		NRM 120		14	14	16	33	110
cryptus		NRM 121		16	12	16	33	102
cryptus	Suriname	NRM 5052	Suriname	14	12	17	32	105
cryptus	Trinidad	BMNH 190	Ilha Trinida	17	15	16	33	105
cryptus	América dc	BMNH 52.9	América dc	18	12	18	35	105
cryptus	Suriname	NRM 5052	Suriname	14	12	14	32	106
cryptus	Surniame	BMNH 194	Paramaribc	18	14	17	34	107
cryptus	Guyana	BMNH 193	Mazaruni, I	18	15	16	34	107
cryptus	Suriname	NRM 1470	Suriname	14	12	16	31	111
cryptus		NRM 1471		16	12	17	31	103
cryptus	Brasil	BMNH s/n	São Gabrie	16	12	18	35	111
cryptus		NRM 1473		18	12	17	34	102
cryptus		NRM 121		15	11	17	36	110
cryptus	Brazil,	CHUNB 152	Amapá	14	12	15	36	106
cryptus	Brazil,	CHUNB 152	Amapá	14	10	15	30	112
cryptus	Brazil,	MUZUSP 27461		14	12	14	28	112
cryptus	Guyana	BMNH 56.5	Demerara,	16	13	15	35	102
cryptus	América dc	BMNH 52.9	América dc	16	12	17	33	104
cryptus	América dc	BMNH 111	América dc	17	13	17	31	104
cryptus	Suriname	BMNH 194	Paramaribc	18	15	16	35	111
cryptus	Guyana	BMNH 197	Holmia, Bri	18	15	16	33	111
cryptus	América dc	BMNH 52.9	América dc	16	13	16	32	111
cryptus	Tobago	BMNH 192	Ilha Tobagc	17	14	15	34	103
cryptus	América dc	BMNH 111	América dc	18	12	17	34	105
cryptus		NRM 122		16	12	17	34	106
cryptus	Guyana	BMNH 56.5	Demerara,	17	14	16	35	106
cryptus	Brasil	BMNH 111	Brasil	17	12	17	34	106
cryptus	Suriname	NRM 1469	Suriname	15	13	16	31	107
cryptus	Brasil	MUZUSP 79	Fazenda Sa	14	12	15	33	108
cryptus	Brasil	MUZUSP 4	Costa da Sa	14	12	17	32	109
cryptus		NRM 1472		14	13	17	34	111
cryptus	Brasil	BMNH 199	Ilha de Mar	17	13	17	33	103
cryptus	Tobago	BMNH 192	Ilha Tobagc	18	15	16	31	103
cryptus	Brasil	CHUNB 013	Amapá	14	13	16	32	108
cryptus	Maloca Ma	MUZUSP 70	Maloca Ma	14	12	16	33	110
cryptus	Altamira, P	MUZUSP 60	Altamira, P	14	12	15	33	111
cryptus	?	MACNBR 1	Ilha Trinida	12	12	13	29	104
cryptus	América dc	BMNH 52.9	América dc	16	12	15	32	108
cryptus		NRM 1466		14	13	17	35	110
cryptus	Brasil	MUZUSP 45	Catrimani,	16	12	16	34	111
cryptus	Brasil	CHUNB 107	Santa Terez	15	11	18	35	111
cryptus		NRM 1467		16	13	17	31	111

cryptus	MACNBR te	MACNBR te	MACNBR te	14	14	17	37	112
cryptus	Brasil	CHUNB 140	Chapada do	14	12	17	35	111
cryptus	Brasil	BMNH 111	Brasil	17	15	14	33	112
cryptus	Brasil	CHUNB 152	Amapá	14	10	16	31	112
cryptus	Brasil	MUZUSP 25	Moura, AM	14	12	17	34	112
cryptus	Brasil	CHUNB 152	Amapá	14	10	16	38	106
cryptus	Brasil	MUZUSP 7.	Santa Mari	14	12	16	35	106
cryptus	Brasil	MUZUSP 7.	Santa Mari	14	12	15	31	108
cryptus		NRM 1468		17	10	17	33	108
cryptus	América do	BMNH 52.9	América do	16	13	15	31	108
cryptus	Brasil	CHUNB 107	Santa Terez	14	10	16	38	110
cryptus	Brasil	CHUNB 140	Chapada do	14	10	16	34	111
cryptus	Brasil	MUZUSP 5!	Manaus, AI	14	12	17	37	112
cryptus	Brasil	MUZUSP 1:	Marowijne	14	10	16	31	112
cryptus	Brasil	CHUNB 107	Santa Terez	12	10	16	33	112
cryptus	Brasil	CHUNB 140	Chapada do	14	10	14	32	105
cryptus	Tobago	BMNH 193	Ilha Tobago	18	14	16	32	106
cryptus	Guyana	BMNH 193	Mazaruni, I	19	15	15	34	109
cryptus	Brasil	CHUNB 161	Palmas, TO	14	11	18	34	110
cryptus	Brasil	MUZUSP 48	Dumbá, MT	14	12	16	35	111
cryptus	Brasil	MUZUSP 7:	Santa Mari	14	12	16	33	112
cryptus	Brasil	CHUNB 148	Chapada do	14	11	15	35	105
cryptus	Brasil	MUZUSP 5!	Amolar, MS	14	13	17	35	108
cryptus	Brasil	BMNH 199	Ilha de Mar	17	13	17	33	110
cryptus	Guyana	BMNH 3.2.	Demerara,	18	16	16	32	111
cryptus	Brasil	CHUNB 107	Santa Terez	14	11	17	35	112
cryptus	Brasil	MUZUSP 2:	Barreirinha	14	12	16	35	112
cryptus	Brasil	BMNH 96.6	Itacoatiara,	17	14	16	35	107
cryptus	Brasil	CHUNB 152	Amapá	14	12	14	34	109
cryptus	Brasil	CHUNB 140	Chapada do	14	10	16	33	110
cryptus	Brasil	MUZUSP 60	Maloca Ma	14	12	17	32	112
cryptus	Brasil	MUZUSP 3:	Barcelos, A	14	12	15	36	112
cryptus	Brasil	MUZUSP 2:	Barreirinha	14	12	17	33	112
cryptus	Brasil	MUZUSP 7:	Catrimani,	14	12	17	34	103
cryptus	Brasil	MUZUSP 7:	Caracaraí, I	14	12	17	34	107
cryptus	Brasil	MUZUSP 4'	Costa da Sa	16	12	16	33	109
cryptus	Brasil	CHUNB 140	Chapada do	13	11	14	34	109
cryptus	Brasil	MUZUSP 3'	Barra do Co	14	12	16	32	109
cryptus	Brasil	MUZUSP 2:	Belém, PA	14	12	16	36	110
cryptus	Brasil	MUZUSP 1'	Itapiranga,	14	12	17	34	110
cryptus	Brasil	MUZUSP 79	Serra de Ku	14	12	17	35	111
cryptus	Brasil	CHUNB 148	Chapada do	14	11	16	33	112
cryptus	Brasil	CHUNB 107	Santa Terez	15	11	18	37	112
cryptus	Brasil	CHUNB 140	Chapada do	13	11	16	35	112
cryptus	Brasil	MUZUSP 7:	Caracaraí, I	14	12	15	34	110
cryptus	Brasil	CHUNB 107	Santa Terez	14	8	16	34	111
cryptus	Brasil	MUZUSP 79	Normandia	15	12	17	32	111

cryptus	Brasil	MUZUSP 1	Oriximiná,	14	12	16	36	112
cryptus	Brasil	CHUNB 01	Amapá	14	11	14	31	106
cryptus	Brasil	CHUNB 10	Santa Tere	14	10	18	35	112
cryptus	Brasil	MUZUSP 4	Limoeiro, A	12	12	18	39	110
cryptus	Brasil	CHUNB 10	Santa Tere	14	11	16	34	110
cryptus	Brasil	CHUNB 14	Chapada d	14	9	17	33	112
cryptus	Brasil	CHUNB 14	Chapada d	14	10	16	35	112
cryptus	Brasil	CHUNB 01	Amapá	17	14	16	32	107
cryptus	Guyana	BMNH 53.	Berbice, Gu	16	14	17	37	110
cryptus	Brasil	MUZUSP 3	Barcelos, A	14	12	17	36	109
cryptus	Brasil	CHUNB 14	Chapada d	14	12	15	35	112
cryptus	Brasil	CHUNB 01	Amapá	15	16	17	32	107
cryptus	Brasil	CHUNB 16	Palmas, TO	14	10	17	35	111
cryptus	Brasil	CHUNB 01	Amapá	14	15	16	34	111
cuzcoensis	Ecuador	MNH218511		16	**14**	**16**	**33**	**119**
cuzcoensis	Peru	MNH16833	MadreDios	18	**14**	**15**	**35**	**114**
cuzcoensis	Peru	FMNH8137	Cuzco	18	**12**	**14**	**32**	**107**
cuzcoensis	Peru	MNH16825	Cuzco	18	**14**	**14**	**32**	**120**
cuzcoensis	Peru	MNH16826	Cuzco	16	**14**	**14**	**34**	**121**
cuzcoensis	Peru	MNH 16822	Cuzco	15	**14**	**16**	**33**	**120**
cuzcoensis	Peru	mnh16833	MadreDios		**14.00**	17.00	34.00	119.00
cuzcoensis	Ecuador	NRM RBB	Zamora, Eq	15	8	16	34	115
cuzcoensis	Oriente, A	MUZUSP 3	Oriente, A	14	12	16	36	116
cuzcoensis	Porto Walt	MUZUSP 5	Porto Walt	14	13	15	32	120
cuzcoensis	Peru	BMNH 81.	Pampa del	17	14	16	34	120
cuzcoensis	CHUNB 00	CHUNB 00	CHUNB 00	14	14	18	29	120
cuzcoensis	Brasil	MUZUSP 5	Benjamin C	14	10	16	33	123
cuzcoensis	Brasil	MUZUSP 2	Alto Purus,	14	12	16	34	124
cuzcoensis	Ecuador	NRM 3914	Rio Pastaza	15	8	16	32	115
cuzcoensis	Brasil	MUZUSP 1	Santo Antô	15	12	16	36	116
cuzcoensis	?	MUZUSP 5	MUZUSP 5	22	16	16	32	138
cuzcoensis	Brasil	MNRJ R26	Rio Javari,	13	8	17	39	123
teguixin		marmoratus						115
teguixin		nrm13			**16**			**116**
teguixin	Surinam	MNH12108	Paramiabo	17	**16**	**15**	**33**	**116**
teguixin	Guyana	MNH140938		18	**14**	**16**	**31**	**101**
teguixin	Guyana	MNH12651	Demerara	19	**16**	**14**	**34**	
teguixin	Guyana	MNH12651	Demerara	18	**15**			
teguixin	Guyana	MNH12650	Essequibo	16	**16**	**15**	**34**	**112**
teguixin	Guyana	MNH126508		18	**16**	**14**	**34**	**116**
teguixin	Guyana	MNH12651	Demerara	16	**14**		**34**	**110**
teguixin	Guyana	MNH12651	Demerara	18	**16**	**16**	**36**	**113**
teguixin	Guyana	MNH126518			16	**15**	**33**	**110**
teguixin	Surinam	MNH133345		16	**14**	**13**	**34**	**111**
teguixin	?	MUZUSP 7	MUZUSP 7	14	12	16	32	114
teguixin	?	MUZUSP 4	MUZUSP 4	14	15	14	29	116
teguixin	Brasil	MUZUSP 7	Catrimani,	14	12	16	33	116

teguixin	Brasil	CHUNB 140	Chapada do	15	10	16	36	116
teguixin	América do	BMNH 52.9	América do	16	13	17	33	114
teguixin	?	MUZUSP 47	MUZUSP 47	14	12	17	35	114
teguixin	Brasil	MUZUSP 48	São Doming	15	12	17	34	116
teguixin	?	MUZUSP 82	MUZUSP 82	14	12	17	36	113
teguixin	Brasil	MUZUSP 30	Diauarum,	15	12	16	33	114
teguixin	Brasil	MUZUSP 63	MUZUSP 63	14	12	17	34	115
teguixin	Brasil	CHUNB 140	Chapada do	14	10	15	35	116
teguixin	Brasil	MNRJ R260	Rio Javari, /	11	12	16	32	120
teguixin	Brasil	CHUNB 107	Santa Terez	14	10	18	33	113
teguixin	Brasil	MUZUSP 73	Santa Mari	14	12	17	33	113
teguixin	Brasil	CHUNB 140	Chapada do	15	11	15	35	113
teguixin	Brasil	CHUNB 107	Santa Terez	14	10	17	35	114
teguixin	Brasil	CHUNB 140	Chapada do	14	13	16	34	115
teguixin	Brasil	MUZUSP 48	São Félix do	14	14	17	36	116
teguixin	Brasil	MNRJ R260	Rio Javari, /	12	13	17	34	125
teguixin	Brasil	MUZUSP 48	Mato Verde	15	12	16	36	113
teguixin	Brasil	CHUNB 107	Santa Terez	14	10	18	36	114
teguixin	Brasil	MUZUSP 50	Manaus (IN	14	12	16	34	116
teguixin	Brasil	MUZUSP 81	Cláudia, MT	14	12	17	36	116
teguixin	Brasil	CHUNB 107	Santa Terez	15	10	15	35	117
teguixin	Brasil	CHUNB 107	Santa Terez	15	10	17	34	118
teguixin	Brasil	CHUNB 107	Santa Terez	14	11	16	36	113
teguixin	Brasil	MUZUSP 21	MUZUSP 21	14	12	17	35	114
teguixin	Brasil	CHUNB 148	Chapada do	15	10	16	35	114
teguixin	Brasil	CHUNB 107	Santa Terez	13	10	17	36	114
teguixin	Brasil	CHUNB 161	Palmas, TO	14	10	18	36	115
teguixin	Brasil	MUZUSP 11	Oriximiná,	14	12	19	38	121
teguixin	Brasil	MUZUSP 17	MUZUSP 17	15	12	14	34	113
teguixin	Brasil	BMNH 192	Manaus, AI	16	14	18	34	114
teguixin	Brasil	CHUNB 107	Santa Terez	14	10	18	36	114
teguixin	?	MUZUSP 21	MUZUSP 21	15	12	17	36	115
teguixin	Brasil	CHUNB 107	Santa Terez	15	10	18	33	115
teguixin	Brasil	CHUNB 166	Amapá	14	10	16	33	116
teguixin	Brasil	MUZUSP 82	São Miguel	14	12	15	35	117
teguixin	Brasil	CHUNB 107	Santa Terez	14	10	17	34	119
teguixin	Brasil	CHUNB 140	Chapada do	14	11	16	33	119
teguixin	Brasil	MUZUSP 83	MUZUSP 83	15	12	16	35	120
teguixin	Brasil	CHUNB 107	Santa Terez	16	12	18	35	121
teguixin	Brasil	CHUNB 107	Santa Terez	14	10	18	35	126
teguixin	Brasil	CHUNB 107	Santa Terez	14	10	17	37	115
teguixin	Brasil	CHUNB 148	Chapada do	14	11	15	34	115
teguixin	Brasil	MUZUSP 48	Mato Verde	15	12	18	33	116
teguixin	Brasil	MUZUSP 83	MUZUSP 83	14	12	14	36	116
teguixin	Brasil	MUZUSP 60	Lago Aman	15	12	17	35	118
teguixin	Brasil	CHUNB 152	Amapá	14	10	16	36	119
teguixin	Brasil	MUZUSP 55	Amolar, MS	14	12	16	34	113

teguixin	Brasil	CHUNB 107	Santa Terez	14	10	17	36	115
teguixin	Brasil	MUZUSP 1	Itapiranga,	14	12	18	34	116
teguixin	Brasil	CHUNB 107	Santa Terez	14	11	18	36	116
teguixin	Brasil	MUZUSP 8	Cláudia, MT	14	14	16	34	119
teguixin	Brasil	BMNH 191	Canelos, Ec	15	13	16	32	120
teguixin	Brasil	MUZUSP 8	MUZUSP 8	14	12	17	34	120
teguixin	Brasil	CHUNB 107	Santa Terez	15	11	18	36	113
teguixin	Brasil	MUZUSP 6	Ilha do Ban	14	10	18	37	115
teguixin	Brasil	MUZUSP 4	MUZUSP 4	14	12	16	36	115
teguixin	Brasil	CHUNB 148	Chapada do	14	10	16	36	117
teguixin	Brasil	CHUNB 148	Chapada do	14	9	17	33	118
teguixin	Brasil	CHUNB 107	Santa Terez	14	11	15	35	119
teguixin	Brasil	MUZUSP 6	Aruanã, Go	14	12	17	35	114
teguixin	Brasil	CHUNB 16	Palmas, TO	14	10	18	38	114
teguixin	Brasil	CHUNB 148	Chapada do	12	12	16	36	116
teguixin	Brasil	CHUNB 107	Santa Terez	15	10	18	38	117
teguixin	Brasil	MUZUSP 9	Araguatins,	14	12	18	35	119
teguixin	Brasil	MUZUSP 4	Maraã, AM	14	12	18	39	120
teguixin	Brasil	CHUNB 140	Chapada do	14	10	17	35	123
teguixin	Brasil	MUZUSP 7	Boa Vista, I	14	12	17	34	114
teguixin	Brasil	MUZUSP 3	Santa Mari	14	12	15	33	116
teguixin	Brasil	CHUNB 152	Amapá	14	10	17	34	116
teguixin	Brasil	MUZUSP 3	Barra do Co	14	12	15	31	113
teguixin	Brasil	MUZUSP 6	Araguacem	14	12	17	34	116
teguixin	Brasil	CHUNB 107	Santa Terez	14	10	17	37	116
teguixin	Brasil	CHUNB 107	Santa Terez	14	10	16	34	116
teguixin	Brasil	CHUNB 16	Palmas, TO	14	10	18	35	119
teguixin	Brasil	MUZUSP 8	Cláudia, MT	15	12	14	32	120
teguixin	Brasil	CHUNB 107	Santa Terez	14	11	18	35	113
teguixin	Brasil	MUZUSP 7	Belém, PA	14	12	16	33	113
teguixin	Brasil	MUZUSP 8	Ilha de Mar	14	14	16	33	113
teguixin	Brasil	CHUNB 107	Santa Terez	14	10	16	36	114
teguixin	Brasil	CHUNB 004	Boa Vista, I	14	14	17	34	114
teguixin	Brasil	CHUNB 107	Santa Terez	14	12	18	38	115
teguixin	Brasil	CHUNB 16	Palmas, TO	14	10	17	35	119
teguixin	Brasil	CHUNB 107	Santa Terez	14	12	16	37	112
teguixin	Brasil	CHUNB 107	Santa Terez	14	10	16	37	116
teguixin	Brasil	CHUNB 107	Santa Terez	14	11	17	37	113
teguixin	Brasil	MACNBR te	MACNBR te	14	13	19	36	113
teguixin	Brasil	CHUNB 107	Santa Terez	14	10	17	37	117
teguixin	Brasil	MNRJ R174	Belém, PA	11	12	18	35	117
teguixin	Brasil	MUZUSP 2	Manaus, AI	14	12	17	38	118
teguixin	Brasil	CHUNB 107	Santa Terez	14	9	17	37	115
teguixin	Brasil	BMNH 194	Paramaribo	18	14	17	32	118
teguixin	Brasil	MUZUSP 3	Canutama,	14	12	19	37	118
teguixin	Brasil	MUZUSP 5	Taboleiro L	14	12	16	34	112
teguixin	Brasil	CHUNB 107	Santa Terez	17	10	17	35	115

teguixin	Brasil	CHUNB 004	Boa Vista, I	14	14	17	32	112
teguixin	Brasil	CHUNB 107	Santa Tere:	14	10	17	34	118
teguixin	Brasil	CHUNB 148	Chapada do	14	10	16	35	118
teguixin	Brasil	CHUNB 004	Boa Vista, I	14	15	19	36	118
zuliensis	Venezuela	FMNH2599	Inconstrado	15	**12**	**16.00**	**34.00**	**110**
zuliensis	Venezuela	FMNH2599	Inconstrado	16	**12**	**16.00**	**34.00**	**110.00**
zuliensis	Venezuela	FMNH2599	Inconstrado	15	**13**	**15.00**	**36.00**	**111.00**
zuliensis	Venezuela	FMNH2599	Inconstrado	16	**14**	**15**	**36**	**113.00**
	Brazil,	ɔpunctatus	Para			13	Mar-35	118
	Brazil,	ɔpunctatus	Para	7	**nd**	13	35	119

	Musuem #	OriginalCountry	State	Sex	SVL	tail	leglength	hl/svl
	nigropunctatus	Brazil,	Para	m				
teguixin	marmoratus							
teguixin	nrm13							
teguixin	MNH12108	Surinam	Paramiabo	j	91	d	56	0.62
teguixin	MNH14093	Guyana		j	84	134	d	
teguixin	MNH12651	ritish Guian	Demerara	m	223	431		
teguixin	MNH12651	ritish Guian	Demerara	m	220	d	166	0.75
teguixin	MNH12650	ritish Guian	Essequibo	m	279	491	185	0.66
teguixin	MNH12650	ritish Guiana		m	237	392	152	0.64
teguixin	MNH12651	ritish Guian	Demerara		255	d	158	0.62
teguixin	MNH12651	ritish Guian	Demerara	f	245	476	155	0.63
teguixin	MNH12651	ritish Guiana		f	275	442	172	0.63
teguixin	MNH13334	Surinam		j	131	234	86	0.66
	nigropunctatus	Brazil,	Para	m				
cryptus	usnm16274	venezuela	Falcon	f	284	d		
cryptus	usnm16274	Venezuela	falcon	f	272	d	187	0.69
cryptus	usnm16274	Venezuela	falcon	j	196	319	148	0.76
cryptus	**uf130028**	**Venezuela**	**gurnare**	**f**	**163**	**295+**		
cryptus	**usnm21712**	**Venezuela**	**monagas**	**j**	**114**	**175+**	**77**	0.68
cryptus	UF47718	Venezuela	cojedes	f	232	454		
cryptus	UF130026	Venezuela	cojedes	f	205	384		

cryptus	**UF47717**	**Venezuela**	**San Jorge**		**263**	**488**		
cryptus	**UF47716**	**Venezuela**	**San Jorge**		**295**	**586**		
cryptus	**uf47715**	**Venezuela**	**San Jorge**	**m**	**325**	**d**		
cryptus	**uf130027**	**Venezuela**	**San Jorge**	**m**	**175**	**340**		
cryptus	**CM7965**	**Venezuela**	**Sucre**	**f**	**275**	**545+**		
cryptus	**CM7937**	**Venezuela**	**Sucre**	**m**	**278**	**635**		
cryptus	**CM86912**	**Venezuela**	**Tachira**	**m**	**310**	**625**		
cryptus	uwi 2011.41	Trinidad	Caura Valley		214	486		
cryptus	vi2010.12.2	Trinidad	FishingPonc	m	263	463	135	
cryptus	MNH13761	Trinidad	Nariva	j	95	d		
cryptus	FMNH4990(	Trinidad	San Rafael	m	266	511	160	0.60
cryptus	FMNH4989!	Trinidad	San Rafael	f	183	d		
cryptus	usnm32136	Trinidad		m	292	593	176	0.60
cryptus	usnm17551	Trinidad		m	285	d	185	0.65
cryptus	usnm 1771!	Trinidad		m	265	d	147	0.55
cryptus	mcz6075	Trinidad		m	272	584		
cryptus	mcz162861	Trinidad		j	132	215		
cryptus	AMNH7305{	Trinidad		j	85	142		
cryptus	AMNH1650	Trinidad			183	354		
cryptus	AMNH1649	Trinidad			252	360		
cryptus	AMNH1641	Trinidad			182	324		
cryptus	AMNH1640	Trinidad			192	360		
cryptus	IMZ2012.2	Tobago		j	106	178		
cryptus	mcz12001	Tobago		m	203	285d		
cryptus	MNH14093	Guyana		m	340	d	188	0.55
cryptus	usnm21712	Venezuela	Amazonas	m	263	511+	167	0.63
cryptus	usnm21712	Venezuela	Amazonas	m	219	d	150	0.68
cryptus	usnm21712	Venezuela	Amazonas	j	185	337	72	0.39
cryptus	usnm21712	Venezuela	Amazonas	f	232	d	162	0.70
cryptus	usm217125	Venezuela	Amazonas	m	285	465d	162	0.57
cryptus	usnm21712	Venezuela	Amazonas	m	257	490+	157	0.61
cryptus	USNM21713	Venezuela	Bolivar	m	195	d	129	0.66
cryptus	USNM21713	venezuela	Bolivar	f	230	d	148	0.64
cryptus	USNM21712	Venezuela	Bolivar	f	183	d	125	0.68
cryptus	USNM21712	Venezuela	Bolivar	f	282	605	180	0.64
cryptus	USNM21713	Venezuela	Orinoco	m	216	d		0.00
cryptus	USNM21713	Venezuela	Orinoco	m	391	53	187	0.48

cryptus	ISNM21713	Venezuela	Orinoco	m	262	626	161	0.61
zuliensis	FMNH2599l	Venezuela	nconstrado	m	244	d	155	0.64
zuliensis	FMNH2599	Venezuela	nconstrado	m	147	d	96	0.65
zuliensis	FMNH2599	Venezuela	nconstrado	m	273	537	175	0.64
zuliensis	FMNH2599	Venezuela	nconstrado	m	168	d	94	0.56
cuzcoensis	MNH21851	Ecuador		j	120	d	82	0.68
cuzcoensis	MNH16833	Peru	MadreDios	j	94	140	67	0.71
cuzcoensis	FMNH8137	Peru	Cuzco	j	121	210	82	0.68
cuzcoensis	MNH16825	Peru	Cuzco	j	85	d	63	0.74
cuzcoensis	MNH16826	Peru	Cuzco	j	87	143	62	0.71
cuzcoensis	MNH 16822	Peru	Cuzco	m	247	d		
cuzcoensis	mnh16833	Peru	MadreDios	m	95.00	155.00	63.00	

ales around mid body	transverse ventral rows	longitudinal ventral rows
**108.00**	**31**	24
		24.00
**111**	**30.00**	24.00
**111.00**	**28.00**	**25.00**
	**30.00**	**24**
**102.00**	**27.00**	23.00
**101.00**	**28.00**	24.00
**104.00**	**28.00**	**25.00**
**101.00**	**28.00**	**26.00**
**105.00**	**28.00**	**21.00**
**109.00**	**28.00**	**23.00**
**105.00**	**28.00**	**23.00**
**106.00**	**30.00**	**24.00**
**100.00**	**28.00**	**24.00**
**103.00**	**31.00**	25.00
**113.00**	**29.00**	25.00
**108.00**	**30.00**	23.00
**108.00**	**28.00**	22.00
**105.00**	**28.00**	22.00
**105.00**	**30.00**	**21**
**109.00**	**28**	25.00
**107.00**	**d**	24
**112.00**	**31.00**	22.00
**103.00**	**29.00**	24.00
**113.00**	**30.00**	23.00
**109.00**	**32.00**	24.00
**113.00**	**29.00**	21.00
**105.00**	**29.00**	22.00
**94.00**	**34.00**	24.00
**101.00**	**31.00**	25.00
**101**	**31**	24
**115**	**28**	26.00
**103.00**	**29.00**	22.00
**103.00**	**31**	23
	**29**	21.00
**108.00**	**31**	24.00
**104.00**	**27**	22
**100.00**	**31.00**	25.00
**109.00**	**30.00**	26
**106.00**	**29**	22

**107.00**	**31**	26
**120.00**	**31**	24
**109**	**29**	26
**112**	**31**	24
93	34	22
93	34	24
94	33	23
94	33	25
97	33	23
97	35	23
97	33	23
97	35	25
97	37	24
97	33	23
98	35	27
98	36	26
99	34	24
99	33	24
100	35	23
100	33	23
100	33	27
101	34	23
101	34	22
101	37	25
101	34	22
101	35	24
101	35	24
102	34	22
102	33	24
102	33	25
102	34	24
102	35	22
102	34	24
102	35	22
102	33	24
102	34	20
103	34	23
103	34	23
103	35	23
103	34	24
103	34	25
104	34	24
104	34	22
104	34	24
104	33	24
104	34	24
104	35	24

104	36	26
105	36	24
105	34	24
105	34	25
105	35	24
106	34	26
106	35	21
106	33	24
106	34	24
106	34	23
106	35	25
106	34	23
106	33	26
106	33	26
106	34	23
107	34	23
107	32	25
107	38	26
107	35	23
107	35	23
107	33	26
108	34	25
108	35	25
108	35	24
108	35	22
108	33	24
108	33	26
109	34	24
109	33	25
109	34	24
109	33	25
109	34	24
109	36	26
110	35	25
110	34	26
110	33	24
110	34	23
110	35	25
110	35	23
110	35	26
110	33	24
110	34	24
110	35	25
110	35	23
111	35	25
111	34	27
111	35	25

111	35	26
112	36	26
112	34	23
113	35	25
113	36	23
113	34	23
113	35	23
114	35	27
114	36	22
115	35	25
115	34	24
116	35	26
118	36	27
120	35	25
**101**	29	**24**
**94**	30	**20**
**95**	30	**21**
**97**	29	**20**
**99**	**29**	22
**96**	28	**21**
93.00	28.00	19.00
94	34	22
95	32	23
96	32	22
96	32	23
98	33	23
98	33	23
98	32	25
99	33	23
99	33	26
99	36	27
99	32	24
**100**	**30**	22
**103**	**30**	22
**101**	**30**	23
**102**	**32**	22
**101**	**29**	22
**100**	**30**	21
**108**	**29**	22
**99**	**29**	23
**106**	**31**	20
100	34	22
100	34	22
101	34	23

102	35	22
103	34	23
103	35	24
103	35	23
104	34	23
104	34	24
104	35	25
104	34	24
104	32	22
105	35	25
105	35	23
105	35	23
105	33	22
105	35	24
105	33	25
105	34	24
106	35	25
106	35	26
106	35	23
106	35	23
106	36	23
106	35	25
107	34	24
107	34	25
107	34	22
107	36	23
107	34	24
107	35	25
108	33	24
108	35	27
108	35	25
108	35	24
108	35	25
108	35	25
108	35	25
108	34	24
108	34	21
108	34	22
108	35	25
108	38	23
109	34	23
109	36	23
109	34	24
109	34	25
109	35	25
109	35	24
110	36	26

110	36	24
110	35	25
110	36	24
110	35	24
110	32	25
110	35	21
111	35	27
111	34	24
111	36	23
111	35	24
111	35	24
111	35	26
112	35	24
112	35	29
112	23	23
112	36	25
112	34	25
112	36	24
112	34	24
113	34	23
113	34	24
113	35	27
114	35	26
114	34	28
114	35	26
114	35	24
114	34	24
114	35	26
115	34	25
115	35	23
115	35	26
115	34	27
115	37	23
115	35	24
115	35	24
116	35	27
116	35	26
117	34	23
117	36	27
117	34	27
117	37	26
117	36	24
118	35	25
118	34	25
118	35	24
119	36	27
119	35	25

120	33	25
121	36	26
121	36	26
124	34	26
**101.00**	**29.00**	22.00
**100.00**	**28.00**	22.00
**102.00**	**28.00**	22.00
**108.00**	**28**	22
107	32	20
107	32	20

	occipitalIP	*interangular sulcus*	*intertypanic sulcus*	*patch of Antegulars*	*scales @ 3-4 fingers*	*1st labial shorter than 2nd*	*1st labial longer than 2nd*	*labials@ loreal*
	4.00	0.00	1.00					
	3.00							23.00
	3.00							
	2.00	i	1.00					
a	3.00	i	1.00	y	4.00	y	y	23.00
a		i	1.00	y	4.00	y	e	23.00
a	3.00	i	1.00	y	8.00	y	e	23.00
a	3.00	i	1.00	y	4.00	y	e	23.00
a	3.00	i	1.00	y	d	y	e	23.00
a	3.00	i	1.00	y	6.00	y	n	23.00
a	3.00	i	1.00	y	7.00	y	n	23.00
a	3.00	i	1.00	y	7.00	y	y	23.00
	4.00	0.00	1.00					
b	1.00	i	1.00	y	4.00	y	y	23.00
b	1.00	i	1.00	y	7.00	y	y	23.00
b	1.00	i	1.00	y	6.00	y	y	23.00
b	1.00	i	1.00					
b	1.00	i	1.00	y	4.00	y	y	23.00
b	1.00	i	1.00					
b		i	1.00					

**b**		i	**1.00**					
**b**		i	**1.00**					
**b**	**1.00**	i	**1.00**					
**b**		i	**1.00**					
**b**		i	**1.00**					
**b**		i	**1.00**					
**b**		i	**1.00**					
b	1.00	i	1.00	7.00	y	n	y	23.00
b	1.00	i	1.00	y		n	y	23.00
b		i	1.00					
b	1.00	i	1.00	y	5.00	y	y	23.00
b	2.00	i	1.00	y	5.00	y	y	23.00
b		i	1.00	y	4.00	y	y	23.00
b	1.00	i	1.00	y	2.00	y	y	23.00
b		i	1.00	y	5.00	y	y	23.00
b		i	1.00					
b		i	1.00					
b		i	1.00					
b		i	1.00					
b		i	1.00					
b		i	1.00					
b		i	1.00					
b	3.00	i	1.00	y		n	n	23.00
b		i	1.00					
b	3.00	i	1.00	y	3.00	y	y	23.00
g	3.00	i	1.00	y	6.00	y	y	23.00
**e**	**3.00**	**fold**	**1.00**	**y**	**4.00**	**y**	**eq**	**23.00**
**e**	**3.00**	**fold**	**1.00**	**y**	**3.00**	**y**	**y**	**23.00**
**e**	**3.00**	**fold**	**1.00**	**y**	**3.00**	**y**	**y**	**23.00**
**e**	**3.00**	**fold**	**1.00**	**y**	**6.00**	**y**	**y**	**23.00**
**e**	**3.00**	**fold**	**1.00**	**y**	**4.00**	**y**	**y**	**23.00**
**f**	**2.00**	**i**	**1.00**	**u**	**8.00**	**y**	**y**	**23.00**
**f**	**1.00**	**i**	**1.00**	**y**	**5.00**	**y**	**y**	**23.00**
**f**	**2.00**	**I, line**	**1.00**	**y**	**5.00**	**y**	**y**	**23.00**
**f**	**1.00**	**i, line**	**1.00**	**y**	**5.00**	**y**	**y**	**23.00**
d	2.00	1.00	1.00	y	6.00	n	y	23.00
d	1.00	1.00	1.00	y	6.00	y	y	23.00

d	1.00	1.00	1.00	n	3.00	y	y	23.00
c	5.00	d	1.00	y	8.00	y	y	23.00
c	3.00	fold	1.00	y	6.00	y	y	23.00
c	2.00	i	1.00	y	4.00	y	y	23.00
c	3.00	i	1.00	y	7.00	y	y	23.00
e								
e								
e	1.00							
e								
e								
e	2.00	i	1.00					
e								

labials @1stSUBOC	loreal fragmented	svl/tail	dorsal row reduction	multiple granular scales	scakes laterfolds	cornerorbit/upp labial	unreduced dorsals	number of supratempor
								2
34.00	n			0		34		
								3
			equal	0	5	3	20	3
34.00	n	0.626866	equal	0	5	3	21	3/2
		0.517401		0			19	3
34.00	n		equal	0	5	3	20	3
34.00	n	0.568228	equal	0	5	3	20	3
34.00	n	0.604592	equal	0	5	34	19	3
34.00	n		equal	1	5	34	22	3
34.00		0.514706	equal	1	5	34	23	3
34.00	n	0.622172	equal	0	5	3	21	3
34.00	y	0.559829	equal	0	5	3	25	2/3
								2
34.00	n			1		4	20	2
34.00	n		1	1	5-6	4	16	2
34.00		0.61442	1	1	5-6	4	19	1
			**equal**	**1**	**5-6**	**4**	**~23**	**2**
34.00	n		**1**	**0**	**5-6**	**4**	**16**	**2**
		0.511013						2
		0.533854					21	3

		**0.538934**							**2**
				**0**				**17**	**2**
								**17**	**2**
		**0.514706**							**3**
									**2**
		**0.437795**							**3**
		**0.496**							**2**
34.00	n	0.440329				4			2
34.00	n	0.568035				4			3
			1						2
34.00	n	0.520548	1	1	5-6	4	16		2
34.00	n			1	6	4	15		2
34.00	n	0.492411	1	1	5-6	4	14		2
34.00	n		1	1	5-6	4	17		2
34.00	n		1`	`1	6	4	16		2
		0.465753							2
		0.613953				4	16		2
		0.598592							
		0.516949							2
		0.7	1	1					2
		0.561728				4			2
		0.533333							2
34.00	n	0.595506				4			2
			1	1	5	4	15		2
34.00	n		1	1	5-6	4	16		3
34.00	n		1	1	6	4	21		2
**34.00**	**n**		1	1	5-6	4	17		2
**34.00**	**n**	0.548961	1`	1	5-6	4	21		2
**34.00**	**n**		1		5-6	4	23		2
**34.00**	**y**		1	1	5	4	20		2
**34.00**	**n**	#VALUE!	1	1	6	4	22		2
**34.00**	**n**		1	1	5	4	24		2
**34.00**	**n**		1	1	5-6	4	17		3
**34.00**	**n**		equal	no	4-5	4	23		2
**34.00**	**n**	0.466116	1	1	5	4	17		2
34.00	n		1	1	56	4	20		2
34.00	y		e	1	5-6	4	16		n

34.00	n	0.41853	1	1	6	4	17	2
34.00	e		1	1	5-6	3	21	3
34.00	e		1	1	6	3	20	3
34.00	e	0.50838	1	1	5-6	3	20	2
34.00	e		1	1	5	3	20	3
			1	1	6	34	25	3
			1	1	6	34	22	3/4
			1	1	5	4	25	4
			1	1	5-6	4	26	4
			e	1	56	34	20	4
								2
			1.00	1.00	5.00	4.00	14.00	4.00

ls<br>upracil @ last supraoc	occiptals longer	**loreal over 1st labial**	**parietals contact # supraoculars**	**dorsolateral line**	**dorsum w/ dashes or spots 4 rows**	**gulars black**	**gulars w/black spots**	**frontal contacs suproc**
**3**	y	y	2		y	n	y	2
**2**								
2	n	n	2	n	y	y	y	2
2	n	no	2	n	y	n-j	y	2
2	n	n	2	n	y	y	y	2
2	n	n	2	n	y	y	y	2
2	n	n	2	n	y	y	n	2
2	n	n	2	n	y	y	n	2
2	n	n	2	n	y	y	y	2
2	n	n	2	weak	y	n	n	2
**2**	n	n	2	weak	y	n	y	2
2	n	no	2	n	y	white	yes	2
**3**	y	y	2		y	n	y	2
3	n	no	3	yes	yes	yes	yes	2
3	n	no	3	yes	yes	yes	yes	2
2	n	no	2	y	y	y	y	2
**3**								
**3**	**n**	**no**	**2**	**spots**	**y**	**yes**	**y**	**2**
2		no	2	no	y	n	y	
3		?	3	dashes	y	yes	y	

2								
3		no	2	spots	y	yes	no	
3		no	2	no	no	yes	no	
3								
3								
3								
3								
3		no	3	no	n	n	y	2
3		no	2	n	n	yes	y	2
3								
3	longer	no	3	no	no	n	no	2
3	shorter	no	3	no	no	n	no	2
3		no	2/3	no	no	yellow	yes	2
3	yt	no	3	no	no	yellow	yes	2
3	eq	no	4/3	no	n	yellow	yes	2
3		no	2			no	no	
2		no	2	maybe	yes	no	yes	
3								
2								
3								
3								
3		no	3	no	y	n	y	2
2		no				n	y	2
3		no	2	weak	n	n	n	2
3	v	n	2	n	n	y	difuse	2
2	y	no	2	weak	y	y	yes	2
2	v	no	2/3	yes	n	yes	NO	2
2	y	n	3	weak	y	y	difuse	2
2	v	no	2	no	yes	yes	yes	2
3	v	no	2	weak	no	y	y	2
3	y	no	3	spots	y	y	y	2
3	n	no	3	y	y	y	y	2
2	n	no	3	y	y	y	y	2
2	n	no	2	weak	y	y	n	3
2	n	no	2	y	y	n	y	2
3	n	no	3	n	y			2

3	n	no	3	y	no	n	y	2
2	n	no	2	no	no	›&Y mottlin	n	2
2	n	no	2	no	no	›&Y mottlin	n	2
2	n	no	2	no	no	›&Y mottlin	n	2
2	n	no	2	no	no	›&Y mottlin	n	2
1	n	no	2	spots	bands	no	no	2
2	n	no	3	spots	bands	no	no	2
2	n	no	3	spots	y+bands	yes	no	2
2	n	no	3	spots	bands	no	no	2
2	n	no	3	spots	bands	no	no	2
1		no	3	spots	weak bands	yes	no	2
2.00	n	n	2.00	spots	weak bands	no	y	2.00

sublabials start at	sublabials	total sublabials	loreal > frontonas	black chin	spots hing legs		*longest supraoc*	vertebral rows
3	**6**		**yes**	**n**		**1**	***12***	118
					**yes**		***1***	115
							2	**116**
3	5/4	9	equal		yes		***2***	**116**
3	4/4	8	EQUAl	n	no		***2***	**101**
3	4/4		equal	y			***1***	
3	5/4	9	eq	y	yes		***1***	
3	3/4	7	equal	y	yes		***1***	**112**
3	4/4	8	equal	y	yes		***1***	**116**
3	3/3	6	equal	y	yes		***1***	**110**
3	4/4	8	equal	n	yes		***1***	**113**
3	4/4	8		n	yes		***1***	**110**
2	4/4	8	shorter	n	yes		***1***	**111**
3	**6**		**yes**	**n**		**1**	***12***	119
4/3	3/2	5	equal	lines			***1***	**104.00**
4/4	3/3	6	e	dl spots	no		***1***	
4/5	3/2	5	longer	dl spots	yes	dash	***1***	**101.00**
**4/4**	**3/4**	**7**	**equal**	**spots**			***1***	**110.00**
**4/3**	**2/2**	**4**	**equal**	**stp**	**yes**	**dash**	***1***	**101.00**
3/3	4/4	8	equal	b + spots			***1***	
4/3	3/3	6	equal	b + spots			***1***	**109.00**

4/4	4/4	8	equal	b + spots			1	108.00
3/3	2/2	4	equal	y			1	103.00
3/3	2/3	5	equal	y			1	105.00
3/4	3/3	6	equal	spots			1	108.00
0.8	4/4	8	equal	spots			1	100.00
	3/3	6	equal	spots	no		1	108.00
1	4/4	8	equal	n			1	107.00
4	4	8	shortrf	n	elongated		1	103.00
4	3/3	6	longer	y	n		1	105.00
1	3/3	6	eq	n			1	103.00
3	4/4	8	equal	n	no		1	103.00
3/4	3/3	6	equal	n			1	103.00
4/4	4/4	8	longer	mot	elong		1	
4/3	4/3	7	longer	bands	no		1	105.00
4/5	3/3	6	equal	y,spots	no		1	102.00
4	4/4	8	longer	n			1	101.00
3	3/4	7	>equal	n	elongated		1	105.00
	4/4	8	eq	n			1	107.00
							1	110.00
							1	105.00
							1	105.00
							1	105.00
4	3/3	6	yes	n	yes		2	103.00
3	4/4	8	>equal	n	elongate		2	105.00
3	4/4	8	e	y	elong		1	103
5	3/2	5	longer	uniform	black spots		1	108
3/3	4/4	8	equal	b+spotst	yes		1	100.00
3	4/4	8	equal	spotst	yes	mottled	1	107.00
3	4/4	8	equal	lines	yes		1	109.00
5	3/3	6	equal	y, spots	yes		1	104.00
3/3	4/4	8	equal	uniform	elongate		1	110.00
3	4/4	8	longer	b/spots	yes	indisct b	2	104.00
4/4	4	8	longer	spotst	yes	indisct b	1	104.00
3/3	5/5	10	equal	y, spots	yes	indisct b	1	111.00
3/3	3/4	7	longer	y	yes	indisct b	1	103.00
3/3	3/3	6	equal	y, spots	elongate		1	112
4	4/4	8	equal	spots			1	113

3/3	4/4	8	equal	spots	elong	indisct b	***1***	**107.00**
3	4/5	9	longer	mot	y		***2***	**110**
3	3/4	7	longer	mot	y		***2***	**110.00**
3	5/4	9	longer	mot	y		***2***	**111.00**
3	4/4	8	longer	mot	y		***2***	**113.00**
5/4	2/2	4	equal	no	n		***2***	**119**
3	3/3	6	longer	no	n		***2***	**114**
3	3/3	6	longer	no	n	n	***2***	**107**
4	3/4	7	longer	no	n		***2***	**120**
4	3/3	6	longer	no	n	n	***2***	**121**
4	3/2	5	equal	yes			***2***	**120**
4.00	2.00	2.00	longer	no	n		***2.00***	119.00

cales around mid body	lamella 4th finger	lamellae 4th toe	transverse ventral rows	longitudinal ventral rows	preanal pores	femoral pores	total pores	number of subocs
107	13	Mar-35	32	20	8	3-7	26	7
								6
								6
100	15	33	30	22	8	13	21	6
103	16	31	30	22	8	16	24	7
101	14	34	30	23	8	13	21	6
					8	16	21	6
102	15	34	32	22	6	15	21	6
101	14	34	29	22	9	11	20	6
100		34	30	21	8	16	24	6
108	16	36	29	22	8	13	21	6
99	15	33	29	23	8	16	21	6
106	13	34	31	20	8	11	19	7
107	13	35/38	32	20	8	3-7	26	7
108.00	16	33	31	24	8			6
				24.00				6
111	15	33	30.00	24.00	11	18	29	6
111.00	17.00	34.00	28.00	25.00				7
	16	31	30.00	24				6
102.00	16.00	33.00	27.00	23.00	9	16	25	d
101.00	15.00	36.00	28.00	24.00	d	20		6

104.00	17.00	32.00	28.00	25.00	8	17	25	6
101.00	15.00	33.00	28.00	26.00	9	18	27	6
105.00	16.00	37.00	28.00	21.00	8	19	27	7
109.00	15.00	38.00	28.00	23.00	9	20	29	6
105.00	16.00	35.00	28.00	23.00	10	14	23	6
106.00	17.00	32.00	30.00	24.00	8	15	23	8
100.00	16.00	32.00	28.00	24.00	8	19	27	6
103.00	16.00	38.00	31.00	25.00	9	20	29	6
113.00	17.00	34.00	29.00	25.00	8	12	19	6
108.00	15	35	30.00	23.00	8	15	23	6
108.00	16.00	33.00	28.00	22.00	8	19	27	6
105.00	16.00	33.00	28.00	22.00	8	15	23	7
105.00	17	32	30.00	21		16	26	6
109.00	15	32	28	25.00	9	18	27	6
107.00	15	34	d	24	8	22	30	5
112.00	17.00	33.00	31.00	22.00	11	26	37	6
103.00	17.00	33.00	29.00	24.00	10	22	32	7
113.00	18.00	35.00	30.00	23.00	10	20	30	6
109.00	17.00	37.00	32.00	24.00	10	17	27	6
113.00	18.00	35.00	29.00	21.00	8	17	25	6
105.00	16.00	33.00	29.00	22.00	12	16	28	5
94.00	18.00	37	34.00	24.00	8	18	26	6
101.00	16.00	30.00	31.00	25.00	14	23	37	7
101	15	32	31	24	8	12	20	6
115	16	33	28	26.00	8	20	28	6
103.00	16.00	35.00	29.00	22.00	8	16	26	6
103.00	14	31	31	23	8	18	26	7
	15	35	29	21.00				6
108.00	17	39	31	24.00	8			6
104.00	16	35	27	22	8	18	26	6
100.00	16.00	36.00	31.00	25.00	8	23	31	7
109.00	14	31	30.00	26	8	18	26	6
106.00	13.00	35.00	29	22	8	18	26	7
107.00	17	31	31	26	8	18	26	6
120.00	15	32	31	24	9	18	26	6
109	15	35	29	26	7	18	22	6

**112**	**15**	**32**	**31**	24	10	19	29	6
**101.00**	**16.00**	**34.00**	**29.00**	22.00	10	11	21	5
**100.00**	**16.00**	**34.00**	**28.00**	22.00	8	14	22	6/7
**102.00**	**15.00**	**36.00**	**28.00**	22.00	9	14	23	6/5
**108.00**	**15**	**36**	**28**	22	10	13	23	6/6
**101**	**16**	**33**	29	**24**	8	13	21	7
**94**	**15**	**35**	30	**20**	5	11	16	7
**95**	**14**	**32**	30	**21**	7	12	19	7
**97**	**14**	**32**	29	**20**	2	9	11	7
**99**	**14**	**34**	**29**	22	2	7	9	7
**96**	**16**	**33**	28	**21**	8	12	20	5
93.00	17.00	34.00	28.00	19.00	7.00	13.00	20.00	7.00

ridged subocs		number of upper labials		**upper labial to end of suboc**	***lower labials***	**supracilliaries**	supraoculars
6		8		7	**10**	9	7
5						10	6
5		9			**8**		
4/5	13	9/8	17	**7/9**	**8/8**	**9/9**	5
5/5	10	9/9	18	**8**	**7/7**	**10/9**	5
5/5	10	10/9	19	**8**	**8/8**	**9**	5
5/5	10	9/9	18	**7/7**	**8/7**	**9/9**	5
5/4	9	8/8	16	**8/6**	**8/8**	**10/11**	5
5/5	10	9/9	18	**7**	**8/8**	**9**	5
5/5	10	8/8	16	**8/8**	**7/7**	**9/10**	5
5/5	10	9/9	18	**5/7**	**8/8**	**9/9**	5
5/5	10				8/8	9/9	5
5	10	8/8	16	**7**	**7/7**	**9/8**	5
6		8	7	7	**n**	9	7
4/4	8	8/10	18	**8/7**	**7/7**	**9/d**	5
4/4	8	9/9	18	**8/8**	**7/7**	**9/d**	5
4/4	8	9/9	18	**7/7**	**8/7**	**41527**	6
**4/4**	**8**	**9/8**	**17**	**8/8**	**8/7**	**9/10**	**5**
**4/4**	**8**	**8/8**	**16**	**8/8**	**6/7**	**8/9**	**5**
d/d		d/d		**d/d**	**7/7**	**9/9**	5
3/3	6	9/9	18	**7/7**	**8/8**	**8/8**	5/5

3/3	6	9/10	19	7/8	7/7		8/10	5
4/4	8	9/9	18	8/8	6/7		10/9	5
4/4	8	9/9	18	7/7	8/8		9/9	5
3/4	7	9/9	18	7/7	8/8		9/9	5
4/4	8	9/9	17	8/8	7/7		d/9	d/d
4/4	8	10/9	19	8/8	9/9		10/9	6/5
4/4	8	9/9	18	8/8	7/7		9/9	6/7
4/4	8	9/9	18	7/7	7/8		10	5
4/4	8	8/8	16	8/8	7/7		10	5
4/4	8	9/9	18	7/7	7/7		10	4/5
4/4	8	10	19	7/8	8/8		9	5
4/4	8	8		7/8	7/7		9	5
4/4	8	10-Oct		8/9	7/7		9/10	5
4/4	8	9/8	17	8	8/8		10/9	5
4/4	8	8/8	16	8	7/7		11/10	5
5/4	9	8/8	16	8/8	7/8		10/9	5
4/5	9	9/9	18	8/7	8/7		10/10	5
4/4	8	9/9	18	7/7 (8/7)	8/9		10	5/6
4/4	8	10/10	20	7/8 (7/9)	8/9		9	5
4/4	8	10/10	20	9/8	7/8		9/10	5/6
3/3	6	10/10	20	7/7 (8/8)	8/7		8/9	6/5
3	6	10	10	7	8		10	5
5/5	10	9/9	18	7/7	8/8		10/10	5
4	8	9/9	18	8	7/7		9/9	5
4/4		10/10	20	8	8/7		10/9	5
5/5		9/10	19	7/8	7/7		10/10	6
5/5		10/9	19	7/7	8/8		41558	5
4/4		7/7	14	8/8	7/7		9/9	6
5/5		9/9	18	7/7	7/7		8/9	5
4/4		9/8	17	7/7	7/7		41557	5
3/3		9/9	18	8/8	8/8		10	7
4/4		10/9	19	7/7	8/8		9	5
5/5		10/11	21	9	8		9	5
4/4		9/9	18	8	7/7		9	5
4/4		8/8	16	7/7	8/8		9	5
4/4		11/9	20	8	7/8		9	5

4/4		9/10	19	**7/7**	**7/7**		**9**	5
5/5		7/8	15	**8**	**6/6**		**10**	5
5/5		8/8	16	**8**	**6/6**		**10**	5
5/4		8/7	15	**7/8**	**7/6**		**10**	5
5/5		8	16	**3-8**	**7/7**		**9**	5
4/4	8	8/8	16	**8/8**	**7/7**		**9/9**	5/5
4/4	8	9/9	18	**7/7**	**7/7**		**10/10**	6/6
3/4	7	9/9	18	**8/8**	**6/6**		**10/9**	5/5
4/3	7	9/9	18	**8/7**	**7/7**		**9/9**	5/5
4/4	8	8/8	16	**7/7**	**7/7**		**10/10**	5/5
4/4	8	8/7	15	**7/7**	**7/7**		**7/8**	6/6
4.00	8.00	9.00		**7.00**	**7.00**		**9.00**	5.00

longest UL	**shorest ul**	**tiny scales in suprocs**	subocs in contact with which upper labials		**pretemporals**	**rows of supratemporas**	**rows of temporals**	**chin shields**	**1s**
**14**	5	n	**3-7**	**5**	3	2	**11**	**6**	
						2			
34	2	**n**	3-7	1-4	**7/5**	2	**9**	**4**	
4	5	**y**	3-8		**4/4**	3	**9**	**4/4**	
3	2	**y**	3-8	1-4	**5-4**	3	**10**	**4**	
34	5	**y**	3-7		**4**	2		**4**	
34	12	**n**	3-6/3-7	1-3	**5/5**	3	**10**	**4**	
34	25	**y**	3-7	1-4	**3-3**	3	**10**	**4**	
4	1	**y**	3-8	1-3	**4/4**	4	**9**	**4**	
4	1	**n**	3-7/3-8	1-4	**5**	4	**10**	**4**	
**3**	**15**	**n**						4	
3	5	**N**	3-7		**4**	2	**10**	**4/4**	
**14**	5	n	**3-7**	**5**	3	2	**11**	**6**	
14	5	**x**	8		**4**	2	**10**	**5/4**	
3	5	**y**	7		**5**	2	**10**		
4	5	**x**	7		**4**	2	**8**	**4/4**	
**34**	**2**	**n**	**8**	**1-5**	**5/5**	**3**	**9**	**5/5**	
**4**	**2**	**x**	**8**		**4**	**2**	**9**	**4/4**	
34	2	**n**	5	1-3	**d/5**	2	**9**	**4/4**	
4	2	**y**	7		**5/5**	3/2	**9**	**4/4**	

4	2	n	8	1-4	5/5	2	9/9	4/4
4	2	n	8	1-4	5/5	2	9/9	4/4
4	2	n	8	1-4	5/5	2	9	4/4
4/34	2	n	8	1-3/1-4	5/5	3	9	4/4
34	2	n	8	1-4	d/d	2	9	5/5
34	2	n	8	1-4	5/6	1	9/9	4/4
34	2	n	8	1-4	5/5	1	9	4/4
34	2	n	7	1-4	4-4	2	9	5/5
34	2	n	8	1-4	5	2	9	5/5
3	2	n	7		4/4	2	10	6/6
3	2	n	7	1-4	4-3	2	8	5/5
34	2	n	7	1-4	3/4	2	8	5/5
1	2	x	8		4	2	9	4/4
4	2	x	8		5	2	9	4/4
4	2	x	8		5	2	9	5/4
34	2	n	8		4/5	2	11	5/5
34	2	n	8		5/6	2	11	4/4
		n	78	1-4	6/5	2	10	5/5
		n	78	1-4	6/6	2	9	5/5
		n	8	1-4	6/6	2	10	6/6
		n	8	1-3	6/7	2	8	6/5
3	2	n	8	1-2	4	2	11	5/5
34	2	n	8	1-5	4	2	9	5/4
3	5	n	8		3/4	2	9	4/4
4	5	y	8		4	2	10	4/4
4	5	x	7		5	2	9	4/4
4	25	x	7		6	2	10	5/5
4	5	x	7		5	2	10	4/4
4	2	x	8		4	2	10	4/4
4	25	x	8		5	2	9	4/5
3	5	x	7		5	2	10	5/5
14	5	x	7		5	2	9	5/5
3	2	x	9		5	2	9	5/5
4	2	x	7		5	2	10	4/4
4	5	x	7		5	2	9	5/5
4	25	x	8	4	2	2		3/4

4	25	**x**	7		**5**	2	**9**	**4/4**
4	15	**n**	3-8	1-5	**5-5**	3	**9**	**5/4**
4	5	**n**	3-8	1-5	**4-5**	3	**9**	**5/5**
4	5	**n**	3-7	1-4	**5-5**	2	**9**	**4/5**
4	5	**n**	7		**5**	2	**9**	**5/4**
4/4	5/5	**n**	3-8		**5/5**	3	**12**	**4/4**
3/4	5/5	**y**	3-7		**4/4**	2	**12**	**4/4**
4/4	5/5	**n**	3-8		**4/4**	2	**10**	**4/4**
4/4	5/5	**n**	3-8		**4/4**	3	**9**	**4/4**
4/4	5/5	**n**	3-8		**4/4**	2	**10**	**4/4**
4/4	2/2	**n**	3-7		**6/6**	1	**11**	**4/4**
4.00	5.00	**y**	3to7		**6.00**	2.00	**12.00**	**4.00**

**t chin shield contacts LL**	rows antegulars	rows of gulars	mesoptychials	dorsal pattern	**number windows in row**		**occipitals @ parieta;s**	rows of occipitals
23	**10**	x	x		3		5+	3
**2/1**	10	10	7	bands	**3**		**8**	3
**23/23**	7	8	9	bands	**2**		**4**	3
**2**	10	7	7	spots	**3**		**6**	3
**3/2**				spots	**d**		**5**	3
**2**	11	8	8	spots	**4**		**7**	3
**1/2**	10	9	9	spots	**3**		**6**	2
**2**	10	9	8	spots			**7**	3
**2/3**	11	10	7	mot/bands	**3**		**5**	3
2	9		8	bands	d		5	3
**2/2**	9	9	7	B/S	**3**		**7**	3
23	**10**	x	x		3		5+	3
**23/234**	8	10	4	lines	**d**		**4**	2
				lines	**d**		**4**	2
**23**	7	9	5	lines	**4**	**1**	**3**	2
**3**	**11**	**9**	**7**	**spots**	**15**	**2**	**3**	**3**
**23**	**9**	**8**	**5**	**uni**	**4**	**1**	**4**	**2**
**23**	10	9	9	lines	**d/d**		**5**	2
**23**	9	9	8	lines	**5**	**1**	**5**	3

**23**	**10**	**10**	**10**	**lines**	**4**	**1**	**4**	**3**
**23**	**8**	**9**	**9**	**lines**	**10**	**2**	**5**	**3**
**23**	**8**	**9**	**6**	**uniform**	**12**	**2**	**5**	**2**
**23**	**8**	**9**	**8**	**spots**	**6**	**1**	**5**	**3**
**2**		**9**	**9**	**lines**	**15**	**2**	**5**	**3**
**2**	**8**	**8**	**9**	**bands**	**14**	**2**	**5**	**3**
**2**	**9**	**9**	**9**	**bands**	**15**	**2**	**6**	**2**
**2**	10	10	6	bands	**13**	**2**	**8**	3
**23**	12	10	8	bands	**10**	**2**	**6**	3
**3/2**	9	10	7	bands	**d**		**5**	2
**2**			8	bands	**13**	**2**	**5**	2
**2**	10	9	8	lines	**15**	**2**	**6**	3
**23**	8	9	4	uni	**8**	**2**	**5**	2
**23**	11	10	4	bands	**3**	**1**	**5**	2
**23**	10	9	4	bands	**12**	**2**	**5**	2
**2**	8	9	6	bands	**14**	**2**	**5**	2
**2**	11	10	6	bands	**13**	**2**	**5**	3
	8	10	13 (6)		**11**	**2**	**7**	3
	8	11	12 (6)		**15**	**2**	**8**	3
	9	10	13 (7)		**10**	**2**	**6**	3
	10	9	12(7)		**11**	**2**	**8**	3
**23/23**	11	9	9	bands	**6**	**1**	**10**	2-3
**2**	11	10	8	lines	**4**	**1**	**7**	3
**23/23**	8	9	8	bands	**3**	**1**	**7**	3
**23/23**	8	10	5	uni	**3**		**4**	3
**23**	12	9	4	lines	**3**		**4**	d
**23**	12	9	5	reticulate	**4**		**3**	3
**23**	7	9	5	lines	**3**		**5**	3
**23**	11	10	4	uni	**3/1**		**5**	3
**23**	9	9	4	uni	**10**		**5**	3
**23**	10	10	5	lines	**3**		**4**	3
**23**	11	9	4	lines/sp	**4**		**5**	3
**23**	10	8	5	lines	**3**		**4**	2
**23**	10	9	5	lines	**d**		**5**	2
**23**	9	9	5	lines	0		**4**	**5**
**2/2-3**	12	9	5	bands	0		**4**	**6**

**23**	9	9	5	lines	**3**		**3**	3
**23**			9	irelines	**5**		**5**	3
**23**	10	9	8	spots	**d**		**5**	2
**23**	9	9	9	spots	**6**		**4**	2
23	**10**	**9**	9	spots	5		5	**2**
**4/5**	9	8	4	bands,spots	2		**4**	**7**
**23/3**	9	9	5	bands,spots	2		**3**	**3**
**23/23**	9	8	4	bands,spots	2		**3**	**3**
**34/34**	12	5	5	bands,spots	2		**2**	**5**
**34/34**	12	6	5	bands,spots	2		**3**	**5**
**23/23**	10	11	6	bands,spots	**3**		**5**	3
**23.00**	12.00	7.00	5.00	ba	**4.00**		**3.00**	2.00

numberofoccipitals	prs cs @ labials
23+	2
**23**	2
**19**	2
**18**	2
**14**	2
**21**	2
**19**	2
**20**	2
**18**	2
15	2
**16**	2
23+	2
**9**	2
**10**	23
**9**	23
**15**	**2**
**12**	**23**
**11**	1/2
**21**	2

**15**	**3/3**	
**15**	**2**	
**13**	**2**	
**20**	**2**	
**21**	**2**	
**20**	**2**	
**18**	**2**	
**14**	2	
**12**	2	
**14**	2	
**10**	2	
**9**	2	
**15**		
**9**	23	
**11**	23	
**17**	2	
**23**	2	
**30**	2	
**30**	2	
**26**	2	
**28**	2	
**29**	2	
**23**	2	
**17**	2	
**15**	23	
**d**	23	
**13**	23	
**14**	23	
**14**	23	
**20**	23	
**13**	23	
**17**	23	
**11**	23	
**13**	23	
3	**12**	23
2	**15**	2/2

**11**	23	
**13**	2	
**13**	2	
**14**	2	
**11**	23	
2	**14**	3/3
2	**11**	
2	**7**	
3	**13**	
3	**13**	
**24**	2/2	
**9.00**	2.00	
