# Supplementary figures and images for: Cryptic, Sympatric Diversity in Tegu Lizards of the Tupinambis teguixin Group (Squamata, Sauria, Teiidae) and the Description of Three New Species
Source: PLoS One. 2016 Aug 3;11(8):e0158542. doi: 10.1371/journal.pone.0158542 (PMC4972348; doi:10.1371/journal.pone.0158542)

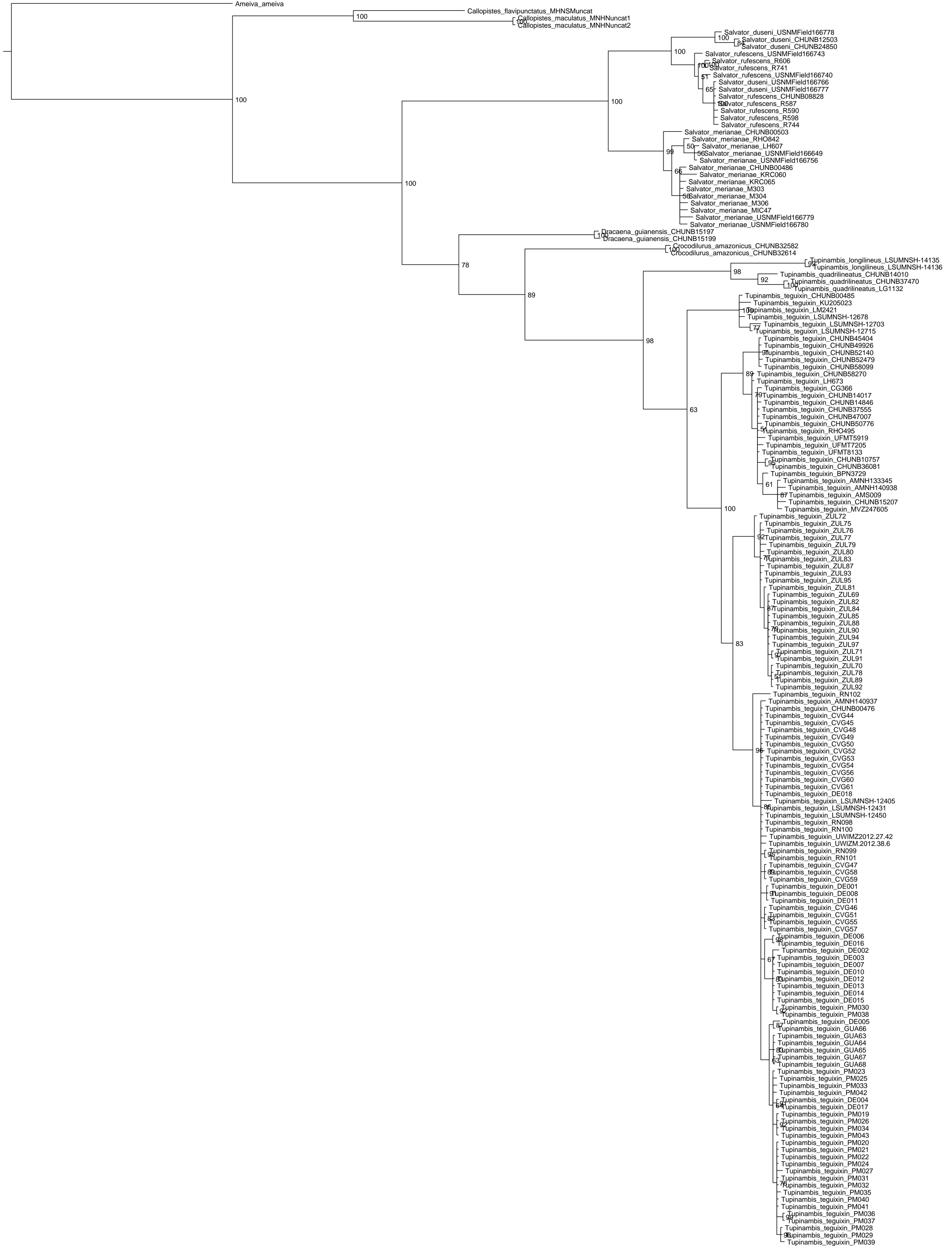

Supplement: S1 Fig — (PDF) [file pone.0158542.s003.pdf]
