# Supplementary material for: Cryptic, Sympatric Diversity in Tegu Lizards of the Tupinambis teguixin Group (Squamata, Sauria, Teiidae) and the Description of Three New Species
Source: PLoS One. 2016 Aug 3;11(8):e0158542. doi: 10.1371/journal.pone.0158542 (PMC4972348; doi:10.1371/journal.pone.0158542)
Supplement: S2 Fig — (PDF) [file pone.0158542.s004.pdf]

Supplemental Information 2 Figures.

S2 Fig. 2A. cluster analyses B. PCA for 61 specimens. The same data set was used for both method. Blue is *Tupinambis cryptus*, brown is *T. zuliensis*, red is *T. teguixin*, and green is *T. cuzcoensis*. The blue box in the PCA denotes a Peruvian specimen of T. cuzcoensis with a high vertebral count. Table S2 provides the eigenvalues and loadings for PCA Fig2B.

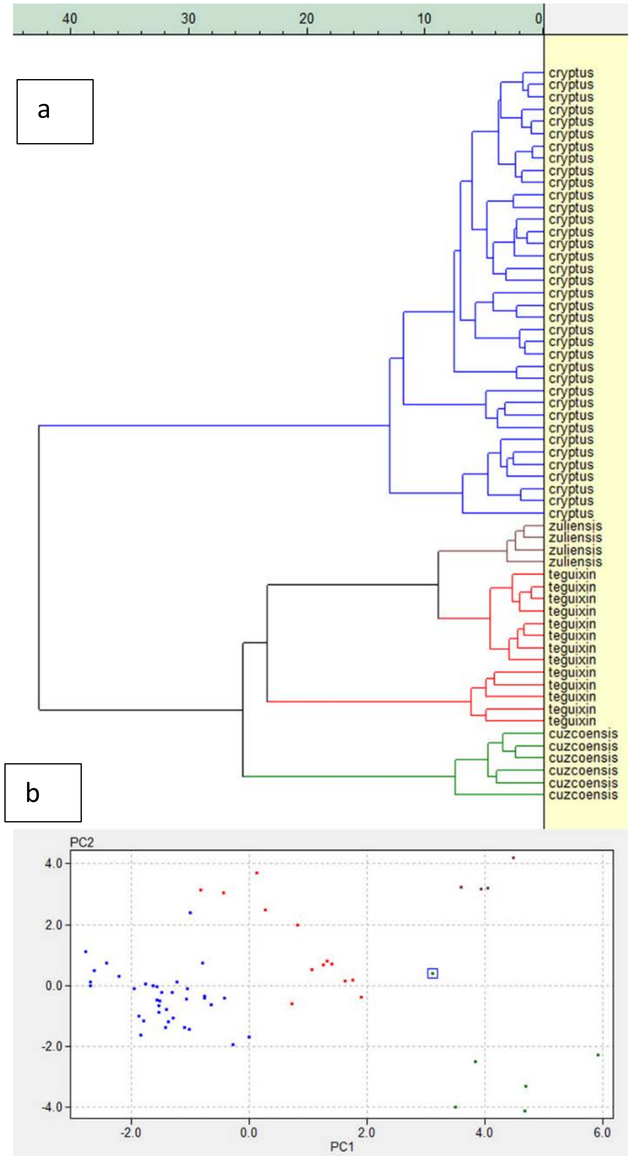
a
40
30
20
10
0
cryptus
zuliensis
teguixin
cuzcoensis
b
PC2
4.0
2.0
0.0
-2.0
-4.0
-2.0
0.0
2.0
4.0
6.0
PC1

Eigenvalues and Loadings for PCA B.

Eigenvalues	PC-1	PC-2	PC-3	PC-4	PC-5	PC-6	PC-7	PC-8	PC-9	PC-10	PC-11	PC-12	PC-13	PC-14	PC-15	PC-16	PC-17	PC-18	PC-19	PC-20	PC-21
	4.923	3.072	2.357	1.542	1.467	1.171	1	0.9543	0.8642	0.6627	0.585	0.4931	0.4092	0.3481	0.3284	0.2745	0.1834	0.1578	0.1424	0.06462	1.07E-16
	loadings																				
	PC-1	PC-2	PC-3	PC-4	PC-5	PC-6	PC-7	PC-8	PC-9	PC-10	PC-11	PC-12	PC-13	PC-14	PC-15	PC-16	PC-17	PC-18	PC-19	PC-20	PC-21
longest supr:	0.3018	0.1078	0.1087	0.403	-0.1325	0.00451	0	-0.1487	0.2121	0.01627	-0.1177	0.0113	-0.3619	-0.2084	0.07812	0.3728	0.05941	-0.3661	-0.1671	-0.3731	4.76E-16
largest supp:	0	0	0	0	0	0	1	0	0	0	0	0	0	0	0	0	0	0	0	0	0
occipitalIP:	0.2027	0.2841	-0.07355	-0.1992	-0.04852	0.2698	0	0.3915	-0.06946	0.03004	0.341	0.154	-0.00608	-0.6263	0.2322	-0.05975	0.02572	-0.0079	0.07262	0.05665	-9.36E-17
orbit c/upp :	-0.2987	-0.1765	0.1763	0.2771	0.08921	-0.1396	0	-0.2118	0.01863	0.1369	-0.1158	-0.159	-0.07983	-0.5093	0.1071	-0.3168	0.2116	0.3986	0.146	-0.1976	-3.10E-16
number of su:	0.3306	-0.185	-0.01437	-0.2108	-0.1528	-0.2095	0	0.05038	0.148	0.04607	-0.01797	0.2159	-0.0412	0.2476	0.4791	0.2042	0.1105	0.4257	0.3082	-0.2201	-1.64E-16
supracil @ l:	-0.2905	-0.06317	-0.1052	0.05416	-0.00944	-0.4871	0	0.1326	-0.01621	0.4005	0.174	0.2407	-0.1712	0.04175	0.3671	-0.1464	-0.2097	-0.2503	-0.3	0.09981	7.89E-17
vertebral ro:	0.2863	0.07915	0.3364	-0.1628	0.1167	-0.0994	0	-0.00996	-0.1968	-0.1737	-0.3186	-0.1139	-0.05289	-0.02387	0.1768	-0.3975	-0.5545	-0.1559	0.1143	-0.1664	5.64E-16
scales aroun:	-0.2655	0.02324	-0.1971	-0.08741	0.3303	0.06102	0	-0.00263	-0.1472	-0.3585	-0.441	0.4945	-0.2091	-0.06015	0.1509	0.04561	0.2462	-0.1728	0.1258	0.04535	-1.36E-16
lamella 4th :	-0.163	0.2499	0.1945	0.1884	-0.1797	-0.2064	0	-0.1423	0.3486	-0.4181	0.3465	0.4261	0.1759	0.02119	-0.1391	-0.1608	-0.1433	-0.00399	0.2026	-0.07979	8.71E-17
lamellae 4th:	-0.00678	0.155	-0.1247	0.2114	-0.3992	-0.4176	0	0.4041	-0.3549	-0.1568	-0.3068	-0.01156	0.06174	-0.1336	-0.2423	0.1904	-0.07212	0.225	-0.02378	0.02747	-2.99E-17
transverse v:	-0.03695	-0.226	-0.3679	-0.09084	-0.3422	0.3023	0	-0.2246	0.202	0.05368	-0.1704	0.2224	-0.2674	-0.1573	-0.1509	-0.06926	-0.4802	0.2313	-0.09456	0.07729	-6.24E-17
longitudinal:	-0.1468	0.2566	0.3986	-0.00256	0.2928	0.01259	0	0.05237	0.01142	0.215	0.04837	0.05645	-0.4118	0.03438	-0.1515	0.3919	-0.2581	0.2714	0.2125	0.2838	-4.36E-16
total pores:	-0.3209	0.1138	-0.09051	0.2427	-0.02293	0.2269	0	0.0594	0.1872	0.0568	-0.2257	-0.1872	0.4715	-0.0251	0.428	0.2917	-0.3016	-0.0855	0.2128	0.04902	4.03E-16
number of su:	0.07194	-0.2089	0.2582	0.01614	0.03714	0.04934	0	0.5483	0.5625	0.1443	-0.339	0.1267	0.07069	-6.67E-05	-0.192	-0.1934	0.1178	-0.06483	-0.0598	0.1035	-1.44E-16
total UL:	-0.2098	-0.2895	-0.1064	0.008198	-0.03581	-0.02712	0	0.3154	0.1565	-0.4897	0.2436	-0.4496	-0.4323	0.0472	0.1308	0.01431	-0.06497	-0.07443	0.129	0.02792	9.31E-17
supraoculars:	0.01495	-0.04043	0.1195	0.527	-0.1895	0.4434	0	0.1924	-0.3417	0.09282	0.08556	0.1896	-0.164	0.3562	0.1255	-0.2818	0.05285	0.05216	0.116	-0.00093	-1.15E-16
occipitals @:	-0.1474	0.2957	0.007214	-0.2584	-0.4856	-0.04386	0	-0.1335	0.06908	0.244	-0.1655	-0.1689	-0.2064	0.06225	-0.02622	-0.169	0.2491	-0.301	0.4486	0.1366	1.42E-17
numberofocci:	-0.1626	0.3638	0.2596	-0.164	-0.2181	0.1414	0	-0.05299	0.1062	-0.2247	-0.1386	-0.09918	-0.08247	0.1194	0.3082	-0.06361	0.1532	0.2844	-0.5944	0.0896	-5.16E-16
postmental l:	0.2929	-0.2881	0.1977	0.1764	-0.1326	-0.1105	0	-0.2468	-0.04523	-0.1669	-0.02121	0.03067	0.06842	-0.1765	0.1888	0.05434	0.07748	-0.09135	0.01565	0.7416	-5.48E-16
femoral scal:	-0.2198	-0.3028	0.3419	-0.2114	-0.2138	0.0977	0	0.01794	-0.1844	-0.00446	0.04848	0.1141	0.08415	-0.08508	-0.03317	0.1945	-0.01468	-0.1256	-0.03268	-0.152	0.7071

adult pattern:	0.2198	0.3028	-0.3419	0.2114	0.2138	-0.0977	0	-0.01794	0.1844	0.004455	-0.04848	-0.1141	-0.08415	0.08508	0.03317	-0.1945	0.01468	0.1256	0.03268	0.152	0.7071
