# Supplementary material for: Cryptic, Sympatric Diversity in Tegu Lizards of the Tupinambis teguixin Group (Squamata, Sauria, Teiidae) and the Description of Three New Species
Source: PLoS One. 2016 Aug 3;11(8):e0158542. doi: 10.1371/journal.pone.0158542 (PMC4972348; doi:10.1371/journal.pone.0158542)
Supplement: S3 Fig — (PDF) [file pone.0158542.s005.pdf]

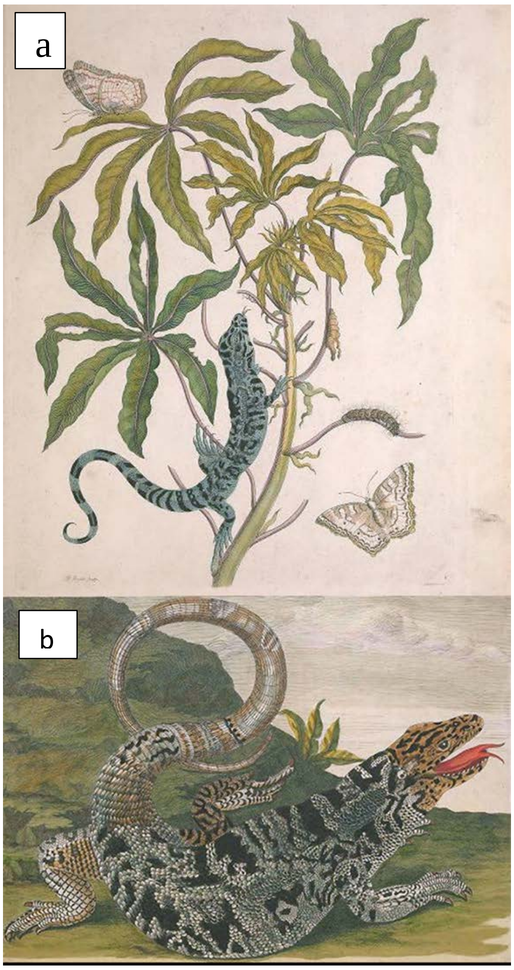

Historic illustrations of members of the *Tupinambis teguixin* group. Plate 70 top (a) from Merian [60] and the lectotype of *Lacerta monitor* Daudin [61]. Plate 4 bottom (b) from Merian [60].

Supporting Information 3. The confusion of names for *Tupinambis* species began in the 18th century. Plate 70 (a) from Merian [60] and the lectotype of *Lacerta monitor* Daudin [61]. Plate 4 (b) from Merian [60]. Given the pattern of the animal depicted in Plate 70 it probably represents *Tupinambis teguixin* as defined here (wide black bands with narrow light ones). Plate 4 [60] likely represents a specimens of *Tupinambis cryptus*, given the yellow head and chin, indistinct bands on the dorsum and the pattern present on the hind legs. For a discussion of the dates of Merian's work see Valiant [62]. *Lacerta monitor* Linnaeus [63] is a name with a long and complex history. Avila-Pires [10] noted this name was used in reports by Merian [60] and plates 70 and 4 depict specimens of *Tupinambis* from Suriname. Plate 70 of Merrem was designated the lectotype of *Lacerta monitor* by Avila-Pires [10] and therefore a junior synonym of *Lacerta teguixin*. Shaw and Nodder [64] applied *Lacerta monitor* to a lizard that appears to be *Varanus scalaris.* Latreille [65] also used this name for a *Tupinambis*. Mertens [66] placed *Tupinambis monitor* Daudin [61] in the synonymy of *Tupinambis teguixin*, following Spix [67]. In 1957 the ICZN (opinion 540) [68] declared *Lacerta monitor* Linnaeus *nomen rejectum* and thus is unavailable. However, the name continues to appear in synonymies of both *Varanus* and *Tupinambis*. Given the dorsal pattern and the Suriname origin it seems likely Merian's [60] plate 4 is based upon *T. cryptus*. Plate XIX in Spix [68] labeled "*Tupinambis monitor*" shows a tegu with a divided loreal and a row of granular scales separating the supraoculars from the ciliaries, traits diagnostic of *Salvator*. Duméril and Bibron's [69] description of "*Salvator merianae*" is more in agreement with *Lacerta teguixin* than it is with *Salvator merianae*. Avila-Pires [10] summarized and clarified much of the confusion that existed between *Tupinambis teguixin* and *Salvator merianae* in the literature from the 18th to the late 20th century, but the confusion has clearly continued into the 21st century.

References

60. Merian MS. Over de Voortteeling en Wonderbaerlyke Veranderingen der Surinaemsche Insecten, Waer in de Surinaemsche Rupsen en Wormen, met alle derzelver Veranderingen, naer het leeven afgebeeldt, en beschreeven worden; zijnde elk geplaest op deselfde Gewassen, Bloemen, en Vruchten, daer ze op gevonden zijn; Beneffens de Beschrijving dier Gewassen. Waer in ook de wonderbare Padden, Hagedissen, Slangen, Spinnen, en andere zeltzame Gediertens worden vertoont, en beschreeven. Alles in Amerika door den zelve M.S. Meiraen naer het leven, en levensgrootte Geschildert, en nu in't Koper overgebracht. Benevens een Aenhangsel van de Veranderingen van Visschen in Kikvorschen, en van Kikvorschen in Visschen: 8 pp., 1–72, Amsterdam 1719, 1730.

61. Daudin FM, Sonnini CS. Histoire naturelle, générale et particulière des reptiles: ouvrage faisant suite à l'histoire naturelle générale et particulieère, composée par Leclerc de Buffon, et rédigée par CS Sonnini, membre de plusieurs sociétés savantes. Dufart; 1801-1803.

62. Valiant S. Maria Sibylla Merian: recovering an eighteenth-century legend. Eighteenth Century Studies. 1993 Apr 1:467-79.

63. Linnaeus C. Systema naturæ per regna tria naturæ, secundum classes, ordines, genera, species, cum characteribus, differentiis, synonymis, locis. Tomus I. Editio decima, reformata. Laurentii Salvii, Holmiæ. 10th Edition, 824 pp 1758.

64. Shaw G, Nodder FP (eds.) *The Naturalist's Miscellany* [...], Vol. I. London, Nodder & Co., plates 1-37, 158 unnumbered pages August 1, 1789, and July 1, 1790. 63. Latreille PA. In: Sonnini CS, Latreille PA Histoire naturelle des reptiles: avec figures dessinées d'apres nature Vol. 1, 280 pp. Paris. 1801.

65. Latreille PA. In: Sonnini CS, Latreille PA Histoire naturelle des reptiles: avec figures dessinées d'apres nature Vol. 1, 280 pp. Paris. 1801.

66. Mertens R. Amphibien und Reptilien I. Ausbeute der Hamburger Südperu-Expedition. In: Titschack E (ed.), Beiträge zur Fauna Perus, vol. 2. 1942. Hamburg, pp. 277-287.

67. Spix JB. Animalia Nova sive species novae Lacertarum, quas in itinere per Brasiliam, annis 1817-1820 jussu et auspiciés Maximiliani Josephi 1. Fr. S. Hübschmanni. 26 pp. 1825.

68. ICZN Opinion 540. Protection under the plenary power of the specific names *bengalensis* Daudin, [1802], as published in the combination *Tupinambis bengalensis*, and *salvator* Laurenti 1768, as published in the combination *Stellio salvator*. 1959. Opin. Declar. Intern. Com. Zool. Nom. 20: 77-85.

69. Duméril AMC, Bibron G. Erpétologie Générale on Histoire Naturelle Complète des Reptiles. Vol.5. Roret/Fain et Thunot, Paris, 871pp. 1839
